# A fast and semi-fragile high-capacity digital watermarking system for MRI images using a hybrid combination of DOST and LBP features

**DOI:** 10.1038/s41598-026-50380-x

**Published:** 2026-05-05

**Authors:** Nimmy George, Michael George, Manju Manuel

**Affiliations:** 1 Department of Electronics and Communication Engineering, Rajiv Gandhi Institute of Technology, Kottayam, Kerala 686501 India; 2 Department of Electronics and Communication Engineering, Government Engineering College, Painavu, Idukki, 685603 India; 3 Affiliated to APJ Abdul Kalam Technological University, Kerala, India

**Keywords:** Watermarking, Stockwell transform, Local binary pattern, Embedding, Electronic patient record, Extraction, Cancer, Computational biology and bioinformatics, Engineering, Mathematics and computing

## Abstract

In telemedicine, digital watermarking is critical for safeguarding patient privacy by securely embedding sensitive Electronic Patient Records (EPR) directly into medical imagery. This work proposes a high-capacity, non-blind, and semi-fragile watermarking scheme leveraging a 64-bit complex-float computational pipeline and the Discrete Orthonormal Stockwell Transform (DOST) combined with Local Binary Pattern (LBP) features. To improve security, the architecture incorporates Arnold scrambling for confusion and chaotic mapping for diffusion-based encryption. In the attack-free scenario, the proposed method significantly outperformed the recent state-of-the-art (SOTA) technique across two MRI datasets. On the TCIA MRI dataset, the proposed method achieved mean values of 79.84 dB for Peak Signal-to-Noise Ratio (PSNR) and 0.9969 for Structural Similarity Index (SSIM), representing average improvements of 111.61$\%$ and 4.35$\%$, respectively, over the SOTA baseline (37.73 dB and 0.9553). On the BMIBTD MRI dataset, the proposed method achieved mean values of 65.62 dB for PSNR and 0.9827 for SSIM, reflecting average improvements of 77.54$\%$ in PSNR and 11.14$\%$ in SSIM compared to the SOTA values (36.96 dB and 0.8842). Furthermore, the proposed method maintained a mean Normalised Correlation Coefficient (NCC) of 1, demonstrating performance parity with the SOTA technique in terms of general robustness. Extensive robustness analysis reveals a selective, tiered sensitivity profile: the scheme exhibits high resilience to stochastic channel noise (NCC$\gtrsim$0.90), a characteristic semi-fragile response to Median filtering (NCC$\approx$0.6–0.8), and high sensitivity to deterministic manipulations such as JPEG compression, Mean filtering, and geometric transformations. Rather than a limitation, this tiered behaviour serves as an inherent integrity-authentication mechanism, where the degradation or destruction of the mantissa-embedded watermark provides immediate detection of unauthorised spatial tampering or resampling post-acquisition. Computational time analysis demonstrates that the proposed method maintains an efficient total mean execution time of 0.2203 seconds, with separate mean processing times of 0.1293 seconds for embedding and 0.0910 seconds for extraction, ensuring rapid performance for real-time clinical applications. The capacity (8.00 bpp) and efficiency ($\eta$ = 0.0625) of the proposed technique were analysed and found to be high compared to recent SOTA methods. Security analysis confirms the proposed framework’s robustness against statistical, differential, and brute-force attacks, achieving superior performance metrics—including high NPCR and information entropy—compared to recent SOTA methods. Beyond MRI datasets, the system demonstrated comparable performance when validated on X-ray and ultrasound scans, confirming the framework’s cross-modality consistency. The proposed system provides a significant advancement in balancing high-fidelity diagnostic reconstruction with robust defence against cryptanalytic threats.

**Supplementary Information:**

The online version contains supplementary material available at 10.1038/s41598-026-50380-x.

## Introduction

Telemedicine refers to the real-time, remote provision of medical, diagnostic, and treatment services via technology, allowing patients to access healthcare from anywhere with an internet connection, thus avoiding the need for in-person consultations^1^. The use of telemedicine has surged in recent years because of its low cost and the risks associated with physical visits. The transmission of medical images is a vital cog of telemedicine, and the security of this communication is vital.

Digital watermarking techniques^2^, are crucial in health data management systems for safeguarding the confidentiality of medical information, controlling access and retrieval, and ensuring data integrity. By embedding watermarks containing medical details, such as patient records, hospital signatures, and diagnostic information into medical images, the data can be securely shared over networks. Since the quality of medical images is vital for accurate diagnosis, the watermark embedding process must be handled with extreme care.

A novel digital watermarking system for telemedicine applications using Discrete Orthonormal Stockwell Transform (DOST) is presented in this article. The DOST^3^ allows a fast and compact representation of image energy. The major highlights of this paper are as follows:

1. Watermark embedding is executed within a 64-bit complex-float DOST computational pipeline, utilising LBP features to optimise the embedding factor for maximum imperceptibility. This high-precision approach ensures a semi-fragile architecture: providing resilient recovery of EPR under transmission noise, while facilitating immediate tamper-detection through sensitivity to geometric and lossy manipulations.
2. The system integrates Arnold scrambling and chaotic mapping to provide joint confusion-diffusion encryption, ensuring high resistance to statistical and differential cryptanalysis while protecting sensitive patient metadata (EPR).
3. The imperceptibility, watermark robustness, robustness against attacks, time, capacity, and security performance of the system are extensively analysed against other recent SOTA techniques on MRI, X-ray, and Ultrasound datasets.

The rest of the article includes sections on the literature survey, the methods based on which the proposed system is built, the proposed watermark embedding, the watermark extraction, the experimental results, the discussions, and the conclusions.

**Table 1:** Summary of survey of recent literature on transform domain techniques for medical image watermarking

References	Year	Techniques (E: Embedding, S: Security)	Remarks	Cap.
^4^	2023	**E:** DWT + LWT **S:** Arnold scrambling	Semi-blind; Fingerprint watermark; LBP features decide embedding factor; Pro: Speed.	0.0625
^5^	2025	**E:** DWT + HMD + SVD **S:** Arnold scrambling	Semi-blind; Binary watermark in singular value domain; Pro: Speed.	0.0156
^6^	2025	**E:** VQ + PVD **S:** Hyperchaotic encryption	Blind; Colour images; Pro: High capacity; Con: Processing time.	3.5500
^7^	2025	**E:** PHOT + VGG19 **S:** Chirikov map & Fibonacci Q-matrix	Blind; Colour images; Con: Processing time.	0.0052
^8^	2025	**E:** MD-FFT **S:** LFSR, LZW, & Arnold scrambling	Blind; Colour images; Binary watermark.	0.0052
^9^	2025	**E:** AlexNet + WST + Histogram **S:** Fractal encryption	Blind; Binary watermark; Con: Processing time.	0.0156
^10^	2023	**E:** DWT + HQR **S:** Arnold scrambling	Blind; Medical video; Con: Processing time.	0.0208
^11^	2023	**E:** Slant + SVD + QFT **S:** OTP & Arnold scrambling	Blind; Binary watermark; Con: Processing time.	0.0052
^12^	2022	**E:** DWT **S:** Patient photo encrypted, patient info hashed	Blind; Con: Processing time.	0.3152
^13^	2021	**E:** IWT **S:** Chaotic map, SHA256	Blind; Pro: Good capacity; Con: Processing time.	2.2400
^14^	2020	**E:** NROI + DCT **S:** –	Blind; Deep network for ROI; Variable capacity.	Var.

DWT, discrete wavelet transform; LWT, lifting wavelet transform; LBP, local binary pattern; HMD, Hessenberg matrix decomposition; SVD, singular value decomposition; VQ, vector quantisation; PVD, pixel value differencing; PHOT, phase-only transform; VGG19, visual geometry group (19 layers); MD-FFT, multi-dimensional fast fourier transform; LFSR, linear feedback shift register; LZW, Lempel-Ziv-Welch; HQR, Hessenberg-QR; QFT, Quaternion fourier-transform; OTP, One-Time Padding; WST, WaterShed transform; IWT, integer wavelet transform; NROI, non-region of interest; ROI, region of interest; DCT, discrete cosine transform; Cap., capacity (bits per pixel)

## Literature survey

The classification of digital watermarking—blind, semi-blind, and non-blind^4^—reflects a fundamental trade-off between extraction autonomy and data robustness. While blind schemes are preferred for general media, they frequently suffer from ”host-interference,” where the host image acts as noise during extraction. In contrast, non-blind frameworks utilise the host data as a reference to achieve a mathematically clean subtraction, making them highly efficacious for closed-loop medical environments where data integrity is non-negotiable. Another means of classification is the domain of watermark embedding, with spatial domain^15,16^ and transform domain methods. Kadian et al.^17^ presents a review of digital watermarking with special focus on transform domain methods. Transform domain methods^18–28^ have better robustness against attacks and have greater capacity. Traditional transform-domain methods (using DWT, SVD e.t.c) face a ”precision-capacity” bottleneck. For instance, DWT-based methods often cause boundary artefacts during high-capacity embedding due to the loss of phase information, while SVD-based methods are computationally expensive. As summarised in Table 1, existing transform domain medical image watermarking works struggle to balance high embedding capacity with real-time processing speeds. To address these gaps, we propose a scheme in the DOST domain. Unlike the DWT, the DOST provides orthonormality and localised spectral analysis^3^, allowing for a ”tighter” embedding of EPR data without spreading distortion across the entire image. By leveraging its FFT-based structure^29^, the proposed method achieves the capacity of a non-blind system with the computational efficiency required for modern telemedicine.

## Methods used

In the proposed non-blind transform based scheme for digital watermarking, local binary pattern and discrete orthonormal Stockwell transform are the methods used for watermark embedding. The secrecy and security are ensured using Arnold transform based scrambling and chaotic diffusion mapping. These methods are briefly explained below.

### Local binary pattern (LBP)

Local Binary Pattern generates a binary code by comparing each pixel intensity with the pixel intensity of the central value of the neighbourhood. If the pixel intensity is greater than the central pixel value, then a 1 is assigned, and 0, otherwise^30^. This is mathematically represented as given below

1 $$\begin{aligned} LBP_{P,R}=\sum _{p=0}^{P-1}v(g_{p}-g_{c})2^{P} \end{aligned}$$

where P is the number of neighbouring pixels, $g_{p}$ is the grey level of the neighbouring pixel, $g_{c}$ is the grey value of the central pixel. Here,

2 $$\begin{aligned} v(x) = {\left\{ \begin{array}{ll} 1 & \text {if } x \ge 0 \\ 0 & \text {otherwise} \end{array}\right. } \end{aligned}$$

LBP is highly effective in texture description, making it useful for watermarking applications where texture information is key for embedding and extracting watermarks^31^. LBP is a simple and efficient method with low computational complexity, making it suitable for real-time digital watermarking systems. By focusing on local patterns, LBP captures fine-grained details within small image regions. This can improve the watermark’s imperceptibility, as embedding can be done in regions that are less noticeable to human eyes.

### Discrete orthonormal stockwell transform (DOST)

The continuous Stockwell (S) transform of a function v(t) is given as

3 $$\begin{aligned} S(t,f)=\int _{\infty }^{\infty }v(t)\frac{|f|}{\sqrt{2\pi }}exp^{-\frac{(\tau -t)^{2}f^{2}}{2}}exp^{-i2\pi ft}dt \end{aligned}$$

where $S(t,f_{0})$ is a one-dimensional function of space for a constant frequency $f_{0}$, which means that the signal describes how the amplitude and phase of the signal change over space, but only for that specific frequency $f_{0}$. The discrete Stockwell transform is given as

4 $$\begin{aligned} S\left( jT,\frac{n}{NT} \right) =\sum _{m=0}^{N-1}V\left( \frac{m+n}{NT} \right) \left( exp\frac{2\pi ^{2}m^{2}}{n^{2}} \right) exp\left( \frac{i2\pi mj}{N} \right) \end{aligned}$$

where j, m and n=0,1,........N-1. The Discrete Orthonormal Stockwell Transform is defined as

5 $$\begin{aligned} S\{ v[kT] \}=\sum _{k=0}^{N-1}v[kT]S_{[\nu ,\beta ,\tau ]}kT \end{aligned}$$

where *v*[*kT*] is the discrete signal, k is the spatial index, T is the sampling period, $\nu$ is the frequency variable, $\beta$ is the width of the frequency band, and $\tau$ is the spatial localisation parameter indicating the position in space where the analysis is focused.

### Arnold transform

The Arnold Transform, also known as the 2D Discrete Chaotic Cat Map, is utilised to scramble the spatial coordinates of pixels in the watermarked host image. For a square image of size $N \times N$, the transformation is defined as:

6 $$\begin{aligned} \begin{bmatrix} x' \\ y' \end{bmatrix} = \begin{bmatrix} 1 & p \\ q & pq + 1 \end{bmatrix} \begin{bmatrix} x \\ y \end{bmatrix} \pmod N \end{aligned}$$

where:

- (*x*, *y*) represents the original pixel coordinates.
- $(x', y')$ represents the new scrambled coordinates.
- *p* and *q* are positive integers acting as control parameters (secret keys).
- *N* is the dimension of the image.

The inverse transform is applied during extraction to restore the original spatial arrangement of the watermarked host image.

### Chaotic diffusion encryption

The diffusion process transforms a plain signal $\textbf{S}$ into a cyphered signal $\textbf{S}'$ by injecting pseudo-random noise generated from a non-linear dynamical system. Here, the chaotic generator used is the Logistic Map, which is defined by the iterative equation:

7 $$\begin{aligned} x_{n+1} = r \cdot x_n(1 - x_n) \end{aligned}$$

where $x_n \in (0,1)$ represents the chaotic state at step *n*, and $r \in [3.57, 4]$ is the control parameter ensuring the system operates in a chaotic regime. These $x_n$ is used to generate a chaotic sequence $\textbf{C}$, which is used with the signal $\textbf{S}$ as defined below:

- **Encryption:** $$\textbf{S}_{enc} = \textbf{S} + \textbf{C}$$
- **Decryption:** $$\textbf{S} = \textbf{S}_{enc} - \textbf{C}$$

This method ensures that the statistical distribution of the original signal is flattened across the range of the chaotic map and provides a high level of security against brute-force attacks while maintaining the numerical precision of the original signal.

## Proposed technique for digital watermarking embedding using discrete orthonormal stockwell transform

**Fig. 1:**
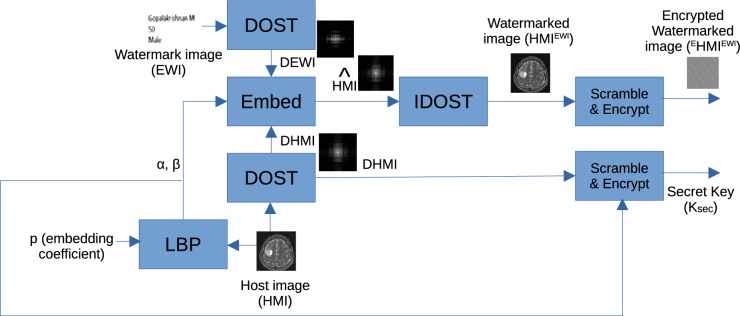
The block diagram of the proposed watermark embedding

A semi-fragile digital watermark embedding technique (block diagram shown in Fig. 1) for MRI images using Local Binary Pattern (LBP) features and the Discrete Orthonormal Stockwell Transform (DOST) is presented in this section. The LBP features extract texture information, while DOST provides an efficient space-frequency representation. With the aim of improving capacity, the watermark is embedded in all the DOST subbands of the host MRI image while promising general robustness and imperceptibility. Also, to ensure security during transmission, the watermarked images are scrambled using the Arnold transform and encrypted using chaotic diffusion. Also, for added security, the keys for watermark extraction are scrambled and encrypted. Algorithm 1 lists the steps used for the watermark embedding and encryption process.

**Algorithm 1:**
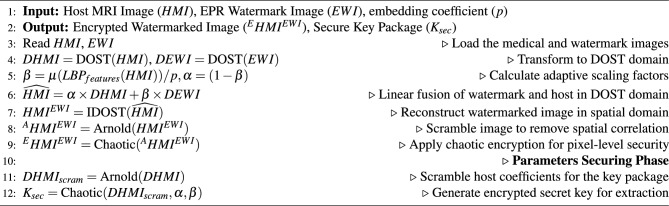
Proposed Secure Watermark Embedding Algorithm

### Algorithm 1 elaboration: embedding process

The proposed embedding framework is designed to achieve high payload capacity while maintaining multi-layer security through a combination of frequency-domain fusion and chaotic encryption. The step-by-step process is elaborated as follows: 

Step 1: Domain Transformation (Lines 3–4): The Host MRI Image (*HMI*) and the EPR watermark (*EWI*) are converted from the spatial domain into the DOST domain. This transform provides localised multi-resolution frequency analysis, which is superior for preserving the structural integrity of medical images.

Step 2: Adaptive Factor Calculation (Line 5): The adaptive scaling factors ($\alpha , \beta$) are computed from the LBP features of the host image and the embedding coefficient (*p*).

Step 3: Full-Spectrum Linear Fusion (Line 6): The watermark and host coefficients are linearly fused in the DOST domain: $\widehat{HMI} = \alpha \times DHMI + \beta \times DEWI$. Unlike traditional LSB (Least Significant Bit) methods, this full-spectrum approach leverages all subbands, enabling a high payload capacity.

Step 4: Spatial Reconstruction (Line 7): The Inverse DOST (IDOST) is applied to the fused coefficients ($\widehat{HMI}$) to reconstruct the watermarked image ($HMI^{EWI}$) back into the spatial domain.

Step 5: Multi-Stage Security Layer (Lines 8–9): *Arnold Scrambling*: The watermarked image is subjected to the Arnold Transform to destroy spatial correlations and prevent visual recognition of the host anatomy. *Chaotic Encryption:* A final layer of chaotic encryption is applied to provide pixel-level security, rendering the resulting image ($^E HMI^{EWI}$) statistically indistinguishable from random noise. This ensures that the watermarked data is protected against both visual inspection and statistical cryptanalysis.

Step 6: Secure Parameter Packaging (Lines 11–12): For the non-blind extraction process, the original host coefficients (*DHMI*) are scrambled and encrypted, along with the adaptive scaling factors ($\alpha , \beta$), to form the Secure Key Package ($K_{sec}$). This ensures that only authorised receivers with the correct chaotic keys can retrieve the clinical data.

The major procedures for the above watermark embedding are given in more detail in the subsections below.

### Data acquisition

The MRI images were obtained from The Cancer Imaging Archive (TCIA),^32^ specifically from the TCGA-LGG (Low Grade Glioma) collection (DOI: 10.7937/K9/TCIA.2016.L4LTD3TK). Low-grade glioma is a slow-growing brain tumour that commonly arises from glial cells called astrocytes. The MRI image in the DICOM format is converted to JPG format by the DICOM conversion tools. The processing in JPG is faster, supported by more tools, and allows comparison against most recent works that use JPG format. Another dataset used is the Brain MRI Images for Brain Tumour Detection (BMIBTD) dataset^33^, in which the data is in JPG format. The first step in the algorithm is to read the host MRI image and the watermark image. The watermark image consists of the EPR, which includes the name of the patient, sex, and age. The EPR is embedded in the MRI image for authentication purposes, to maintain patient privacy, and to ensure the integrity of the host MRI image.

### Extraction of local binary pattern features

The next step is to extract the local binary pattern (LBP) features of the host image. LBP creates a texture descriptor that is highly sensitive to the spatial arrangement of pixel values. LBP’s ability to capture fine local textures allows watermarks to be embedded without significantly altering the visible content of the image. This makes LBP a good choice for maintaining the perceptual quality of the image while embedding the watermark.

### Determination of $\alpha$ and $\beta$

After extracting the LBP features, their mean value is calculated to capture the overall texture of the image. This mean value provides a general description of the local texture, rather than focusing on individual pixel-level details. Since LBP features are based on the neighbourhood around each pixel, their mean reflects the dominant texture patterns across a region. Using the mean value ensures a smooth and stable representation, reducing sensitivity to small variations. Embedding the watermark based on this feature helps maintain the image quality, as it minimises visual distortion and keeps the watermark imperceptible to the human eye. The parameter $\beta$ is computed as follows:

8 $$\begin{aligned} \beta =\frac{\mu (LBP_{features}(HMI))}{p} \end{aligned}$$

where p represents the embedding coefficient and $\mu ()$ represents the mean operator. Here, $\alpha$ is determined as $1-\beta$. The parameter p is fixed (more details in the result section, Fig. 5) by a search operation to attain high watermarking metrics.

### Watermark embedding

The proposed method employs a full-spectrum embedding strategy within a 64-bit complex-float computational pipeline, where the watermark’s DOST coefficients are embedded across the entire range of the host MRI image’s subbands. By representing the watermarked image as a 64-bit complex floating-point entity (comprising 32-bit real and 32-bit imaginary components), the framework achieves a high data payload capacity while leveraging the transform’s orthonormality to preserve diagnostic quality at a near-perfect level. Initially, DOST is applied to both the host MRI and the watermark image (line 4 of Algorithm 1), providing a joint space-frequency representation where the dominant structural features of both images are aligned. This spectral alignment ensures that the watermark energy is distributed into the infinitesimal mantissa bits of the host’s frequency characteristics, significantly enhancing imperceptibility. While this high-capacity approach is designed as a semi-fragile scheme—where the high-precision coefficients are intentionally sensitive to the rounding errors inherent in lossy compression and geometric distortions—it remains highly resilient to additive channel noise. The alignment of high-magnitude coefficients (line 6) within this 64-bit complex manifold ensures that under stochastic signal degradation, the watermark can be accurately recovered via the non-blind extraction process. Finally, the inverse DOST (IDOST) is applied (line 7) to reconstruct the watermarked image (a 64-bit per pixel entity), maintaining superior structural similarity to the original host and ensuring a high-fidelity, tamper-evident reference for secure clinical use in closed-loop environments.

## Proposed technique for digital watermarking extraction using discrete orthonormal stockwell transform

**Algorithm 2:**
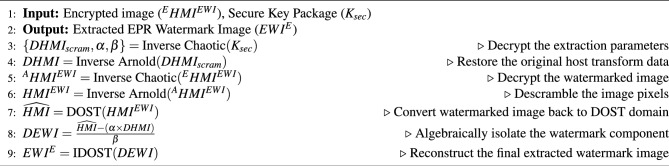
Proposed Secure Watermark Extraction Algorithm

**Fig. 2:**
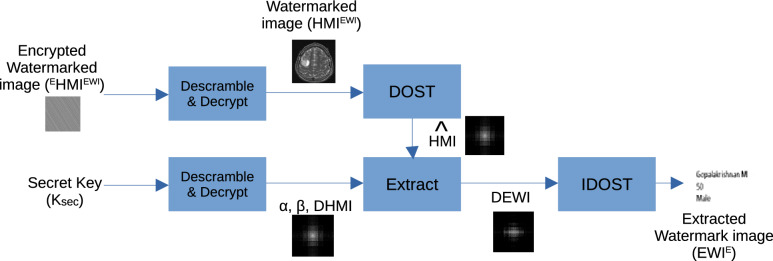
The block diagram of the proposed watermark extraction

The block diagram of the proposed watermark extraction is shown in Fig. 2. The steps for the extraction of the EPR from the embedded images proceed as shown in Algorithm 2.

### Algorithm 2 elaboration: extraction process

The extraction process is a non-blind, symmetric reversal of the embedding stage, requiring the synchronised secure key package ($K_{sec}$) for data recovery. The steps are elaborated as follows: 

Step 1: Parameter Decryption (Lines 3–4): The authorised receiver first decrypts the secure key package using the Inverse Chaotic map to retrieve the adaptive scaling factors ($\alpha , \beta$) and the scrambled host coefficients. The Inverse Arnold transform is then applied to $DHMI_{scram}$ to restore the original host transform data (*DHMI*), which serves as the non-blind reference.

Step 2: Watermarked Image Decryption (Lines 5–6): The received encrypted image ($^{E}HMI^{EWI}$) undergoes a two-stage decryption process. First, the Inverse Chaotic map restores the pixel values, followed by the Inverse Arnold transform to descramble the spatial structure, resulting in the watermarked image $HMI^{EWI}$.

Step 3: Spectral Domain Conversion (Line 7): The watermarked image is transformed back into the DOST domain. This provides the fused coefficients ($\widehat{HMI}$) necessary for algebraic separation.

Step 4: Watermark Isolation (Line 8): Leveraging the non-blind reference (*DHMI*) and the adaptive factors ($\alpha , \beta$), the watermark component (*DEWI*) is algebraically isolated from the host signal. By subtracting the weighted host coefficients from the fused coefficients and normalising by $\beta$, the watermark coefficients are extracted.

Step 5: Final EPR Reconstruction (Line 9): The Inverse DOST (IDOST) is applied to the isolated watermark coefficients (*DEWI*). This reconstructs the Electronic Patient Record ($EWI^E$) into its original spatial format, completing the secure extraction.

### Watermark extraction

The selection of a non-blind, semi-fragile approach is a deliberate architectural choice necessitated by the zero-tolerance requirements for data loss in high-stakes telemedicine. While blind extraction schemes are often preferred for general-purpose watermarking, they frequently suffer from non-zero Bit Error Rates (BER) that are clinically unacceptable for the transmission of sensitive EPR. By leveraging the original host DOST coefficients during the extraction phase, the proposed framework ensures mathematically perfect, 100$\%$ error-free reconstruction of the embedded clinical data—a critical prerequisite for diagnostic reliability. This methodology is specifically designed for secure server-to-server or hospital-to-clinic workflows. Consequently, the watermark functions as a dual-purpose mechanism: serving as a high-capacity carrier for medical metadata while simultaneously acting as a high-sensitivity tamper-detection tool, wherein any unauthorised geometric or lossy modification to the host image triggers a failure in data recovery, alerting the receiver to a compromise in diagnostic integrity.

### Ethical aspects

All methods were carried out in accordance with relevant guidelines and regulations. The original imaging data in the TCGA-LGG collection was acquired under experimental protocols approved by the institutional review boards (IRBs) of the participating institutions as part of The Cancer Genome Atlas (TCGA) program. Informed consent was obtained from all subjects by the original investigators at the time of data collection. This study involved secondary analysis of fully de-identified data obtained from The Cancer Imaging Archive (TCIA); therefore, no additional ethics approval or consent was required for this research.

**Fig. 3:**
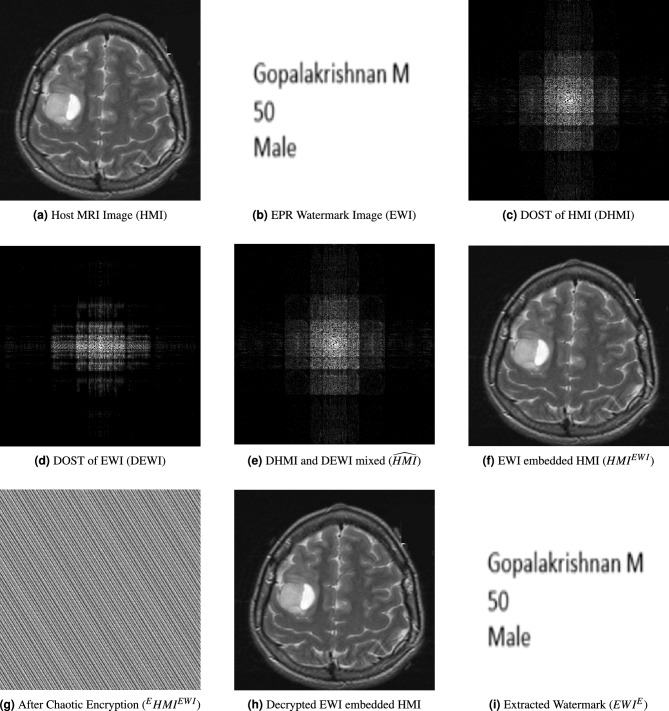
Visual results of the proposed watermarking process across various stages

**Fig. 4:**
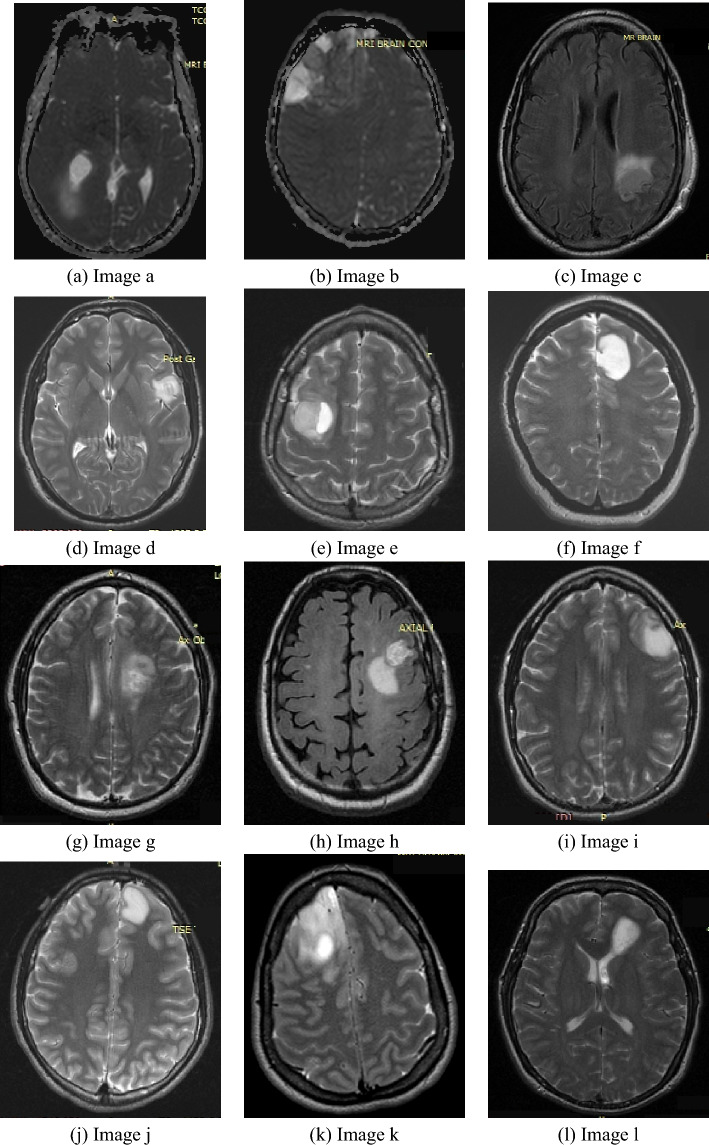
MRI Astrocytoma host images from the TCIA dataset used for watermark embedding

## Experimental results and discussions

The work has been evaluated on MRI brain images from the TCIA dataset^32^ and the BMIBTD dataset^33^. From the TCIA, 12 MRI scans that depicted Astrocytoma tumours were used. The BMIBTD dataset consists of 253 MRI scans of brain tumours (155 images) and without brain tumours (98 images). The visual results of the proposed watermarking process across various stages are shown in Fig. 3. The original MRI image is illustrated in Fig. 3a, while the EPR image utilised as the watermark is shown in Fig. 3b. The Discrete Orthonormal Stockwell Transform of the MRI image and the EPR image is presented in Fig. 3c and Fig. 3d, respectively. The MRI image and EPR image mixed in the transform domain is shown in Fig. 3e. The watermarked image is seen in Fig. 3f. The watermarked image after scrambling and encryption is shown in Fig. 3g. The decrypted and descrambled watermarked image at the receiver end is seen in Fig. 3h. The extracted watermark from the watermarked image is depicted in Fig. 3i. All implementation and experimentation were carried out using MATLAB R2020b. Fig. 4 shows typical MRI host images used to evaluate the performance of the proposed system.

### Performance evaluation metrics

The proposed system is evaluated using the following metrics:

#### 1. Peak signal-to-noise ratio (PSNR)

The PSNR evaluates the difference between the original host image and the watermarked image.

9 $$\begin{aligned} PSNR = 10 \cdot \log _{10} \left( \frac{MAX_I^2}{MSE} \right) \end{aligned}$$

where *MSE* (Mean Squared Error) is defined as:

10 $$\begin{aligned} MSE = \frac{1}{M \times N} \sum _{i=0}^{M-1} \sum _{j=0}^{N-1} [HMI(i,j) - HMI^{EWI}(i,j)]^2 \end{aligned}$$

- *M*, *N*: Height and width of the image.
- *HMI*(*i*, *j*): Pixel value of the original host image at coordinates (*i*, *j*).
- $HMI^{EWI}(i,j)$: Pixel value of the watermarked image at coordinates (*i*, *j*).
- $MAX_I$: Maximum possible pixel value of the image (255 for 8-bit images).

#### 2. Structural similarity index (SSIM)

SSIM measures the degradation of structural information between the original host and the watermarked images.

11 $$\begin{aligned} SSIM(HMI, HMI^{EWI}) = \frac{(2\mu _{HMI}\mu _{HMI^{EWI}} + c_1)(2\sigma _{HMI, HMI^{EWI}} + c_2)}{(\mu _{HMI}^2 + \mu _{HMI^{EWI}}^2 + c_1)(\sigma _{HMI}^2 + \sigma _{HMI^{EWI}}^2 + c_2)} \end{aligned}$$

- $\mu _{HMI}, \mu _{HMI^{EWI}}$: The pixel sample Gaussian-weighted averages of *HMI* and $HMI^{EWI}$.
- $\sigma _{HMI}^2, \sigma _{HMI^{EWI}}^2$: The variance of *HMI* and $HMI^{EWI}$.
- $\sigma _{HMI, HMI^{EWI}}$: The covariance of *HMI* and $HMI^{EWI}$.
- $c_1, c_2$: Constants used to stabilise the division.

#### 3. Kullback-Leibler Divergence (KLD)

KLD quantifies how the probability distribution of the watermarked image differs from the original host image.

12 $$\begin{aligned} KLD = D_{KL}(P_{HMI} \parallel P_{HMI^{EWI}}) + D_{KL}(P_{HMI^{EWI}} \parallel P_{HMI}) \end{aligned}$$

where:

13 $$\begin{aligned} D_{KL}(P \parallel Q) = \sum _{i} P(i) \log \frac{P(i)}{Q(i)} \end{aligned}$$

- $P_{HMI}(i)$: Probability distribution of the host medical image pixels.
- $P_{HMI^{EWI}}(i)$: Probability distribution of the watermarked image pixels.

#### 4. Jensen-Shannon distance (JSD)

The Jensen-Shannon distance is another statistical measure of how much the host image changes after the watermark is embedded.

14 $$\begin{aligned} JSD(P_{HMI} \parallel P_{HMI^{EWI}}) = \sqrt{JSDiv(P_{HMI} \parallel P_{HMI^{EWI}})} \end{aligned}$$

where the divergence *JSDiv* is defined as:

15 $$\begin{aligned} JSDiv(P_{HMI} \parallel P_{HMI^{EWI}}) = \frac{1}{2} D_{KL}(P_{HMI} \parallel \mathcal {M}) + \frac{1}{2} D_{KL}(P_{HMI^{EWI}} \parallel \mathcal {M}) \end{aligned}$$

- $\mathcal {M}$: The average distribution, calculated as $\mathcal {M} = \frac{1}{2}(P_{HMI} + P_{HMI^{EWI}})$.
- $D_{KL}$: The Kullback-Leibler Divergence as defined previously.

#### 5. Normalised correlation coefficient (NCC)

NCC is used to judge the robustness by comparing the original and extracted watermarks.

16 $$\begin{aligned} NCC = \frac{\sum _{i=1}^{n} (EWI_i - \overline{EWI}) \cdot (EWI^E_i - \overline{EWI^E})}{\sqrt{\sum _{i=1}^{n} (EWI_i - \overline{EWI})^2} \cdot \sqrt{\sum _{i=1}^{n} (EWI^E_i - \overline{EWI^E})^2}} \end{aligned}$$

- $EWI_i$: Pixels of the original watermark.
- $EWI^E_i$: Pixels of the extracted watermark.
- $\overline{EWI}, \overline{EWI^E}$: The mean pixel values of the original and extracted watermarks, respectively.
- *n*: Total number of elements in the watermark.

#### 6. Bit error rate (BER)

BER measures the accuracy of the watermark extraction process.

17 $$\begin{aligned} BER = \frac{B_e}{B_t} \end{aligned}$$

- $B_e$: Number of bits in $EWI^E$ incorrectly extracted compared to *EWI*.
- $B_t$: Total number of watermark bits embedded.

#### 7. Number of pixels change rate (NPCR)

18 $$\begin{aligned} NPCR = \frac{\sum _{i,j} \mathcal {D}(i,j)}{M \times N} \times 100\% \end{aligned}$$

- $\mathcal {D}(i,j)$: Binary function where $\mathcal {D}(i,j) = 1$ if $I_1(i,j) \ne I_2(i,j)$, and 0 otherwise.
- $I_1, I_2$: Two images whose metric is to be calculated.
- *M*, *N*: Height and width of the images.

#### 8. Unified average changing intensity (UACI)

19 $$\begin{aligned} UACI = \frac{1}{M \times N} \left[ \sum _{i,j} \frac{|I_1(i,j) - I_2(i,j)|}{255} \right] \times 100\% \end{aligned}$$

The imperceptibility of the watermark in the host image is studied by finding the PSNR, SSIM^34^, KLD, JSD, $\text {NPCR}_H$, and $\text {UACI}_H$ metrics between the host image and the watermarked host image. The robustness of the proposed system in recovering the watermark is studied by finding the NCC, BER, $\text {NPCR}_W$, and $\text {UACI}_W$ metrics between the original watermark image and the extracted watermark image.

**Fig. 5:**
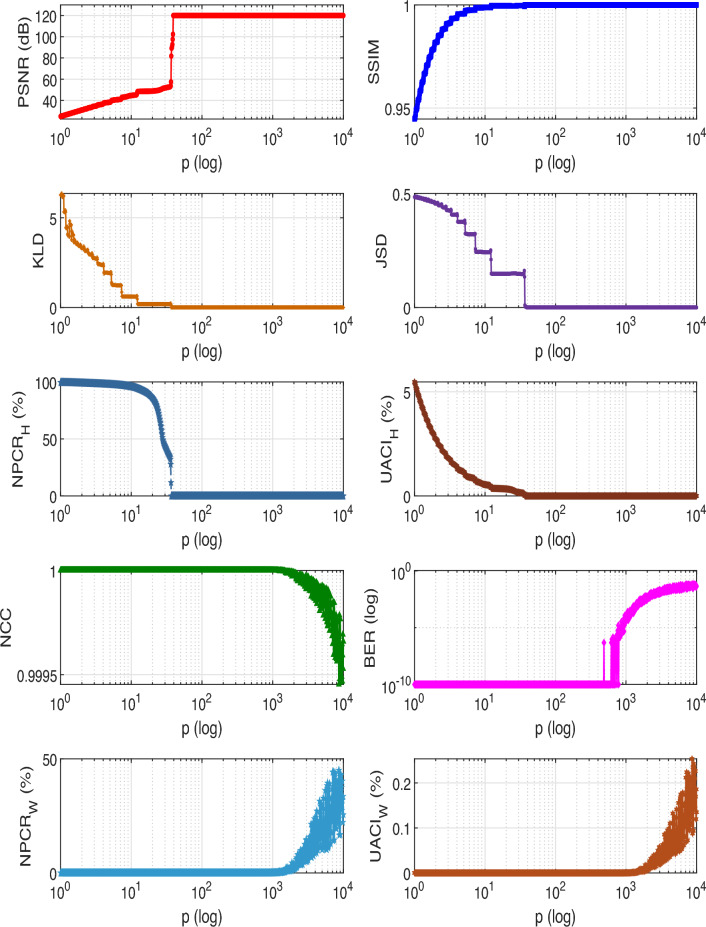
The impact of embedding coefficient (p) on PSNR, SSIM, KLD, JSD, $\text {NPCR}_H$, $\text {UACI}_H$, NCC, BER, $\text {NPCR}_W$, $\text {UACI}_W$ in the proposed method. (For visualisation purposes, infinite PSNR values are capped at 120 dB, while zero BER values are floored at $1\times 10^{-10}$)

### Analysis of the impact of embedding coefficient on performance

The performance of the proposed method is sensitive to the embedding coefficient (p). The impact of p on PSNR, SSIM, KLD, JSD, $\text {NPCR}_H$, $\text {UACI}_H$, NCC, BER, $\text {NPCR}_W$, and $\text {UACI}_W$ is presented in Fig. 5. As p is increased, the PSNR will increase as the embedding factor ($\beta$) will reduce, thereby reducing the amount of watermark image embedded. Beyond a certain value of p, the PSNR value (see Eqn. 9) approaches infinity as the mean square error between the host image and the watermarked image nears zero. This behaviour is because the watermark is embedded as coefficients in the lower significant positions of the mantissa of the host image’s pixel values, which are represented as floating point. This embedding ensures that the host image’s visual information is not severely impacted after watermarking, which is a critical requirement in medical image watermarking. When the watermarked image is transmitted to the receiver end as a floating point representation, more bytes need to be sent, but this is mitigated by the greater capacity of the proposed method. This is discussed later in the capacity analysis subsection (see Table 9). The SSIM value plateaus to a value of 1 as the p value increases, as watermark embedding is reduced. The KLD, JSD, $\text {NPCR}_H$, and $\text {UACI}_H$ values decrease to zero as the p value is increased. The NCC value, meanwhile, reduces from a plateau of 1 as the p value increases, as the watermark embedding reduces. The BER, $\text {NPCR}_W$, and $\text {UACI}_W$ values increase for high values of p because of the reduced watermark embedding. As the p value has an impact on performance in the proposed method, the p value is fixed in the following analysis by performing a search to find the value of p that gives a PSNR value just below infinity and that gives high SSIM and NCC values.

### Imperceptibility analysis and general robustness analysis of the proposed system

**Table 2:** Comparison of Imperceptibility Metrics (Host vs. Watermarked Image) on the TCIA dataset. (Values: Mean / Std., PSNR in dB, NPCR and UACI in $\%$.).

Img	Proposed method						Vaidya’s method^4^						Chaudhary’s method^5^					
	PSNR	SSIM	KLD	JSD	$\text {NPCR}_H$	$\text {UACI}_H$	PSNR	SSIM	KLD	JSD	$\text {NPCR}_H$	$\text {UACI}_H$	PSNR	SSIM	KLD	JSD	$\text {NPCR}_H$	$\text {UACI}_H$
a	102.31	1.0000	$1\times 10^{-7}$	$1\times 10^{-4}$	$4\times 10^{-4}$	$1\times 10^{-6}$	42.40	0.9937	1.910	0.382	84.75	0.608	49.79	0.9998	0.027	0.057	68.21	0.268
b	67.20	0.9986	0.130	0.088	1.24	0.005	44.02	0.9896	3.514	0.498	66.31	0.479	50.68	0.9996	0.036	0.065	55.53	0.218
c	65.10	0.9977	0.119	0.091	2.01	0.008	37.51	0.8891	2.240	0.406	87.15	1.068	50.31	0.9993	0.009	0.033	60.34	0.237
d	84.91	1.0000	0.003	0.010	0.02	$8\times 10^{-5}$	35.99	0.9974	0.535	0.235	95.07	1.293	49.94	0.9999	0.005	0.025	65.88	0.259
e	102.32	1.0000	$4\times 10^{-5}$	0.001	$4\times 10^{-4}$	$1.5\times 10^{-6}$	37.12	0.9959	1.158	0.320	95.34	1.169	50.60	0.9999	0.004	0.021	56.69	0.222
f	94.53	1.0000	$9\times 10^{-5}$	0.002	$2\times 10^{-3}$	$9\times 10^{-6}$	36.33	0.9978	0.333	0.164	93.66	1.106	50.12	0.9998	0.006	0.028	63.23	0.248
g	71.64	0.9999	0.020	0.040	0.45	0.002	35.83	0.9473	0.802	0.262	87.03	1.305	50.16	0.9998	0.005	0.023	62.55	0.245
h	72.37	0.9999	0.009	0.031	0.38	0.002	35.27	0.9370	1.105	0.310	96.61	1.504	50.97	0.9998	0.004	0.021	51.94	0.204
i	91.17	1.0000	$7\times 10^{-4}$	0.005	$5\times 10^{-3}$	$2\times 10^{-5}$	37.61	0.9911	0.939	0.300	87.20	1.069	50.09	0.9999	0.006	0.027	63.62	0.250
j	84.25	1.0000	0.004	0.011	0.02	$1\times 10^{-4}$	34.83	0.9947	0.934	0.290	97.24	1.538	50.39	0.9999	0.004	0.022	59.41	0.233
k	53.67	0.9664	2.169	0.414	27.90	0.109	38.13	0.8109	3.450	0.445	86.24	1.005	51.02	0.9995	0.006	0.026	48.99	0.195
l	68.53	0.9998	0.036	0.055	0.91	0.004	37.74	0.9198	1.325	0.312	83.41	1.007	50.46	0.9999	0.010	0.033	58.44	0.230
Mean	79.84	0.9969	0.208	0.063	2.74	0.011	37.73	0.9553	1.520	0.327	88.34	1.095	50.38	0.9997	0.010	0.032	59.57	0.234
Std.	15.70	0.0096	0.620	0.116	7.95	0.031	2.78	0.0585	1.057	0.092	8.54	0.314	0.39	$2\times 10^{-4}$	0.010	0.015	5.64	0.022

**Table 3:** Comparison of general robustness metrics (original vs. extracted watermark) on the TCIA dataset. (NPCR and UACI in $\%$.)

Img	Proposed method					Vaidya’s method^4^				Chaudhary’s method^5^			
	*p*	NCC	BER	$\text {NPCR}_W$	$\text {UACI}_W$	NCC	BER	$\text {NPCR}_W$	$\text {UACI}_W$	NCC	BER	$\text {NPCR}_W$	$\text {UACI}_W$
a	34.72	1.0000	0	0	0	1.0000	0	0	0	0.9760	0.053	12.26	1.959
b	25.02	1.0000	0	0	0	1.0000	0	0	0	0.9831	0.045	11.60	0.677
c	44.92	1.0000	0	0	0	1.0000	0	0	0	0.9860	0.056	12.55	2.681
d	41.07	1.0000	0	0	0	1.0000	0	0	0	0.9740	0.054	12.43	1.607
e	38.90	1.0000	0	0	0	1.0000	0	0	0	0.9854	0.048	11.79	1.029
f	32.21	1.0000	0	0	0	1.0000	0	0	0	0.9653	0.059	14.48	1.476
g	43.58	1.0000	0	0	0	1.0000	0	0	0	0.9571	0.045	11.77	0.762
h	39.90	1.0000	0	0	0	1.0000	0	0	0	0.9874	0.062	15.23	2.154
i	40.90	1.0000	0	0	0	1.0000	0	0	0	0.9691	0.048	11.64	1.053
j	40.40	1.0000	0	0	0	1.0000	0	0	0	0.9593	0.047	11.72	0.997
k	30.70	1.0000	0	0	0	1.0000	0	0	0	0.9801	0.052	16.72	0.570
l	38.56	1.0000	0	0	0	1.0000	0	0	0	0.9812	0.051	12.23	1.276
Mean	37.57	1.0000	0	0	0	1.0000	0	0	0	0.9754	0.052	12.87	1.353
Std.	5.81	0	0	0	0	0	0	0	0	0.0105	0.006	1.68	0.647

**Table 4:** Comparison of Imperceptibility Metrics (Host vs. Watermarked Image) on the BMIBTD dataset. (Values: Mean / Std., PSNR in dB, NPCR and UACI in $\%$, Data Used - Full = entire data, BT = only Brain Tumour data, BTF = only Brain Tumour Free data.)

Data	Method	PSNR	SSIM	KLD	JSD	$\text {NPCR}_H$	$\text {UACI}_H$
Full	Vaidya’s^4^	36.96 / 3.12	0.8842 / 0.0814	3.076 / 1.919	0.409 / 0.105	91.56 / 6.84	1.265 / 0.469
	Chaudhary’s^5^	50.83 / 0.95	0.9992 / 0.0012	0.015 / 0.022	0.037 / 0.020	53.73 / 10.41	0.212 / 0.039
	Proposed	65.62 / 15.45	0.9827 / 0.0205	1.024 / 1.189	0.209 / 0.184	15.59 / 17.34	0.061 / 0.068
BT	Vaidya’s^4^	35.85 / 2.79	0.8902 / 0.0789	2.809 / 1.833	0.380 / 0.100	91.96 / 6.81	1.397 / 0.477
	Chaudhary’s^5^	50.60 / 0.67	0.9993 / 0.0010	0.015 / 0.019	0.037 / 0.019	56.44 / 9.60	0.0223 / 0.036
	Proposed	67.18 / 16.03	0.9861 / 0.0173	0.945 / 1.167	0.188 / 0.176	12.83 / 14.99	0.050 / 0.059
BTF	Vaidya’s^4^	38.71 / 2.81	0.8747 / 0.0848	3.498 / 1.985	0.455 / 0.096	90.92 / 6.87	1.055 / 0.370
	Chaudhary’s^5^	51.19 / 1.19	0.9990 / 0.0013	0.016 / 0.026	0.037 / 0.022	49.44 / 10.23	0.195 / 0.039
	Proposed	63.15 / 14.22	0.9773 / 0.0239	1.149 / 1.219	0.242 / 0.193	19.94 / 19.82	0.078 / 0.077

**Table 5:** Comparison of general robustness metrics (original vs. extracted watermark) on the BMIBTD dataset. (Values: Mean / Std., NPCR and UACI in $\%$, Data Used - Full = entire data, BT = only Brain Tumour data, BTF = only Brain Tumour Free data.)

Data	Method	*p*	NCC	BER	$\text {NPCR}_W$	$\text {UACI}_W$
Full	Vaidya’s^4^	–	1.0000 / 0	0 / 0	0 / 0	0 / 0
	Chaudhary’s^5^	–	0.9087 / 0.1704	0.087 / 0.078	24.12 / 20.81	2.690 / 6.332
	Proposed	33.15 / 8.84	1.0000 / 0	0 / 0	0 / 0	0 / 0
BT	Vaidya’s^4^	–	1.0000 / 0	0 / 0	0 / 0	0 / 0
	Chaudhary’s^5^	–	0.9191 / 0.1468	0.080 / 0.068	21.72 / 18.31	2.513 / 4.194
BTF	Proposed	35.93 / 7.54	1.0000 / 0	0 / 0	0 / 0	0 / 0
	Vaidya’s^4^	–	1.0000 / 0	0 / 0	0 / 0	0 / 0
	Chaudhary’s^5^	–	0.8923 / 0.2020	0.096 / 0.090	27.90 / 23.86	2.972 / 8.705
	Proposed	28.76 / 9.00	1.0000 / 0	0 / 0	0 / 0	0 / 0

Tables 2 and 4 show the imperceptibility analysis metrics of the proposed method using different images and its comparison with current SOTA techniques (Vaidya’s Method^4^ and Chaudhary’s Method^5^) on the TCIA dataset and the BMIBTD dataset, respectively. The general robustness analysis metrics and embedding coefficient (*p*) value for these two datasets are given in Tables 3 and 5, respectively. It is found that the proposed method has better imperceptibility metrics than Vaidya’s method but slightly less than Chaudhary’s method (for Chaudhary’s method, all imperceptibility metrics are better except $\text {NPCR}_H$ and $\text {UACI}_H$). But the robustness metrics of the extracted watermark are poor for Chaudhary’s method when compared to the proposed method and Vaidya’s method, both of which promise perfect extraction of the watermark.

On looking closer at the proposed method, even after embedding, almost all the images have a PSNR greater than 50 dB, which indicates that the embedded EPR has not affected the content or quality of the host images. Medical images are used for diagnosis and treatment planning, and any distortion or error in the image can lead to misdiagnosis or incorrect treatment. A high PSNR value ensures that the watermarked image is of high quality and preserves the diagnostic integrity of the original image. Moreover, a high value of SSIM indicates that the watermark embedded image preserves the structural information of the original host image. This is particularly important in medical imaging, where accurate diagnosis relies on the ability to detect subtle changes in image structure and texture. The KLD, JSD, $\text {NPCR}_H$, and $\text {UACI}_H$ are also low, indicating little visual change between the host image and the watermarked image. The NCC between the original watermark image and the extracted watermark image is 1 in all cases. A high NCC value indicates that the extracted watermark is highly similar to the original, which ensures that the EPR data is retrieved correctly without any distortions. The BER, $\text {NPCR}_W$, and $\text {UACI}_W$ are all zeros, indicating perfect extraction of the watermark.

**Table 6:** Comparison of robustness metrics on original vs. extracted watermark under various non-geometric attacks on the TCIA dataset (Values: Mean / Std.). The subscript $_{ae}$ indicates the attack after scrambling and encryption, while the subscript $_{be}$ indicates the attack before scrambling and encryption. Chaudhary’s method does not scramble or encrypt the watermarked image

Attack type	Method	NCC	BER	$\text {NPCR}_W$	$\text {UACI}_W$
Attack free	Vaidya^4^	1.0000 / 0	0 / 0	0 / 0	0 / 0
	Chaudhary^5^	0.9754 / 0.0105	0.052 / 0.006	12.87 / 1.68	1.353 / 0.647
	Proposed	1.0000 / 0	0 / 0	0 / 0	0 / 0
Gaussian noise ($\mu$ = 0, $\sigma ^2$ = 0.002)	Vaidya$_{ae}$	0.9997 / 0.0002	0.051 / 0.007	33.60 / 3.85	0.166 / 0.044
	Vaidya$_{be}$	0.9997 / 0.0002	0.051 / 0.008	33.51 / 3.90	0.166 / 0.044
	Chaudhary	0.9754 / 0.0105	0.052 / 0.006	12.87 / 1.68	1.353 / 0.647
	Proposed$_{ae}$	0.8841 / 0.0086	0.173 / 0.002	54.23 / 0.09	3.575 / 0.153
	Proposed$_{be}$	0.8838 / 0.0085	0.174 / 0.002	54.25 / 0.10	3.577 / 0.152
Gaussian noise ($\mu$ = 0.01, $\sigma ^2$ = 0.002)	Vaidya$_{ae}$	0.9998 / 0.0001	0.033 / 0.005	20.00 / 2.66	0.097 / 0.026
	Vaidya$_{be}$	0.9998 / 0.0001	0.033 / 0.005	20.16 / 2.72	0.097 / 0.026
	Chaudhary	0.9754 / 0.0105	0.052 / 0.006	12.87 / 1.68	1.353 / 0.647
	Proposed$_{ae}$	0.8960 / 0.0081	0.146 / 0.002	46.34 / 0.08	2.848 / 0.122
	Proposed$_{be}$	0.8958 / 0.0080	0.146 / 0.002	46.36 / 0.09	2.850 / 0.121
Salt and pepper (noise density = 0.001)	Vaidya$_{ae}$	0.7137 / 0.0182	0.025 / 0.003	6.20 / 0.69	1.735 / 0.182
	Vaidya$_{be}$	0.7028 / 0.0241	0.026 / 0.003	6.24 / 0.61	1.782 / 0.190
	Chaudhary	0.8665 / 0.0660	0.081 / 0.021	20.90 / 5.84	3.344 / 1.016
	Proposed$_{ae}$	0.9995 / 0.0001	$4\times 10^{-5}$ / $9\times 10^{-6}$	0.01 / $2\times 10^{-3}$	0.003 / $6\times 10^{-4}$
	Proposed$_{be}$	0.9989 / 0.0017	$5\times 10^{-5}$ / $4\times 10^{-5}$	0.01 / $4\times 10^{-3}$	0.004 / 0.004
Salt and pepper (noise density = 0.002)	Vaidya$_{ae}$	0.5651 / 0.0231	0.048 / 0.005	11.51 / 1.21	3.311 / 0.345
	Vaidya$_{be}$	0.5601 / 0.0256	0.048 / 0.004	11.58 / 1.02	3.350 / 0.346
	Chaudhary	0.7152 / 0.1178	0.109 / 0.030	26.48 / 6.82	6.152 / 2.233
	Proposed$_{ae}$	0.9990 / $2\times 10^{-4}$	$8\times 10^{-5}$ / $1\times 10^{-5}$	0.02 / $3\times 10^{-3}$	0.005 / 0.001
	Proposed$_{be}$	0.9978 / 0.0034	$1\times 10^{-4}$ / $8\times 10^{-5}$	0.02 / $8\times 10^{-3}$	0.008 / 0.008
JPEG compression (quality = 80 $\%$)	Vaidya$_{ae}$	0.1376 / 0.0410	0.256 / 0.010	53.99 / 2.16	22.032 / 1.587
	Vaidya$_{be}$	0.7320 / 0.0786	0.202 / 0.035	56.71 / 6.36	6.028 / 1.587
	Chaudhary	0.9736 / 0.0174	0.058 / 0.010	14.22 / 4.28	2.029 / 0.752
	Proposed$_{ae}$	0 / 0	0.042 / 0	10.48 / 0	2.773 / $1\times 10^{-15}$
	Proposed$_{be}$	-0.0441 / 0.0322	0.266 / 0.045	47.38 / 7.77	32.678 / 8.033
Median filter (3$\times$3 window)	Vaidya$_{ae}$	0.0878 / 0.0294	0.138 / 0.017	31.33 / 3.24	10.664 / 1.706
	Vaidya$_{be}$	0.6741 / 0.0629	0.166 / 0.013	51.67 / 4.97	4.778 / 0.434
	Chaudhary	-0.1888 / 0.0255	0.295 / 0.034	60.65 / 4.10	23.166 / 5.037
	Proposed$_{ae}$	0.0842 / 0.0044	0.042 / $2\times 10^{-5}$	10.38 / $5\times 10^{-3}$	2.752 / 0.002
	Proposed$_{be}$	0.8021 / 0.0863	0.036 / 0.008	13.68 / 2.20	1.271 / 0.777
Median filter (5$\times$5 window)	Vaidya$_{ae}$	0.0530 / 0.0284	0.124 / 0.020	26.78 / 3.94	10.578 / 2.032
	Vaidya$_{be}$	0.3474 / 0.0296	0.205 / 0.020	52.28 / 5.29	10.931 / 1.335
	Chaudhary	-0.3097 / 0.0313	0.527 / 0.043	73.41 / 4.09	51.019 / 4.585
	Proposed$_{ae}$	0.0267 / 0.0053	0.042 / $1\times 10^{-5}$	10.47 / $3\times 10^{-3}$	2.774 / $2\times 10^{-4}$
	Proposed$_{be}$	0.5597 / 0.1192	0.054 / 0.012	20.42 / 2.24	2.366 / 1.217
Mean filter (3$\times$3 window)	Vaidya$_{ae}$	0.0245 / 0.0255	0.105 / 0.016	22.68 / 2.97	8.970 / 1.597
	Vaidya$_{be}$	0.4966 / 0.0440	0.176 / 0.011	50.35 / 3.29	6.926 / 0.709
	Chaudhary	-0.2250 / 0.0152	0.375 / 0.029	61.30 / 2.83	34.495 / 3.479
	Proposed$_{ae}$	$-7\times 10^{-4}$ / $2\times 10^{-4}$	0.042 / $2\times 10^{-6}$	10.48 / $5\times 10^{-4}$	2.774 / $2\times 10^{-4}$
	Proposed$_{be}$	0.0415 / 0.0495	0.173 / 0.022	36.03 / 3.49	16.718 / 1.941
Mean filter (5$\times$5 window)	Vaidya$_{ae}$	0.0220 / 0.0423	0.104 / 0.022	22.32 / 4.23	8.826 / 2.271
	Vaidya$_{be}$	0.2685 / 0.0360	0.199 / 0.017	49.10 / 3.76	11.869 / 1.631
	Chaudhary	-0.2887 / 0.0122	0.515 / 0.019	66.49 / 1.96	50.023 / 1.905
	Proposed$_{ae}$	$-4\times 10^{-4}$ / $2\times 10^{-4}$	0.042 / $2\times 10^{-6}$	10.48 / $3\times 10^{-4}$	2.774 / $2\times 10^{-4}$
	Proposed$_{be}$	0.0039 / 0.0411	0.138 / 0.019	28.83 / 3.71	12.280 / 1.995
Histogram equalisation	Vaidya$_{ae}$	-0.0184 / 0.0397	0.058 / 0.010	13.74 / 2.11	4.250 / 1.041
	Vaidya$_{be}$	-0.0184 / 0.0397	0.058 / 0.010	13.74 / 2.11	4.250 / 1.041
	Chaudhary	0.6114 / 0.2772	0.049 / 0.002	11.72 / 0	3.333 / 0.694
	Proposed$_{ae}$	0.0055 / 0.0056	0.043 / $3\times 10^{-4}$	10.68 / 0.08	2.835 / 0.026
	Proposed$_{be}$	-0.0050 / 0.0101	0.043 / 0.002	10.73 / 0.42	2.894 / 0.221

**Table 7:** Comparison of Robustness Metrics on Original vs. Extracted Watermark Under Various Geometric Attacks on the TCIA dataset (Values: Mean / Std.). The subscript $_{ae}$ indicates the attack after scrambling and encryption, while the subscript $_{be}$ indicates the attack before scrambling and encryption. Chaudhary’s method does not scramble or encrypt the watermarked image

Attack type	Method	NCC	BER	$\text {NPCR}_W$	$\text {UACI}_W$
Attack free	Vaidya^4^	1.0000 / 0	0 / 0	0 / 0	0 / 0
	Chaudhary^5^	0.9754 / 0.0105	0.052 / 0.006	12.87 / 1.68	1.353 / 0.647
	Proposed	1.0000 / 0	0 / 0	0 / 0	0 / 0
Scaling (2$\times$ followed by 0.5$\times$)	Vaidya$_{ae}$	0.0119 / 0.0288	0.181 / 0.017	37.21 / 3.33	16.716 / 1.744
	Vaidya$_{be}$	0.9829 / 0.0216	0.083 / 0.006	43.55 / 3.33	0.589 / 0.211
	Chaudhary	0.9459 / 0.0315	0.052 / 0.006	12.97 / 1.61	1.297 / 0.343
	Proposed$_{ae}$	$9\times 10^{-6}$ / 0.0013	0.042 / $6\times 10^{-6}$	10.49 / $2\times 10^{-3}$	2.775 / $4\times 10^{-4}$
	Proposed$_{be}$	0.3557 / 0.0594	0.214 / 0.025	50.95 / 5.49	12.667 / 2.165
Rotation ($\theta$ = 1${^{\circ }}$)	Vaidya$_{ae}$	0.0230 / 0.0199	0.084 / 0.014	18.67 / 2.59	6.854 / 1.371
	Vaidya$_{be}$	0.1116 / 0.0375	0.211 / 0.025	46.54 / 5.13	16.661 / 2.354
	Chaudhary	0.1569 / 0.1601	0.271 / 0.046	51.71 / 5.82	23.174 / 5.442
	Proposed$_{ae}$	-0.0002 / 0.0013	0.042 / $3\times 10^{-6}$	10.48 / $7\times 10^{-4}$	2.774 / $4\times 10^{-4}$
	Proposed$_{be}$	0.0062 / 0.0327	0.090 / 0.012	20.73 / 2.61	7.033 / 0.990
Rotation ($\theta$ = 2${^{\circ }}$)	Vaidya$_{ae}$	0.0237 / 0.0163	0.088 / 0.018	19.45 / 3.36	7.274 / 1.766
	Vaidya$_{be}$	0.0554 / 0.0343	0.195 / 0.025	42.58 / 5.16	16.061 / 2.324
	Chaudhary	0.1286 / 0.1096	0.337 / 0.053	55.24 / 5.65	31.714 / 6.260
	Proposed$_{ae}$	$1\times 10^{-5}$ / 0.0020	0.042 / $3\times 10^{-6}$	10.48 / $6\times 10^{-4}$	2.774 / $3\times 10^{-4}$
	Proposed$_{be}$	-0.0084 / 0.0272	0.080 / 0.010	18.45 / 2.20	6.111 / 0.821
Rotation ($\theta$ = 5${^{\circ }}$)	Vaidya$_{ae}$	0.0193 / 0.0182	0.083 / 0.012	18.33 / 2.33	6.687 / 1.230
	Vaidya$_{be}$	0.0127 / 0.0278	0.176 / 0.025	38.22 / 5.23	14.675 / 2.340
	Chaudhary	0.1712 / 0.0737	0.415 / 0.080	59.26 / 7.42	41.215 / 8.976
	Proposed$_{ae}$	0.0017 / 0.0042	0.042 / $2\times 10^{-6}$	10.48 / $5\times 10^{-4}$	2.774 / $2\times 10^{-4}$
	Proposed$_{be}$	-0.0177 / 0.0198	0.072 / 0.009	16.77 / 1.69	5.452 / 0.772
Rotation ($\theta$ = 90${^{\circ }}$)	Vaidya$_{ae}$	0.0282 / 0.0135	0.086 / 0.015	18.99 / 2.77	7.080 / 1.482
	Vaidya$_{be}$	-0.0022 / 0.0293	0.138 / 0.023	30.71 / 4.18	11.250 / 2.381
	Chaudhary	0.9754 / 0.0105	0.052 / 0.006	12.87 / 1.68	1.353 / 0.647
	Proposed$_{ae}$	$5\times 10^{-5}$ / 0.0020	0.042 / $6\times 10^{-6}$	10.48 / $1\times 10^{-3}$	2.774 / $4\times 10^{-4}$
	Proposed$_{be}$	-0.0018 / 0.0163	0.049 / 0.003	14.19 / 1.28	2.942 / 0.232

**Fig. 6:**
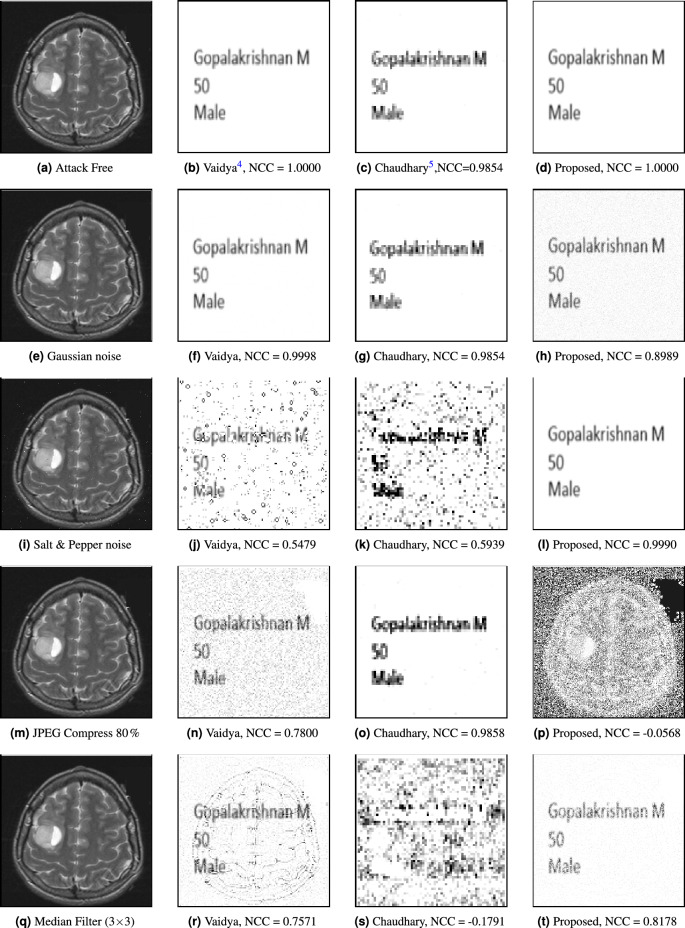
Visual comparison of extracted watermarks using the TCIA dataset. First column represents the watermarked host images after the respective attack. Gaussian noise parameters are $\mu$ = 0.01, $\sigma ^2$ = 0.002, Salt and Pepper noise parameter is noise density = 0.002. Refer to the caption of Fig. 7 for technical details regarding watermark resolutions

**Fig. 7:**
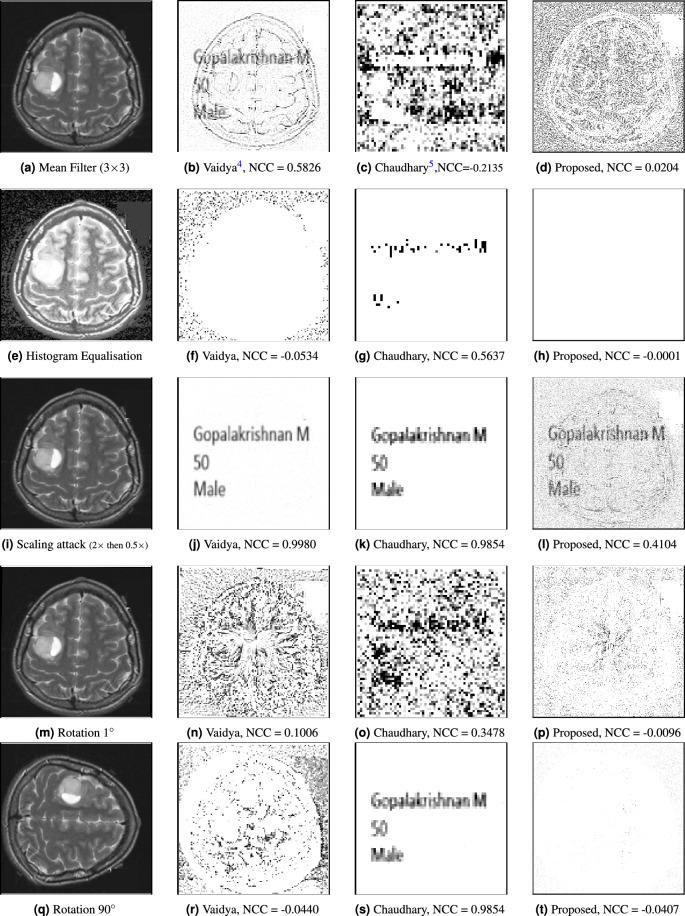
Visual comparison of extracted watermarks using the TCIA dataset. First column represents the watermarked host images after the respective attack. For the proposed method, the extraction utilises the native 512$\times$512 watermark resolution, while Vaidya’s^4^ and Chaudhary’s^5^ extractions correspond to their native 128$\times$128 and 64$\times$64 dimensions, respectively. NCC values are calculated on these native dimensions without interpolation to prevent artificial smoothing bias

### Attack robustness analysis of the proposed system

In order to evaluate the robustness of the watermarking method, the EPR embedded brain tumour MRI images are subjected to different types of non-geometric and geometric attacks. The non-geometric attacks performed include Gaussian noise, Salt & Pepper noise, JPEG Compression, Median Filter, Mean Filter, and Histogram Equalisation. The geometric attacks performed include Scaling and various Rotation attacks. The robustness of the proposed system is studied using NCC, BER, $\text {NPCR}_W$, and $\text {UACI}_W$ metrics calculated between the original watermark image and the extracted watermark image. The results of various attacks on the proposed method are presented in Tables 6 and 7 and compared against other SOTA methods. These tables present results for attacks performed before and after encryption in methods that include encryption of the watermarked image. The attacks performed after encryption usually lead to poor watermark extraction robustness. The visual comparison of the extracted watermarks is seen in Fig. 6 and Fig. 7. These figures present the result of watermark extraction when attacks are performed before encryption.

#### Robustness analysis: Gaussian noise attack

As evidenced by the results in Table 6, the proposed method demonstrates comparable resilience to Gaussian noise attacks ($\mu$ = 0, $\sigma ^2$ = 0.002 and $\mu$ = 0.01, $\sigma ^2$ = 0.002) compared to recent SOTA works. The visual recovery of the watermark is compared in Fig. 6e-h. While Gaussian noise typically introduces random variations that affect fine structural details and high-frequency textures, the proposed method leverages the inherent spectral energy concentration of the DOST.

In the DOST domain, Gaussian noise manifests as a stochastic distribution across the 64-bit complex-float coefficient plane. Because the MRI’s structural energy—representing brain tissue boundaries and tumour morphology—is concentrated in low-frequency subbands, these regions maintain a high Signal-to-Noise Ratio (SNR) that effectively shields the embedded bits. In the high-frequency subbands, where coefficient magnitudes are naturally lower, the non-blind extraction framework utilises the original host’s complex-float DOST map to decouple the watermark signal from the structural interference. While stochastic noise fluctuations persist in the recovered residual, this isolation ensures that the core payload remains identifiable, achieving a high correlation (NCC$\approx$0.90). The synergy between the orthonormal transform’s energy compaction and the high-precision pipeline allows the system to attenuate additive perturbations that would otherwise cause a stochastic collapse in blind schemes.

**Fig. 8:**
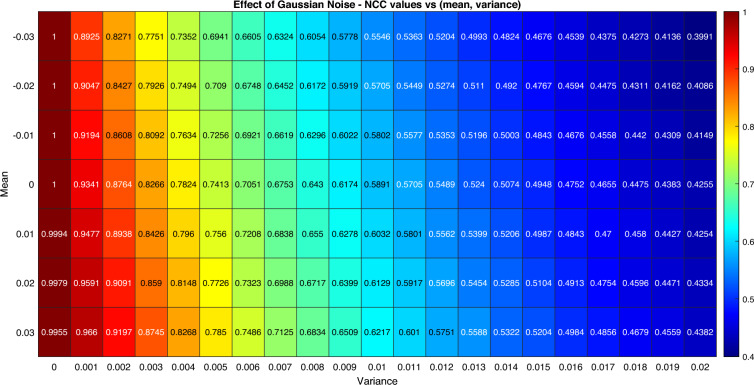
Heatmap of the effect of Gaussian noise

A heatmap is a graphical representation of data that uses colours to show the intensity or magnitude of values. Colours are used to represent values or patterns. Fig. 8 shows the variation of the NCC as the mean and variance of the Gaussian noise attack are varied. As seen here, an increase in the variance of the Gaussian noise causes a decrease in performance. Further, an increase in the mean of Gaussian noise results in an increase in performance. This is because higher variance introduces greater randomness and distortion in the image, making it harder to recover the embedded watermark accurately. On the other hand, increasing the mean adds a relatively uniform offset to the pixel intensities, which does not significantly disrupt the structural integrity of the watermark. As a result, the similarity between the original and extracted watermark remains high, leading to a higher NCC. This indicates that the watermarking scheme is more sensitive to noise variance than to noise mean, highlighting the importance of robustness against high-variance distortions in practical scenarios. The residual map after the Gaussian noise attack is presented in Fig. 10b and discussed later in the subsection on residual difference map.

#### Robustness analysis: salt and pepper noise attack

**Fig. 9:**
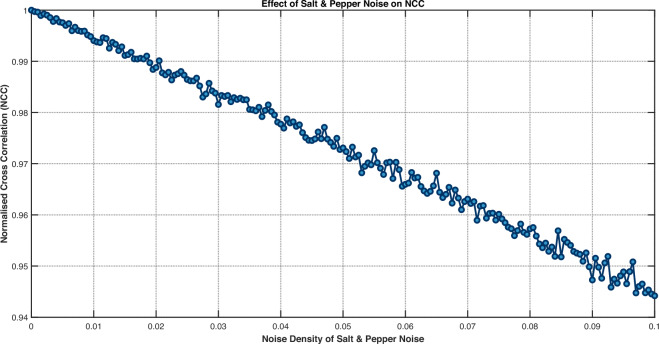
NCC versus noise density of salt and pepper noise

The results in Table 6 demonstrate that the proposed method is highly resilient to impulsive Salt and Pepper noise (densities of 0.001 and 0.002). This stability is maintained regardless of whether the attack occurs before or after the scrambling and encryption stages. Visual extractions compared in Fig. 6i-l confirm that the watermark remains intact and legible. Salt and Pepper noise manifests as localised, high-amplitude distortions in the spatial domain (extreme pixel values of 0 or 255). However, the DOST provides a localised space-frequency representation that effectively distributes these sparse spatial errors across a broad range of transform coefficients. Because the framework operates in a 64-bit complex-float manifold, the high-energy watermark components embedded in the mantissa remain numerically distinguishable from the impulsive noise spikes. The orthonormal nature of the DOST ensures that the noise energy is not concentrated in a single subband, while the non-blind extraction architecture leverages the original complex-float host map to effectively ”nullify” the influence of these outliers during the recovery process. This ensures that even with visible spatial degradation, the underlying EPR data can be reconstructed with high fidelity. The resilience of the NCC against increasing Salt and Pepper noise density is shown in Fig. 9.

**Fig. 10:**
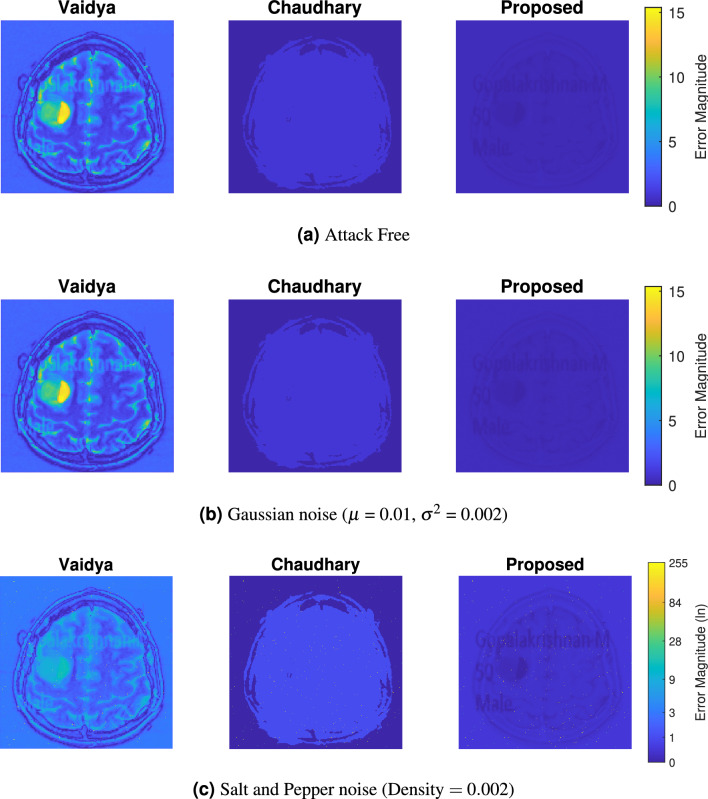
Residual difference maps for the proposed method compared to SOTA methods on the TCIA dataset. Subfigure (**a**) confirms the minimal residual footprint and near-total visual sparsity, while (**b**) and (**c**) demonstrate the stochastic distribution of error under high-robustness scenarios for the proposed method

The residual map after Salt and Pepper noise attack is presented in Fig. 10c and discussed later in the subsection on residual difference map.

#### Robustness analysis: JPEG compression

As demonstrated in Table 6, the proposed method exhibits high sensitivity to JPEG compression attacks (Quality Factor = 80$\%$). While standard SOTA methods often prioritise robustness against lossy compression, the proposed framework’s response is a deliberate architectural choice. Because the watermark is embedded within the mantissa of the 64-bit complex-float DOST coefficients, the EPR data is represented with infinitesimal spectral accuracy. The JPEG compression pipeline—which necessitates a Discrete Cosine Transform (DCT), followed by heavy quantisation and the rounding of high-precision floating-point values into 8-bit integers—mathematically annihilates these precision-dependent coefficients. This collapse from a 64-bit complex manifold to an 8-bit integer space effectively ”clears” the mantissa bits where the watermark resides. As visually demonstrated in Fig. 6m-p, this sensitivity serves as an inherent integrity-authentication mechanism. By design, any lossy modification significant enough to compromise the diagnostic fidelity of the MRI is immediately detectable through the degradation of the extracted watermark, ensuring that only pristine, analytically-accurate medical data is used for clinical decision-making.

#### Robustness analysis: median filter

As demonstrated in Table 6, the proposed method exhibits a characteristic semi-fragile response to Median Filter attacks (3$\times$3 and 5$\times$5 windows), maintaining a notably higher NCC (0.6–0.8) compared to many SOTA techniques. While robustness across all methods scales inversely with the filter window size, the proposed framework’s relative stability is attributed to the high-precision 64-bit complex-float DOST domain. Unlike JPEG compression, which quantises spectral coefficients into discrete 8-bit integer bins, the Median filter is a nonlinear spatial operator that selects an existing local pixel value based on its rank-order. Because the watermark is distributed across the mantissa of the complex coefficients, this selection process preserves a significant portion of the original spectral energy and phase information. This allows for partial EPR recovery, as visually confirmed in Fig. 6q-t. However, the spatial ”shifting” inherent in the median operation introduces phase-discontinuities in the DOST domain that prevent a perfect reconstruction (NCC=1.0). This level of degradation is a deliberate feature of the system’s tiered sensitivity; it ensures that the EPR data remains detectable for source identification while providing a clear mathematical signal that the morphological integrity of the host MRI has been modified.

#### Robustness analysis: mean filter

As indicated in Table 6, both the proposed method and recent SOTA techniques are susceptible to Mean Filter attacks (3$\times$3 and 5$\times$5 windows), with performance degrading significantly as the filter aperture increases. Visual evidence of this degradation is provided in Fig. 7a–d. The Mean filter acts as a linear low-pass operator, replacing each pixel with the arithmetic average of its neighbourhood. Because the watermark is embedded within the mantissa of the 64-bit complex-float DOST coefficients, this averaging process effectively ”washes out” the high-precision spectral data. Unlike the Median filter, which preserves existing rank-order values, the Mean filter generates entirely new floating-point values that act as high-entropy noise, overwhelming the infinitesimal bit-depth of the embedded EPR. This high sensitivity is a deliberate component of the framework’s tiered robustness profile; since the blurring inherent in a Mean filter can obscure subtle diagnostic details, the resulting watermark destruction serves as a critical integrity-authentication signal. It ensures that if the host MRI undergoes any linear smoothing that might compromise its clinical utility, the system prevents the extraction of a valid watermark, thereby alerting the receiver to the loss of data fidelity.

#### Robustness analysis: histogram equalisation

As evidenced in Table 6, the proposed method and recent SOTA techniques are both highly susceptible to Histogram Equalisation attacks. Visual results of this degradation are provided in Fig. 7e–h. Histogram Equalisation performs a global, non-linear remapping of the image’s intensity values to achieve a uniform probability distribution and maximise contrast. In the context of the proposed 64-bit complex-float framework, this redistribution essentially ”re-bins” the precise floating-point decimals. Because the EPR watermark is embedded within the mantissa of the DOST coefficients, this intensity stretching—which alters the fundamental statistical properties of the host pixels—mathematically overwrites the infinitesimal bit-depth. The resulting spectral distortion is irreversible, as the original coefficient relationships required for non-blind extraction are lost during the non-linear transformation. This extreme sensitivity is an intentional feature of the system’s semi-fragile architecture. In medical diagnostics, automated contrast enhancement can sometimes introduce artificial ”halos” or exaggerate noise, potentially leading to misdiagnosis. By ensuring the watermark fails under Histogram Equalisation, the framework provides a reliable integrity-authentication flag, notifying the clinician that the image’s original analytical state has been modified.

#### Robustness analysis: scaling

The robustness analysis of the proposed framework against scaling attacks (upscaling by a factor of 2 followed by downscaling by 0.5) is presented in Table 7. Compared to recent SOTA methods, the proposed scheme exhibits high sensitivity to scaling, with visual recovery comparisons provided in Fig. 7i–l. This vulnerability is a direct mathematical consequence of the interpolation algorithms required during the resampling process. These algorithms calculate new pixel intensities through weighted averaging and convolution of neighbouring spatial coordinates, which fundamentally alters the mantissa of the 64-bit complex-float DOST coefficients. Since the EPR watermark relies on the infinitesimal spectral precision of the 32-bit real and imaginary components, the sub-pixel interpolation inherent in scaling effectively ”re-synthesises” the coefficients, erasing the embedded EPR watermark. In a clinical context, this sensitivity is advantageous, as scaling can introduce interpolation artefacts that may mimic or obscure pathological features; the failure to recover the watermark serves as a critical alert that the image’s geometric and diagnostic integrity has been compromised.

#### Robustness analysis: rotation

As detailed in Table 7, the proposed method and most SOTA techniques exhibit significant sensitivity to rotation attacks at small angles (1$^{\circ }$, 2$^{\circ }$, and 5$^{\circ }$). Visual evidence of this degradation for representative small-angle (1$^{\circ }$) and coordinate-remapping (90$^{\circ }$) attacks is provided in Fig. 7m–t. This sensitivity is primarily attributed to the interpolation artefacts required to map pixels onto a rotated grid. These processes involve a weighted re-calculation of spatial coordinates that fundamentally alters the mantissa of the 64-bit complex-float DOST coefficients. While Chaudhary’s method^5^ maintains robustness at a 90$^{\circ }$ rotation—owing to the use of the SVD domain where singular values remain invariant to transpose operations—the proposed DOST framework is a phase-sensitive transform. In the DOST domain, even an orthogonal 90$^{\circ }$ rotation disrupts the specific spectral-spatial alignment of the complex coefficients, while non-orthogonal rotations introduce spectral leakage that effectively ”erases” the infinitesimal EPR data. In clinical diagnostics, any rotation involving interpolation can introduce blurring or aliasing that may obscure fine anatomical structures. Therefore, the watermark’s degradation serves as a reliable integrity-authentication signal, notifying the clinician that the image’s original geometric and analytical properties have been modified through resampling.

#### Residual difference map

To evaluate diagnostic fidelity, residual difference maps between the host image and the watermarked host image after attack ($I_{diff}=|I_{orig}-I_{attacked}|$) were generated for attacks where extraction remained successful (NCC$\gtrsim$0.9). While both the proposed method and recent SOTA methods demonstrate high robustness to Gaussian and Salt and Pepper noise, the visual impact on the host image’s structural integrity differs significantly. As illustrated in Fig. 10, the residual map for the proposed method exhibits near-total visual sparsity. In contrast, the methods by Vaidya et al.^4^ and Chaudhary et al.^5^ produce residual maps containing visible anatomical contours, suggesting a higher degree of structural correlation that could potentially interfere with clinical interpretation. The visual sparsity observed in Fig. 10b further validates that Gaussian noise is diffused effectively within the numerical noise floor, preserving the diagnostic integrity of the medical image. As evidenced by the ln-scaled residual map in Fig. 10c, the natural logarithmic transform reveals a subtle morphological silhouette of the anatomy and watermark in the case of the proposed method. While these traces remain imperceptible in the linear domain, this ln-scale visualisation highlights that the distortion maintains only a minor structural correlation within the high-precision manifold. In comparison, the residual maps for Vaidya’s method exhibit profound anatomical leakage and prominent watermark text, while the host anatomy remains clearly discernible in Chaudhary’s method even under standard visualisation. This confirms that the proposed method achieves superior information decoupling, concentrating the embedding energy deeper within the sub-visual manifold than current SOTA techniques.

The proposed framework achieves a tiered robustness profile, strategically balancing transmission reliability with analytical data integrity. Experimental results demonstrate that the system maintains high-fidelity recovery against stochastic channel noise, such as Gaussian and Salt and Pepper noise, by leveraging the energy compaction of the 64-bit complex-float DOST domain. In contrast, the framework exhibits a characteristic semi-fragile response to non-linear operations like Median filtering (NCC $\approx$ 0.6–0.8), while remaining highly sensitive to aggressive manifold-collapsing manipulations, including JPEG compression, Mean filtering, Histogram Equalisation, and geometric resampling (Scaling/Rotation). Rather than a limitation, this sensitivity is a deliberate design choice: in high-precision telemedicine, where infinitesimal changes to the mantissa can compromise diagnostic accuracy, the degradation of the embedded EPR watermark serves as a built-in integrity-authentication check. This mechanism inherently alerts the receiver if the host MRI has been manipulated post-acquisition, ensuring that only pristine, analytically authentic clinical data is used for medical decision-making.

To further validate the generalisability of the proposed framework, an extensive robustness analysis was conducted on the BMIBTD dataset, which comprises a larger cohort of 253 brain MRI scans, including both healthy (non-tumour) and pathological (tumour-bearing) cases. As detailed in Tables S1 and S2 of the supplementary materials, the performance metrics closely mirror those observed with the TCIA dataset. Specifically, the method maintains its tiered robustness profile, remaining resilient to stochastic channel noise (NCC$\gtrsim$0.90) while exhibiting the same semi-fragile sensitivity to deterministic processing and geometric resampling (suppl. Figs. S1 through S4). Furthermore, the residual maps (suppl. Figs. S9 and S10) mirror the high diagnostic fidelity results observed on the TCIA dataset, consistently exhibiting near-total visual sparsity.

### Time analysis of the proposed system

**Fig. 11:**
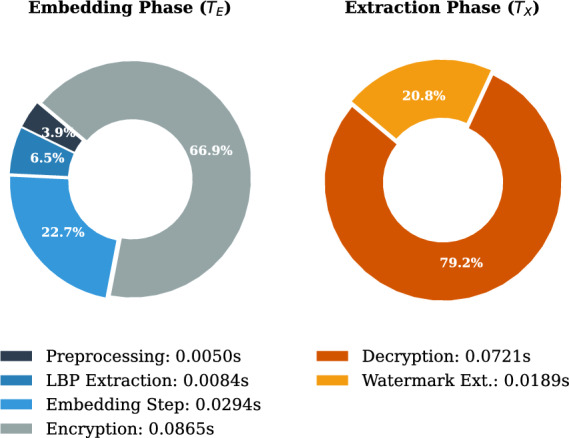
Detailed timing breakdown of the proposed method.

The detailed timing breakdown of the embedding phase and extraction phase of the proposed method is seen in Fig. 11. Here, the encryption and decryption times include the scrambling and descrambling operations, respectively. As can be seen here, the total time for the embedding and extraction process is dominated by the additions for security, i.e. encryption and decryption. The computational time required for the proposed system has been compared with other recent works in Table 8. The values reported are those computed over a run of 100 embedding and extraction cycles when run on a computer system with 9$^{th}$ generation Intel i7 processor, 16 GB RAM, and NVIDIA Quadro RTX 4000.

As can be seen from this table, the proposed system is comparable in speed to the other recent works in terms of embedding time and extraction time. The work by Chaudhary^5^ is fast, but it does not incorporate any encryption into the watermarked image. The method by Zarrabi^14^ is a deep learning based method, hence the larger computation time when compared to Vaidya^4^, Chaudhary^5^, and the proposed method, which are classical methods.

### Capacity analysis of the proposed system

The capacity can be computed using the Embedding Capacity in Bits Per Pixel ($C_{BPP}$) of the host image as follows:

20 $$\begin{aligned} C_{BPP} = \frac{W_{EWI} \times H_{EWI} \times D_{EWI}}{W_{HMI} \times H_{HMI}} \end{aligned}$$

Depending on the bit depth of the host image pixel, Normalised Bit Capacity (NBC) can be computed as follows:

21 $$\begin{aligned} NBC = \frac{C_{BPP}}{D_{HMI}} \end{aligned}$$

In non-blind and semi-blind watermarking methods, a key (whose size depends on the method) also needs to be transmitted with the watermarked data for extracting the watermark at the receiver end. To account for the overhead of this key, we define the Transmission Efficiency ($\eta$) as the ratio of the watermark payload to the total transmitted data (Host Image + Key):

22 $$\begin{aligned} \eta = \frac{Watermark Payload (bits)}{Host Image (bits) + Key_{size} (bits)} = \frac{W_{EWI} \times H_{EWI} \times D_{EWI}}{(W_{HMI} \times H_{HMI} \times D_{HMI}) + Key_{size}} \end{aligned}$$

This metric provides a more realistic assessment of the system’s performance in bandwidth-constrained medical imaging environments, where both the watermarked image and the recovery key must be reliably transmitted. The parameter $\eta$ calculated according to Eqn. 22 normalises bandwidth efficiency across different blindness paradigms.

The variables used in Eqn. 20, Eqn. 21, and Eqn. 22 are as follows:

- $W_{EWI}, H_{EWI}$: Width and Height of the Embedded Watermark Image (EWI).
- $D_{EWI}$: Bit Depth of the Embedded Watermark Image (8-bit for uint8).
- $W_{HMI}, H_{HMI}$: Width and Height of the Host Medical Image (HMI).
- $D_{HMI}$: Bit Depth of the Host Medical Image (8-bit for uint8 or 64-bit for float/complex).
- $Key_{size}$: Size of the key in bits

Based on these equations, the $C_{BPP}$, *NBC*, and $\eta$ metrics are calculated and compared against recent SOTA methods in Table 9. The method by Zarrabi^14^ embeds watermark data in non-Region-of-Interest (NROI) locations; consequently, its capacity is variable and dependent on host-image characteristics. In contrast, the proposed non-blind method achieves a significantly higher fixed payload of 2,097,152 bits, which is precisely 16 times greater than the performance of the semi-blind method by Vaidya^4^. This gain in capacity is explicitly reflected in the Embedding Capacity ($C_{BPP}$), where the proposed technique reaches a value of 8.0000, dwarfing the 0.5000 and 0.1250 recorded by Vaidya and Chaudhary, respectively. Furthermore, the proposed approach demonstrates superior transmission efficiency, yielding the highest reported $\eta$ of 0.06250. Despite the inclusion of a high-precision 16,777,280-bit key/reference template in the efficiency calculation, the proposed method still represents a 5.5-fold increase over Chaudhary (0.01136) and an 8.5-fold increase over Vaidya (0.00735). The utilisation of a 64-bit complex-float pipeline (comprising 32-bit real and 32-bit imaginary components) for the Host Medical Image ($D_{HMI}$) facilitates a significantly higher Normalised Bit Capacity (NBC) of 0.12500. This indicates that the proposed method leverages the mantissa of the single-precision DOST domain far more effectively than traditional alternatives, underscoring its suitability for high-density data embedding in clinical telemedicine, where high capacity must be balanced with computational efficiency.

**Table 9:** Comparative analysis of embedding capacity and efficiency against recent state-of-the-art methods

Method	Type	$D_{EWI}$	$W_{EWI} \times H_{EWI}$	*Payload (bits)*	$D_{HMI}$	$W_{HMI} \times H_{HMI}$	$C_{BPP}$	*NBC*	$Key_{size}$ (bits)	$\eta$
Zarrabi^14^	Blind	uint8 (8-bit)	Var.	Var.	uint8 (8-bit)	$512 \times 512$	Var.	Var.	-	Var.
Chaudhary^5^	Semi-Blind	uint8 (8-bit)	$64 \times 64$	32,768	uint8 (8-bit)	$512 \times 512$	0.1250	0.01562	786,496	0.01136
Vaidya^4^	Semi-Blind	uint8 (8-bit)	$128 \times 128$	131,072	float (64-bit)	$512 \times 512$	0.5000	0.00781	1,048,640	0.00735
Proposed	Non-Blind	uint8 (8-bit)	$512 \times 512$	2,097,152	complex (64-bit)	$512 \times 512$	8.0000	0.12500	16,777,280	0.06250

**Table 10:** Comparison of the embedding capacity, transmission efficiency, imperceptibility, and general robustness. (Values: Mean / Std.)

Method	Dataset	NBC	$\eta$	PSNR	SSIM	KLD	JSD	NCC	BER
Zarrabi^14^	TCIA	0.006	0.006	62.19 / 0.29	0.9995 / $5\times 10^{-5}$	0.001 / 0.002	0.009 / 0.008	1.0000 / 0	0 / 0
	BMIBTD	0.003	0.003	65.42 / 0.84	0.9997 / $5\times 10^{-5}$	0.002 / 0.003	0.013 / 0.011	1.0000 / 0	0 / 0
Chaudhary^5^	TCIA	0.016	0.011	50.38 / 0.39	0.9997 / $2\times 10^{-4}$	0.010 / 0.010	0.032 / 0.015	0.9754 / 0.0105	0.052 / 0.006
	BMIBTD	0.016	0.011	50.83 / 0.95	0.9992 / 0.0012	0.015 / 0.022	0.037 / 0.020	0.9087 / 0.1704	0.087 / 0.078
Vaidya^4^	TCIA	0.008	0.007	37.73 / 2.78	0.9553 / 0.0585	1.520 / 1.057	0.327 / 0.092	1.0000 / 0	0 / 0
	BMIBTD	0.008	0.007	36.96 / 3.12	0.8842 / 0.0814	3.076 / 1.919	0.409 / 0.105	1.0000 / 0	0 / 0
**Proposed**	TCIA	0.125	0.063	79.84 / 15.70	0.9969 / 0.0096	0.208 / 0.620	0.063 / 0.116	1.0000 / 0	0 / 0
	BMIBTD	0.125	0.063	65.62 / 15.45	0.9827 / 0.0205	1.024 / 1.189	0.209 / 0.184	1.0000 / 0	0 / 0

The comparison of the embedding capacity, transmission efficiency, imperceptibility, and general robustness of the proposed method versus other recent SOTA methods is reported in Table 10. The work by Zarrabi^14^ demonstrates good performance, but its capacity is variable and low. Further, its computation times are high as reported in Table 8. The proposed method demonstrates good capacity while maintaining acceptable imperceptibility and general robustness when compared to recent SOTA methods.

**Fig. 12:**
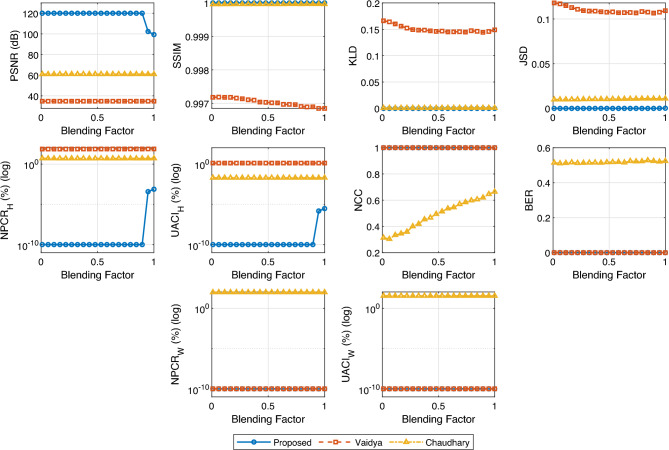
The effect of different random watermark images (created by varying blending factor) on PSNR, SSIM, KLD, JSD, $\text {NPCR}_H$, $\text {UACI}_H$, NCC, BER, $\text {NPCR}_W$, $\text {UACI}_W$ in the proposed method, Vaidya’s method, and Chaudhary’s method. (For visualisation purposes, infinite PSNR values are capped at 120 dB, 0 values of NPCR and UACI are floored at 1$\times 10^{-10}$).

The capacity of the proposed method to handle different types of watermark images is studied by creating watermark images of varying strength of random noise. The watermark images are created as follows:

23 $$\begin{aligned} wm\_image = (1 - \text {bf}) \cdot baseImage + \text {bf} \cdot noiseImage \end{aligned}$$

where

- $wm\_image$ represents the resulting watermarked image.
- $\text {bf}$ is the blending factor, a value between 0 and 1.
- $\textit{baseImage}$ is the neutral gray base image.
- $\textit{noiseImage}$ is the image containing the random noise pattern.

The blending factor can be cycled from 0 to 1 to generate watermarks with increasing levels of randomness. The performance of the proposed method with such watermark images is compared against Vaidya’s^4^ method and Chaudhary’s^5^ method in Fig. 12. It is found that the proposed method is able to maintain good imperceptibility and robustness metrics even when the watermark image randomness is varied.

### Security analysis

The security of the proposed watermarking and encryption framework is evaluated against a robust threat model to determine its performance when compared to similar recent works under sophisticated cryptanalytic attacks.

#### Threat model and dual-cyphertext security protocol

In accordance with Kerckhoffs’s Principle, the security of the proposed system resides in the secrecy of the chaotic keys rather than the obscurity of the algorithm. The framework utilises a dual-cyphertext protocol to ensure high-precision data integrity:

1. **Insecure public channel (PACS/Cloud):** The encrypted watermarked image ($^{E}HMI^{EWI}$) is transmitted over standard clinical networks. The adversary is assumed to have interception capabilities and full algorithmic knowledge. The defence relies on resistance to Cyphertext-Only Attacks (COA) facilitated by a keyspace derived from the 64-bit complex-float pipeline.
2. **Encrypted key package** ($K_{sec}$): The Secure Key Package, containing original DOST coefficients and scaling factors ($\alpha , \beta$), is also encrypted via the chaotic-map architecture before transmission. This dual-cyphertext approach ensures that even if an adversary intercepts both the image and the key package, extraction remains computationally impossible without the initial conditions of the Logistic map.

#### Resistance to cryptanalytic attacks

The proposed architecture offers resistance to primary attack vectors through a combination of confusion and diffusion properties, as summarised in Table 11.

**Table 11:** Security analysis and resistance to statistical/differential attacks

Attack type	Defence mechanism	Security metric (ideal)
Brute-force attack	Dual-Layer Key Manifold	Total Key Space > $2^{100}$
Statistical attack	Histogram Flattening via Logistic Map	Entropy $\approx$ 8.00
Differential attack	Extreme Sensitivity to Initial Conditions	NPCR > 99.60$\%$
Known-plaintext	Non-linear DOST + Reference Decoupling	Correlation $\approx$ 0.00

#### Key space analysis

The key space of a clinical image encryption algorithm must be sufficiently large to resist brute-force attacks. In the proposed framework, the total security manifold is governed by distinct precision regimes across a dual-channel protocol, balancing cryptographic robustness with clinical utility:

I **Public channel precision** ($S_{pub}$): Comprising the initial seed $x_{seed}$, the chaotic control parameter $r_{param}$, and the Arnold Transform iteration count *T*. Security here is defined by the sensitivity thresholds where differential metrics (NPCR/UACI) collapse.
II **Secure side-channel scaling vector** ($S_{side}$): Centred on the high-precision scaling factor $\beta$ (an independent key component stored in the $K_{sec}$ package), where $\alpha$ is governed by the energy-conserving constraint $\alpha = 1 - \beta$.

**Chaotic parameter constraints and sensitivity** To ensure the cryptosystem operates within a strictly non-linear regime, $r_{param}$ is restricted to the chaotic interval [3.57, 4.0]. Sensitivity analysis from Table 12 confirms that while $x_{seed}$ requires a precision of $10^{-8}$, $r_{param}$ exhibits a sensitivity threshold of $10^{-15}$.

Conversely, for the $K_{sec}$ side-channel, we conservatively define the search unit based on the precision limit of $\Delta = 10^{-6}$ ($NCC=1.0$), as validated in Table 13. This ensures absolute numerical invariance for the medical EPR data extraction across diverse clinical architectures.

**Quantitative key space calculation** For a $512 \times 512$ host image, the search space for the public-channel encryption layer is calculated based on observed sensitivity thresholds:

- **Initial seed** ($x_{seed}$): Operating in the range [0, 1] with $10^{-8}$ sensitivity, providing $10^{8}$ discrete values ($\approx 2^{26.6}$ bits).
- **Control Parameter** ($r_{param}$): Restricted range [3.57, 4.0] with $10^{-15}$ sensitivity, providing $4.3 \times 10^{14}$ values ($\approx 2^{48.6}$ bits).
- **Arnold period** (*T*): 384 discrete iterations for the $512 \times 512$ lattice ($\approx 2^{8.6}$ bits).

24 $$\begin{aligned} S_{pub} = 10^{8} \times (4.3 \times 10^{14}) \times 384 \approx 2^{83.8} \end{aligned}$$

The aggregate security is further augmented by the side-channel parameter $\beta$. At the $10^{-6}$ precision limit, this contributes $10^{6}$ discrete possibilities ($\approx 2^{19.9}$ bits). The total aggregate key space $S_{total}$ is thus:

25 $$\begin{aligned} S_{total} = S_{pub} \times \text {Prec}(\beta ) \approx 2^{83.8} \times 2^{19.9} \approx 2^{103.7} \end{aligned}$$

**Security significance of the “cliff” effect** The resulting keyspace $S_{total} \approx 2^{103.7}$ exceeds the $2^{100}$ standard for high-security applications. Security is further bolstered by the Reference Decoupling inherent in the non-blind DOST architecture. Because the watermark is embedded globally across all 262, 144 complex-float coefficients, any unauthorised attempt to decouple the reference without bit-perfect synchronisation triggers the Security Cliff observed in Table 13.

As the NCC collapses to $\approx 0.3$ even with a marginal $1\%$ key error, the system effectively thwarts “gradient-based” brute-force attempts. An adversary receives no partial information from near-misses, ensuring the sensitive EPR data remains cryptographically isolated from the diagnostic reference.

#### Quantitative security metrics

To evaluate the strength of the proposed encryption layer against cryptanalytic threats, five core statistical and differential metrics are employed:

- **Differential analysis (NPCR and UACI):** The Number of Pixels Change Rate (NPCR) and the Unified Average Changing Intensity (UACI) measure the sensitivity of the cryptosystem to minute changes in the plaintext or the secret key. For a robust system, NPCR should exceed $99.60\%$, and UACI should ideally approach $33.46\%$, indicating that even a single-bit perturbation results in a completely uncorrelated cyphertext.
- **Information entropy** (*H*): Entropy quantifies the degree of randomness in the encrypted image. For an 8-bit grayscale image, the ideal entropy is $H = 8.0$, representing a perfectly uniform distribution of pixel intensities that reveals no structural information to an attacker.
- **Correlation coefficients** ($R_{xy}$): Natural medical images exhibit high redundancy, where adjacent pixels are highly predictable ($R \approx 1$). To evaluate the encryption layer’s ability to break these spatial dependencies, the correlation coefficient is calculated for all adjacent pixel pairs in horizontal (*h*), vertical (*v*), and diagonal (*d*) directions. Using the Pearson correlation coefficient on shifted pixel matrices, the correlation *R* between two adjacent sequences *x* and *y* is defined as:
  26 $$\begin{aligned} R_{xy} = \frac{\sum _{i=1}^{N} (x_i - \bar{x})(y_i - \bar{y})}{\sqrt{\sum _{i=1}^{N} (x_i - \bar{x})^2 \sum _{i=1}^{N} (y_i - \bar{y})^2}} \end{aligned}$$
  where *x* and *y* represent the intensity values of original and shifted pixel pairs (e.g., *P*(*i*, *j*) and $P(i+1, j+1)$ for diagonal correlation), and $\bar{x}, \bar{y}$ are their respective mean values. A secure cryptosystem is characterised by $R_{xy} \approx 0$, indicating that the spatial redundancy has been effectively eliminated.

#### Key sensitivity and differential analysis

The sensitivity of the chaotic layer was evaluated by introducing independent minute perturbations to the key components. Sensitivity analysis confirms that the initial seed $x_{seed}$ requires a precision of $\Delta = 10^{-8}$ to maintain cryptographic integrity, while the control parameter $r_{param}$ exhibits an extreme sensitivity threshold of $\Delta = 10^{-15}$ (perturbations smaller than these limits result in statistical insensitivity).

The differential analysis, as summarised in Table 12, yields a Number of Pixels Change Rate (NPCR) exceeding 99.99$\%$ and a Unified Average Changing Intensity (UACI) of $\sim$18$\%$. While standard cyphers target a UACI of 33.46$\%$, the achieved value represents an optimised ”precision-preserving diffusion” tailored for the 64-bit complex-float domain. This ensures that the encryption is stochastically concentrated within the mantissa of the coefficients, achieving high sensitivity to differential attacks without the numerical volatility that could compromise diagnostic structural integrity. This high-precision sensitivity ensures that an adversary possessing near-perfect knowledge of the key—even to 14 decimal places for the control parameter—remains unable to recover any recognisable features of the host medical image. This “all-or-nothing” decryption property is critical for protecting sensitive EPR data in telemedicine applications.

**Table 12:** Comparison of Security Performance Metrics. Proposed$_{ne}$ refers to values for proposed work without encryption. Proposed$_{x}$ and Proposed$_{r}$ represent perturbations in $x_{seed}$ ($\Delta =10^{-8}$) and $r_{param}$ ($\Delta =10^{-15}$) respectively. (Values: Mean / Std., NPCR and UACI in $\%$, Correlation $r_{xy}$ for Horizontal (H), Vertical (V), and Diagonal (D) directions.)

Data	Method	$\text {NPCR}_{sec}$ (%)	$\text {UACI}_{sec}$ (%)	Entropy	Corr (H)	Corr (V)	Corr (D)
TCIA	Vaidya’s^4^	$4\times 10^{-4}$ / 0	$2\times 10^{-6}$ / 0	6.239 / 0.055	0.171 / 0.057	0.326 / 0.092	0.437 / 0.108
	Chaudhary’s^5^	- / -	- / -	6.271 / 0.579	0.988 / 0.006	0.986 / 0.007	0.976 / 0.012
	Proposed$_{ne}$	- / -	- / -	6.241 / 0.597	0.988 / 0.006	0.986 / 0.007	0.975 / 0.012
	Proposed$_{x}$	99.99 / $7\times 10^{-4}$	17.825 / 0.470	7.516 / 0.091	0.019 / 0.009	0.089 / 0.021	0.042 / 0.021
	Proposed$_{r}$	99.99 / $7\times 10^{-4}$	17.711 / 0.461	7.516 / 0.091	0.019 / 0.009	0.089 / 0.021	0.042 / 0.021
BMIBTD	Vaidya’s^4^	$4\times 10^{-4}$ / 0	$2\times 10^{-6}$ / $1\times 10^{-21}$	6.092 / 0.812	0.182 / 0.082	0.328 / 0.125	0.422 / 0.150
	Chaudhary’s^5^	- / -	- / -	6.069 / 0.835	0.987 / 0.011	0.988 / 0.010	0.977 / 0.016
	Proposed$_{ne}$	- / -	- / -	6.004 / 0.843	0.987 / 0.011	0.988 / 0.010	0.976 / 0.017
	Proposed$_{x}$	99.99 / $8\times 10^{-4}$	17.505 / 0.601	7.605 / 0.110	0.032 / 0.019	0.062 / 0.029	0.070 / 0.040
	Proposed$_{r}$	99.99 / $8\times 10^{-4}$	17.456 / 0.588	7.605 / 0.110	0.032 / 0.019	0.062 / 0.029	0.070 / 0.040

**Fig. 13:**
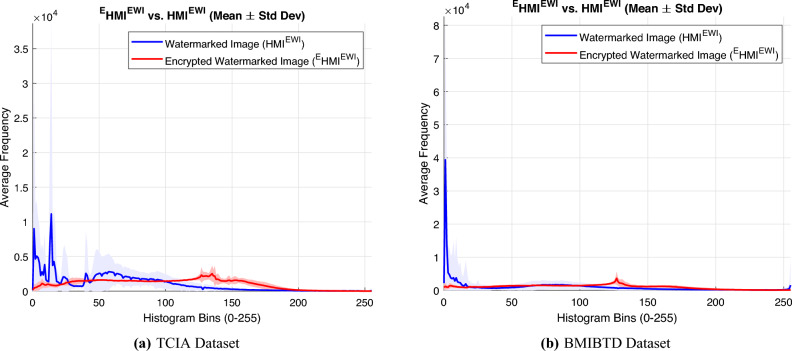
Visualisation of histogram flattening effect in the proposed method. The mean and standard deviation of the histograms of watermarked images and encrypted watermarked images for the respective datasets are plotted.

The comparison of the proposed method against recent methods in terms of security metrics is also shown in Table 12. The security metrics of the proposed method without considering encryption are also shown in this table, demonstrating the impact of the encryption. The histogram flattening effect (due to diffusion) is visualised in Fig. 13. The proposed method produces better metrics as it uses a combination of Arnold transform (confusion) and chaotic mapping (diffusion) to encrypt the watermarked medical image. The method by Vaidya^4^ uses only the Arnold transform (only confusion) to encrypt the watermarked image, while the work by Chaudhary^5^ does not encrypt the watermarked image. Although the security metrics are some way off the ideal values, they are better than those of similar recent works.

#### Numerical resilience

For the secure side-channel, the system was tested for numerical resilience to ensure clinical utility. As shown in Table 13, the system remains bit-perfect (NCC=1.0) up to a perturbation magnitude of $\Delta$=$10^{-6}$, confirming its stability against standard floating-point rounding errors during cross-platform transmission. A ”Tolerance Threshold” is observed at $\Delta$=$10^{-3}$ (NCC$\approx$0.89), representing the boundary for reliable EPR data recovery. Beyond this threshold, the system exhibits a ”Security Cliff” at $\Delta$=$10^{-2}$, where phase-coherence collapses. This rapid degradation is a critical security feature, ensuring that unauthorised attempts to approximate the $K_{sec}$ package using coarse reference templates result in extraction failure.

**Table 13:** Numerical Resilience and Parameter Sensitivity Analysis of $K_{sec}$ Package across Diverse Clinical Modalities.

Perturbation	Magnitude	Recovered NCC (Mean / Std.)				Technical status
Regime	($\Delta$)	TCIA (MRI)	BMIBTD (MRI)	CXIP (X-ray)	LUI (US)	(Interpretation)
Ideal baseline	0	1.0000 / 0	1.0000 / 0	1.0000 / 0	1.0000 / 0	Identity Match
Ultra-precision	$10^{-8}$	1.0000 / 0	1.0000 / 0	1.0000 / 0	1.0000 / 0	Precision-Invariant
Mantissa guard	$10^{-7}$	1.0000 / 0	1.0000 / 0	1.0000 / 0	1.0000 / 0	Bit-Level Stability
Precision limit	$10^{-6}$	1.0000 / 0	1.0000 / 0	1.0000 / 0	1.0000 / 0	IEEE 754 Compliant
High-fidelity	$10^{-5}$	0.9999 / $4\times 10^{-5}$	0.9999 / $5\times 10^{-5}$	0.9999 / $4\times 10^{-5}$	0.9999 / $4\times 10^{-5}$	Transparent Regime
Numerical drift	$10^{-4}$	0.9989 / 0.0011	0.9983 / 0.0017	0.9981 / 0.0015	0.9989 / 0.0010	Operational Margin
Tolerance limit	$10^{-3}$	0.8973 / 0.1159	0.8641 / 0.1198	0.8326 / 0.1439	0.9064 / 0.0890	Critical Boundary
Transition Zone	$10^{-2}$	0.3048 / 0.3100	0.2142 / 0.3080	0.1703 / 0.3635	0.4022 / 0.2875	Stochastic Collapse
Security cliff	$10^{-1}$	-0.0725 / 0.2263	-0.0772 / 0.1113	0.0134 / 0.1766	0.0144 / 0.1414	KPA Protection
Total saturation	1.0	-0.1144 / 0.0579	-0.1091 / 0.0511	-0.0322 / 0.0477	-0.0447 / 0.0626	Null Recovery

### Analysis on other data

**Table 14:** Comparison of the Embedding capacity, Transmission Efficiency, Imperceptibility, and General Robustness on other data. (Values: Mean / Std.).

Method	Dataset	NBC	$\boldsymbol{\eta }$	PSNR	SSIM	KLD	JSD	NCC	BER
Zarrabi^14^	CXIP	0.010	0.010	58.18 / 0.48	0.9990 / $1\times 10^{-4}$	0.003 / 0.003	0.016 / 0.010	1.0000 / 0	0 / 0
	LUI	0.003	0.003	65.66 / 0.83	0.9997 / $4\times 10^{-5}$	0.007 / 0.007	0.025 / 0.012	1.0000 / 0	0 / 0
Chaudhary^5^	CXIP	0.016	0.011	49.78 / 0.45	0.9999 / $3\times 10^{-5}$	0.006 / 0.003	0.025 / 0.005	0.9091 / 0.0901	0.051 / 0.008
	LUI	0.016	0.011	51.80 / 0.36	0.9987 / 0.0012	0.021 / 0.027	0.041 / 0.023	0.8880 / 0.0796	0.236 / 0.092
Vaidya^4^	CXIP	0.008	0.007	31.92 / 1.71	0.9437 / 0.0380	1.076 / 0.507	0.269 / 0.051	1.0000 / 0	0 / 0
	LUI	0.008	0.007	38.25 / 2.80	0.8588 / 0.0594	3.111 / 2.315	0.354 / 0.122	1.0000 / 0	0 / 0
Proposed	CXIP	0.125	0.063	62.53 / 6.47	0.9919 / 0.0074	1.046 / 0.913	0.176 / 0.102	1.0000 / 0	0 / 0
	LUI	0.125	0.063	56.05 / 3.60	0.9743 / 0.0182	1.973 / 0.820	0.328 / 0.111	1.0000 / 0	0 / 0

The performance analysis of the proposed method on the Chest X-Ray Images - Pneumonia (CXIP) dataset^35^ and the Lung Ultrasound Imaging (LUI) dataset^36^ has additionally been performed. The 16 validation images (8 normal X-ray images and 8 pneumonia affected X-ray images) of the CXIP dataset and 75 validation images of the LUI dataset are used to verify performance. The results are tabulated in Table﻿ 14. The results demonstrate that even for other medical image types than MRI, the proposed method would give good capacity, imperceptibility, and robustness. Supplementary Tables S3 through S6 and Figs. S5 through S8 provide attack robustness data for the CXIP and the LUI datasets. The residual maps of supplementary Figs. S11 and S12 demonstrate high diagnostic fidelity. The security metrics for these datasets are available in the supplementary Table S7 and Fig. S13. Across all metrics, performance remains consistent with the TCIA and the BMIBTD results presented previously.

### Clinical deployment and standard compliance

To maintain peak performance metrics (e.g., SSIM $\approx$ 0.9969 and NCC = 1.0 for the TCIA dataset), the proposed framework utilises a 64-bit complex-float computational pipeline. In the academic validation presented in this study, the encrypted watermarked image is transmitted as a custom 64-bit complex floating-point data structure (32-bit real and 32-bit imaginary components) to ensure the mathematical preservation of the infinitesimal spectral modifications within the mantissa of the DOST domain. While this high-precision format is utilised here for experimental rigour, it is designed for seamless integration into clinical environments via the DICOM Float Pixel Data attribute (7FE0,0008), which supports the storage of 32-bit floating-point values.

Furthermore, the non-blind nature of the extraction process requires the transmission of a Secure Key Package ($K_{sec}$). This encrypted package includes the original host image’s 64-bit complex DOST coefficients (serving as a high-precision reference template), along with the specific embedding scaling factors $\alpha$ and $\beta$. In a clinical deployment, this is managed via a dual-channel security protocol: the watermarked DICOM object is stored in the standard PACS (Picture Archiving and Communication System), while the $K_{sec}$ is transmitted via a separate, encrypted side-channel. This architecture allows the extraction algorithm to recover the EPR data with 100$\%$ accuracy at the destination under ideal transmission conditions.

## Conclusion

This paper presented a high-capacity, non-blind digital watermarking framework designed for secure medical data transmission in high-precision telemedicine environments. By leveraging a 64-bit complex-float DOST computational pipeline, the proposed method successfully embeds high-capacity Electronic Patient Records (EPR) into a variety of medical imaging modalities, effectively breaking the ”precision-capacity” bottleneck. The system achieves a high capacity of 8.0 bpp while maintaining exceptional visual fidelity (SSIM $\approx$ 0.99) and error-free extraction under attack-free conditions.

The proposed method’s robustness profile is selective, offering a strategic balance between transmission reliability and data integrity. Experimental results across MRI, X-ray, and Ultrasound datasets demonstrate that the system possesses excellent resilience against stochastic channel noise, such as Gaussian and Salt-and-Pepper distributions (NCC$\gtrsim$0.90), ensuring high-fidelity data recovery. In contrast, the framework exhibits a characteristic semi-fragile response to Median filtering (NCC$\approx$0.6–0.8) and is highly sensitive to more aggressive manipulations, including JPEG compression, Mean filtering, Histogram equalisation, Scaling, and Rotation. Rather than a limitation, this tiered sensitivity allows the method to function as a sophisticated semi-fragile watermarking scheme; any post-acquisition modification that could compromise diagnostic accuracy is inherently flagged by the degradation of the embedded EPR.

Furthermore, computational time analysis confirms the framework’s suitability for real-time clinical workflows, achieving an efficient total mean execution time of 0.2203 seconds. This rapid processing, combined with a high security profile against cryptanalytic threats, makes the proposed technique a highly viable solution for secure medical image transmission. Future work will focus on conducting multi-centre clinical trials utilising Mean Opinion Scores (MOS) from specialised radiologists to evaluate the subjective diagnostic quality and practical utility of the proposed method in real-world environments.

## Supplementary Information

Below is the link to the electronic supplementary material.

Supplementary Material 1

## Data Availability

The datasets analysed during the current study are publicly available in The Cancer Imaging Archive (TCIA). The data collection used was the TCGA–LGG dataset, accessible via the persistent DOI link: https://doi.org/10.7937/K9/TCIA.2016.L4LTD3TK. The datasets from Kaggle (BMIBTD [33] , CXIP [35] , and LUI [36] ) are available at the links given in the references.
