# Supplementary material for: A fast and semi-fragile high-capacity digital watermarking system for MRI images using a hybrid combination of DOST and LBP features
Source: Sci Rep. 2026 May 5;16:20661. doi: 10.1038/s41598-026-50380-x (PMC13333918; doi:10.1038/s41598-026-50380-x)
Supplement: Supplementary file 1 — Supplementary Material 1 [file 41598_2026_50380_MOESM1_ESM.pdf]

Supplementary material for A Fast and Semi-Fragile High-Capacity Digital Watermarking System for MRI Images Using a Hybrid Combination of DOST and LBP Features

Nimmy George[1,*,@]**, Michael George**[1,*]**, and Manju Manuel**[2,*]

[1]Department of Electronics and Communication Engineering, Rajiv Gandhi Institute of Technology, Kottayam, Kerala, 686501, India.
[2]Department of Electronics and Communication Engineering, Government Engineering College, Painavu, Idukki, 685603, India
[*]Affiliated to APJ Abdul Kalam Technological University, Kerala, India.
[@]nimmygeorge@rit.ac.in

ABSTRACT

In telemedicine, digital watermarking is critical for safeguarding patient privacy by securely embedding sensitive Electronic Patient Records (EPR) directly into medical imagery. This work proposes a high-capacity, non-blind, and semi-fragile watermarking scheme leveraging a 64-bit complex-float computational pipeline and the Discrete Orthonormal Stockwell Transform (DOST) combined with Local Binary Pattern (LBP) features. To improve security, the architecture incorporates Arnold scrambling for confusion and chaotic mapping for diffusion-based encryption. In the attack-free scenario, the proposed method significantly outperformed the recent state-of-the-art (SOTA) technique across two MRI datasets. On the TCIA MRI dataset, the proposed method achieved mean values of 79.84 dB for Peak Signal-to-Noise Ratio (PSNR) and 0.9969 for Structural Similarity Index (SSIM), representing average improvements of 111.61% and 4.35%, respectively, over the SOTA baseline (37.73 dB and 0.9553). On the BMIBTD MRI dataset, the proposed method achieved mean values of 65.62 dB for PSNR and 0.9827 for SSIM, reflecting average improvements of 77.54% in PSNR and 11.14% in SSIM compared to the SOTA values (36.96 dB and 0.8842). Furthermore, the proposed method maintained a mean Normalised Correlation Coefficient (NCC) of 1, demonstrating performance parity with the SOTA technique in terms of general robustness. Extensive robustness analysis reveals a selective, tiered sensitivity profile: the scheme exhibits high resilience to stochastic channel noise (NCC$\gtrsim$0.90), a characteristic semi-fragile response to Median filtering (NCC$\approx$0.6–0.8), and high sensitivity to deterministic manipulations such as JPEG compression, Mean filtering, and geometric transformations. Rather than a limitation, this tiered behaviour serves as an inherent integrity-authentication mechanism, where the degradation or destruction of the mantissa-embedded watermark provides immediate detection of unauthorised spatial tampering or resampling post-acquisition. Computational time analysis demonstrates that the proposed method maintains an efficient total mean execution time of 0.2203 seconds, with separate mean processing times of 0.1293 seconds for embedding and 0.0910 seconds for extraction, ensuring rapid performance for real-time clinical applications. The capacity (8.00 bpp) and efficiency (η = 0.0625) of the proposed technique were analysed and found to be high compared to recent SOTA methods. Security analysis confirms the proposed framework's robustness against statistical, differential, and brute-force attacks, achieving superior performance metrics—including high NPCR and information entropy—compared to recent SOTA methods. Beyond MRI datasets, the system demonstrated comparable performance when validated on X-ray and ultrasound scans, confirming the framework's cross-modality consistency. The proposed system provides a significant advancement in balancing high-fidelity diagnostic reconstruction with robust defence against cryptanalytic threats.

1 Supplementary Data

.

Table S1. Comparison of Robustness Metrics on Original vs. Extracted Watermark Under Various Non-geometric Attacks on the BMIBTD dataset (Values: Mean / Std.). The subscript $_{ae}$ indicates the attack after scrambling and encryption, while the subscript $_{be}$ indicates the attack before scrambling and encryption. Chaudhary's method does not scramble or encrypt the watermarked image.

Attack Type	Method	NCC	BER	$NPCR_W$	$UACI_W$
Attack Free	Vaidya[1]	1.0000 / 0	0 / 0	0 / 0	0 / 0
	Chaudhary[2]	0.9087 / 0.1704	0.086 / 0.078	24.12 / 20.82	2.691 / 6.322
	Proposed	1.0000 / 0	0 / 0	0 / 0	0 / 0
Gaussian Noise (μ = 0, σ^2 = 0.002)	$Vaidya_{ae}$	0.9995 / 0.0004	0.058 / 0.010	37.06 / 4.44	0.211 / 0.070
	$Vaidya_{be}$	0.9995 / 0.0004	0.058 / 0.010	37.08 / 4.42	0.211 / 0.069
	Chaudhary	0.9087 / 0.1704	0.086 / 0.078	24.12 / 20.82	2.691 / 6.322
	$Proposed_{ae}$	0.8821 / 0.0031	0.174 / 0.001	54.27 / 0.11	3.611 / 0.054
	$Proposed_{be}$	0.8820 / 0.0030	0.174 / 0.001	54.27 / 0.11	3.611 / 0.054
Gaussian Noise (μ = 0.01, σ^2 = 0.002)	$Vaidya_{ae}$	0.9997 / 0.0002	0.038 / 0.007	22.65 / 3.28	0.124 / 0.041
	$Vaidya_{be}$	0.9997 / 0.0002	0.038 / 0.007	22.69 / 3.26	0.124 / 0.041
	Chaudhary	0.9087 / 0.1704	0.086 / 0.078	24.12 / 20.82	2.691 / 6.322
	$Proposed_{ae}$	0.8942 / 0.0029	0.146 / 0.001	46.38 / 0.11	2.877 / 0.044
	$Proposed_{be}$	0.8941 / 0.0029	0.146 / 0.001	46.38 / 0.10	2.877 / 0.043
Salt and Pepper (Noise density = 0.001)	$Vaidya_{ae}$	0.7111 / 0.0364	0.024 / 0.003	6.09 / 0.65	1.680 / 0.256
	$Vaidya_{be}$	0.7112 / 0.0351	0.024 / 0.003	6.07 / 0.67	1.675 / 0.255
	Chaudhary	0.7080 / 0.2073	0.124 / 0.073	31.33 / 15.03	5.790 / 6.567
	$Proposed_{ae}$	0.9995 / 0.0002	4×10^{-5} / 9×10^{-6}	0.01 / 2×10^{-3}	0.003 / 7×10^{-4}
	$Proposed_{be}$	0.9966 / 0.0037	1×10^{-4} / 8×10^{-5}	0.02 / 8×10^{-3}	0.009 / 0.008
Salt and Pepper (Noise density = 0.002)	$Vaidya_{ae}$	0.5629 / 0.0384	0.045 / 0.005	11.15 / 1.08	3.139 / 0.435
	$Vaidya_{be}$	0.5637 / 0.0384	0.045 / 0.005	11.14 / 1.07	3.131 / 0.432
	Chaudhary	0.5377 / 0.1875	0.153 / 0.072	35.52 / 12.82	9.537 / 7.389
	$Proposed_{ae}$	0.9989 / 2×10^{-4}	8×10^{-5} / 1×10^{-5}	0.02 / 3×10^{-3}	0.006 / 0.001
	$Proposed_{be}$	0.9933 / 0.0071	2×10^{-4} / 2×10^{-4}	0.03 / 0.02	0.018 / 0.016
JPEG Compression (Quality = 80 %)	$Vaidya_{ae}$	0.1200 / 0.0397	0.234 / 0.028	49.16 / 5.82	20.450 / 2.516
	$Vaidya_{be}$	0.6497 / 0.0973	0.197 / 0.040	54.17 / 10.92	6.930 / 1.774
	Chaudhary	0.8200 / 0.3312	0.099 / 0.082	27.37 / 21.11	3.515 / 6.903
	$Proposed_{ae}$	0 / 0	0.042 / 0	10.48 / 0	2.773 / 3×10^{-15}
	$Proposed_{be}$	-6×10^{-5} / 0.1087	0.240 / 0.089	40.46 / 11.74	27.001 / 11.186
Median Filter (3×3 window)	$Vaidya_{ae}$	0.0719 / 0.0333	0.122 / 0.022	28.26 / 4.68	9.336 / 2.022
	$Vaidya_{be}$	0.5319 / 0.1173	0.165 / 0.021	46.26 / 7.32	6.838 / 1.811
	Chaudhary	-0.1931 / 0.1147	0.332 / 0.125	62.97 / 14.27	27.770 / 14.507
	$Proposed_{ae}$	0.0828 / 0.0055	0.042 / 3×10^{-5}	10.38 / 7×10^{-3}	2.753 / 0.003
	$Proposed_{be}$	0.7820 / 0.0684	0.036 / 0.009	12.34 / 2.57	1.225 / 0.370
Median Filter (5×5 window)	$Vaidya_{ae}$	0.0340 / 0.0296	0.100 / 0.020	22.53 / 3.54	7.975 / 2.132
	$Vaidya_{be}$	0.2792 / 0.0632	0.188 / 0.031	45.77 / 8.20	11.859 / 2.141
	Chaudhary	-0.3152 / 0.1202	0.530 / 0.123	72.15 / 11.02	51.497 / 13.070
	$Proposed_{ae}$	0.0257 / 0.0052	0.042 / 2×10^{-5}	10.48 / 3×10^{-3}	2.774 / 0.002
	$Proposed_{be}$	0.5696 / 0.1003	0.050 / 0.010	16.58 / 2.82	2.047 / 0.434
Mean Filter (3×3 window)	$Vaidya_{ae}$	0.0188 / 0.0263	0.080 / 0.015	18.11 / 2.77	6.356 / 1.591
	$Vaidya_{be}$	0.3845 / 0.0810	0.176 / 0.020	47.09 / 6.30	8.957 / 1.662
	Chaudhary	-0.2638 / 0.0553	0.444 / 0.073	66.56 / 7.17	42.106 / 8.083
	$Proposed_{ae}$	-2×10^{-4} / 0.0016	0.042 / 3×10^{-6}	10.48 / 5×10^{-4}	2.774 / 2×10^{-4}
	$Proposed_{be}$	0.0735 / 0.0801	0.130 / 0.031	28.03 / 5.66	12.237 / 3.329
Mean Filter (5×5 window)	$Vaidya_{ae}$	0.0225 / 0.0240	0.071 / 0.018	16.09 / 3.35	5.503 / 1.774
	$Vaidya_{be}$	0.2251 / 0.0528	0.182 / 0.031	43.83 / 7.47	12.190 / 2.334
	Chaudhary	-0.3233 / 0.0530	0.568 / 0.057	70.30 / 5.98	55.440 / 5.860
	$Proposed_{ae}$	2×10^{-4} / 0.0021	0.042 / 2×10^{-6}	10.48 / 4×10^{-4}	2.774 / 2×10^{-4}
	$Proposed_{be}$	0.0455 / 0.0913	0.108 / 0.027	23.16 / 5.06	9.275 / 2.839
Histogram Equalisation	$Vaidya_{ae}$	0.0560 / 0.0991	0.051 / 0.013	12.22 / 2.49	3.455 / 1.329
	$Vaidya_{be}$	0.0560 / 0.0991	0.051 / 0.013	12.22 / 2.49	3.455 / 1.329
	Chaudhary	0.1509 / 0.2638	0.059 / 0.093	12.79 / 9.16	3.983 / 9.503
	$Proposed_{ae}$	0.0050 / 0.0087	0.043 / 5×10^{-4}	10.67 / 0.10	2.833 / 0.040
	$Proposed_{be}$	0.0002 / 0.0370	0.044 / 0.005	10.88 / 0.99	2.962 / 0.474

Table S2. Comparison of Robustness Metrics on Original vs. Extracted Watermark Under Various Geometric Attacks on the BMIBTD dataset (Values: Mean / Std.). The subscript $_{ae}$ indicates the attack after scrambling and encryption, while the subscript $_{be}$ indicates the attack before scrambling and encryption. Chaudhary's method does not scramble or encrypt the watermarked image.

Attack Type	Method	NCC	BER	$NPCR_W$	$UACI_W$
Attack Free	Vaidya[1]	1.0000 / 0	0 / 0	0 / 0	0 / 0
	Chaudhary[2]	0.9087 / 0.1704	0.086 / 0.078	24.12 / 20.82	2.691 / 6.322
	Proposed	1.0000 / 0	0 / 0	0 / 0	0 / 0
Scaling (2× followed by 0.5×)	$Vaidya_{ae}$	-0.0147 / 0.0439	0.155 / 0.027	32.33 / 5.12	14.150 / 2.750
	$Vaidya_{be}$	0.9466 / 0.0730	0.099 / 0.012	42.87 / 5.23	1.226 / 0.741
	Chaudhary	0.6998 / 0.3620	0.102 / 0.087	26.99 / 19.97	4.038 / 8.010
	$Proposed_{ae}$	0.0001 / 0.0019	$0.042 / 6 \times 10^{-6}$	$10.49 / 1 \times 10^{-3}$	$2.775 / 5 \times 10^{-4}$
	$Proposed_{be}$	0.2771 / 0.0746	0.192 / 0.035	44.09 / 8.14	13.476 / 2.837
Rotation (θ = 1°)	$Vaidya_{ae}$	0.0163 / 0.0147	0.065 / 0.010	15.01 / 2.01	4.955 / 1.046
	$Vaidya_{be}$	0.1123 / 0.0489	0.182 / 0.035	40.63 / 7.42	14.498 / 3.039
	Chaudhary	0.1416 / 0.1439	0.358 / 0.148	56.23 / 11.45	33.746 / 16.627
	$Proposed_{ae}$	-0.0004 / 0.0012	$0.042 / 4 \times 10^{-6}$	$10.48 / 7 \times 10^{-4}$	$2.774 / 3 \times 10^{-4}$
	$Proposed_{be}$	0.0442 / 0.0867	0.081 / 0.012	18.97 / 2.88	6.047 / 1.028
Rotation (θ = 2°)	$Vaidya_{ae}$	0.0198 / 0.0167	0.064 / 0.013	14.85 / 2.54	4.872 / 1.325
	$Vaidya_{be}$	0.0591 / 0.0410	0.170 / 0.032	37.53 / 6.83	13.947 / 2.935
	Chaudhary	0.1162 / 0.1216	0.413 / 0.146	58.31 / 11.61	40.462 / 15.873
	$Proposed_{ae}$	2×10^{-5} / 0.0019	$0.042 / 3 \times 10^{-6}$	$10.48 / 6 \times 10^{-4}$	$2.774 / 3 \times 10^{-4}$
	$Proposed_{be}$	0.0196 / 0.0655	0.077 / 0.011	17.84 / 2.59	5.659 / 0.957
Rotation (θ = 5°)	$Vaidya_{ae}$	0.0167 / 0.0146	0.065 / 0.010	14.99 / 1.93	4.943 / 1.011
	$Vaidya_{be}$	0.0083 / 0.0264	0.163 / 0.027	34.95 / 5.69	13.632 / 2.573
	Chaudhary	0.1222 / 0.1107	0.462 / 0.129	59.81 / 10.60	46.193 / 13.731
	$Proposed_{ae}$	2×10^{-6} / 0.0020	$0.042 / 2 \times 10^{-6}$	$10.48 / 5 \times 10^{-4}$	$2.774 / 2 \times 10^{-4}$
	$Proposed_{be}$	-0.0110 / 0.0358	0.079 / 0.017	17.61 / 2.69	5.996 / 1.609
Rotation (θ = 90°)	$Vaidya_{ae}$	0.0169 / 0.0153	0.065 / 0.011	14.94 / 2.14	4.921 / 1.118
	$Vaidya_{be}$	-0.0120 / 0.0335	0.116 / 0.026	26.66 / 5.26	9.066 / 2.505
	Chaudhary	0.9087 / 0.1704	0.086 / 0.078	24.20 / 20.82	2.691 / 6.322
	$Proposed_{ae}$	-0.0004 / 0.0012	$0.042 / 4 \times 10^{-6}$	$10.48 / 8 \times 10^{-4}$	$2.774 / 4 \times 10^{-4}$
	$Proposed_{be}$	-0.0080 / 0.0119	0.050 / 0.006	14.12 / 2.20	2.923 / 0.189

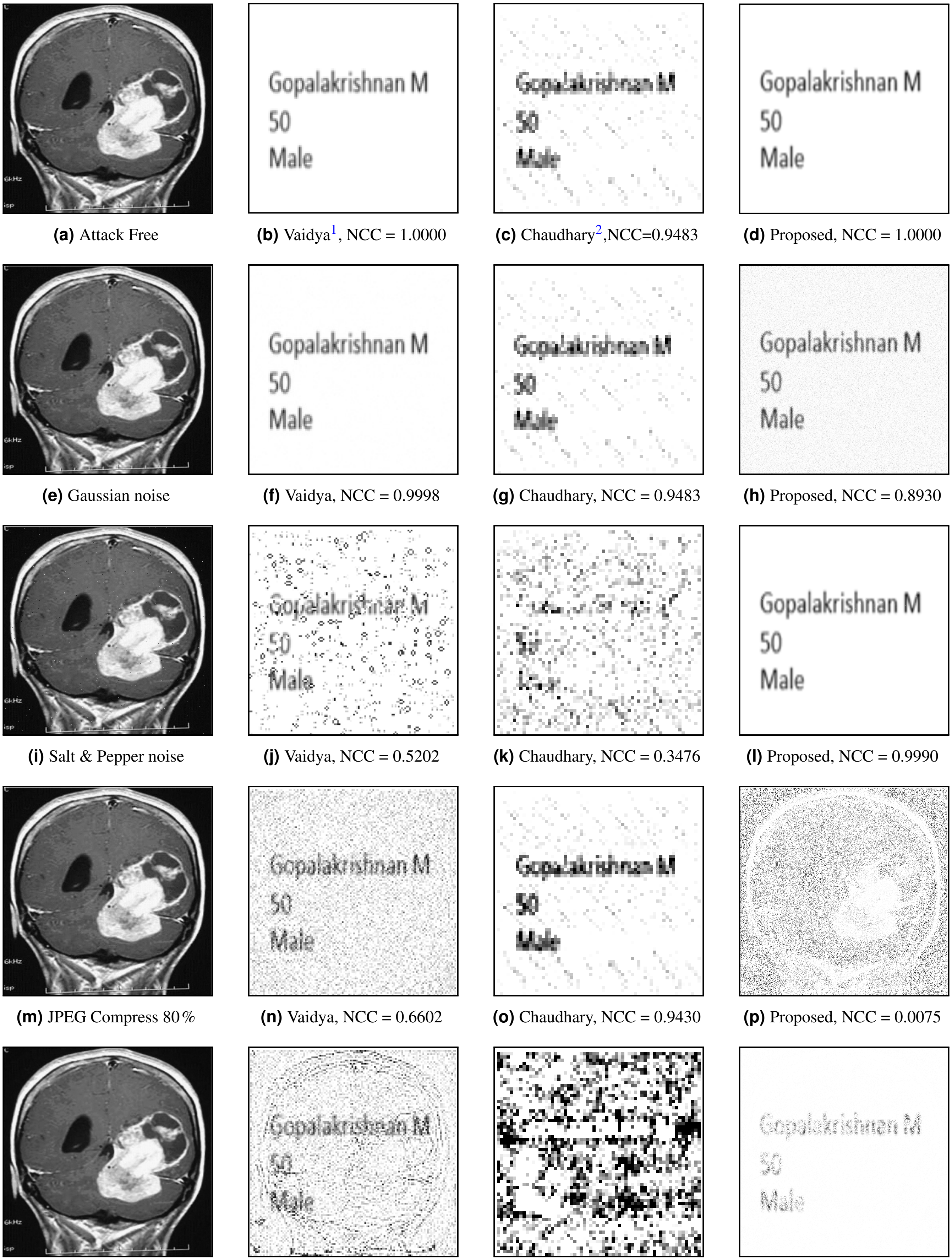

(a) Attack Free
(b) Vaidya[1], NCC = 1.0000
(c) Chaudhary[2],NCC=0.9483
(d) Proposed, NCC = 1.0000
(e) Gaussian noise
(f) Vaidya, NCC = 0.9998
(g) Chaudhary, NCC = 0.9483
(h) Proposed, NCC = 0.8930
(i) Salt & Pepper noise
(j) Vaidya, NCC = 0.5202
(k) Chaudhary, NCC = 0.3476
(l) Proposed, NCC = 0.9990
(m) JPEG Compress 80%
(n) Vaidya, NCC = 0.6602
(o) Chaudhary, NCC = 0.9430
(p) Proposed, NCC = 0.0075
(q) Median Filter (3×3)
(r) Vaidya, NCC = 0.4734
(s) Chaudhary, NCC = -0.1945
(t) Proposed, NCC = 0.6072

Figure S1. Visual comparison of extracted watermarks using the BMIBTD dataset. First column represents the watermarked host images after the respective attack. Gaussian noise parameters are $\mu = 0.01$, $\sigma^2 = 0.002$, Salt and Pepper noise parameter is noise density = 0.002. Refer to the caption of Fig. S2 for technical details regarding watermark resolutions.

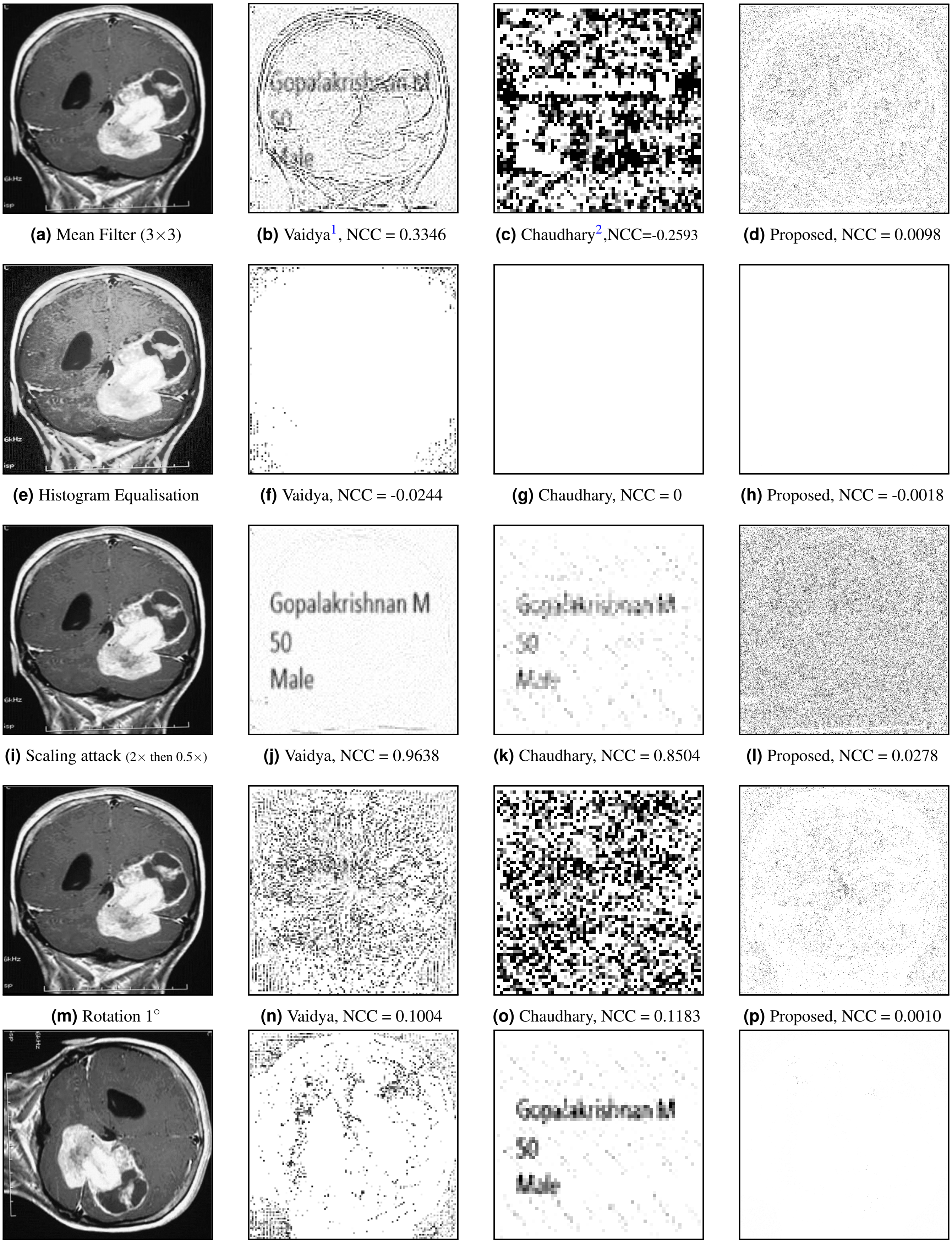

(a) Mean Filter (3×3) **(b)** Vaidya[1], NCC = 0.3346 **(c)** Chaudhary[2],NCC=-0.2593 **(d)** Proposed, NCC = 0.0098

(e) Histogram Equalisation **(f)** Vaidya, NCC = -0.0244 **(g)** Chaudhary, NCC = 0 **(h)** Proposed, NCC = -0.0018

(i) Scaling attack (2× then 0.5×) **(j)** Vaidya, NCC = 0.9638 **(k)** Chaudhary, NCC = 0.8504 **(l)** Proposed, NCC = 0.0278

(m) Rotation 1° **(n)** Vaidya, NCC = 0.1004 **(o)** Chaudhary, NCC = 0.1183 **(p)** Proposed, NCC = 0.0010

(q) Rotation 90° **(r)** Vaidya, NCC = 0.0058 **(s)** Chaudhary, NCC = 0.9483 **(t)** Proposed, NCC = -0.0092

Figure S2. Visual comparison of extracted watermarks using the BMIBTD dataset. First column represents the watermarked host images after the respective attack. For the proposed method, the extraction utilises the native 512×512 watermark resolution, while Vaidya's[1] and Chaudhary's[2] extractions correspond to their native 128×128 and 64×64 dimensions, respectively. NCC values are calculated on these native dimensions without interpolation to prevent artificial smoothing bias.

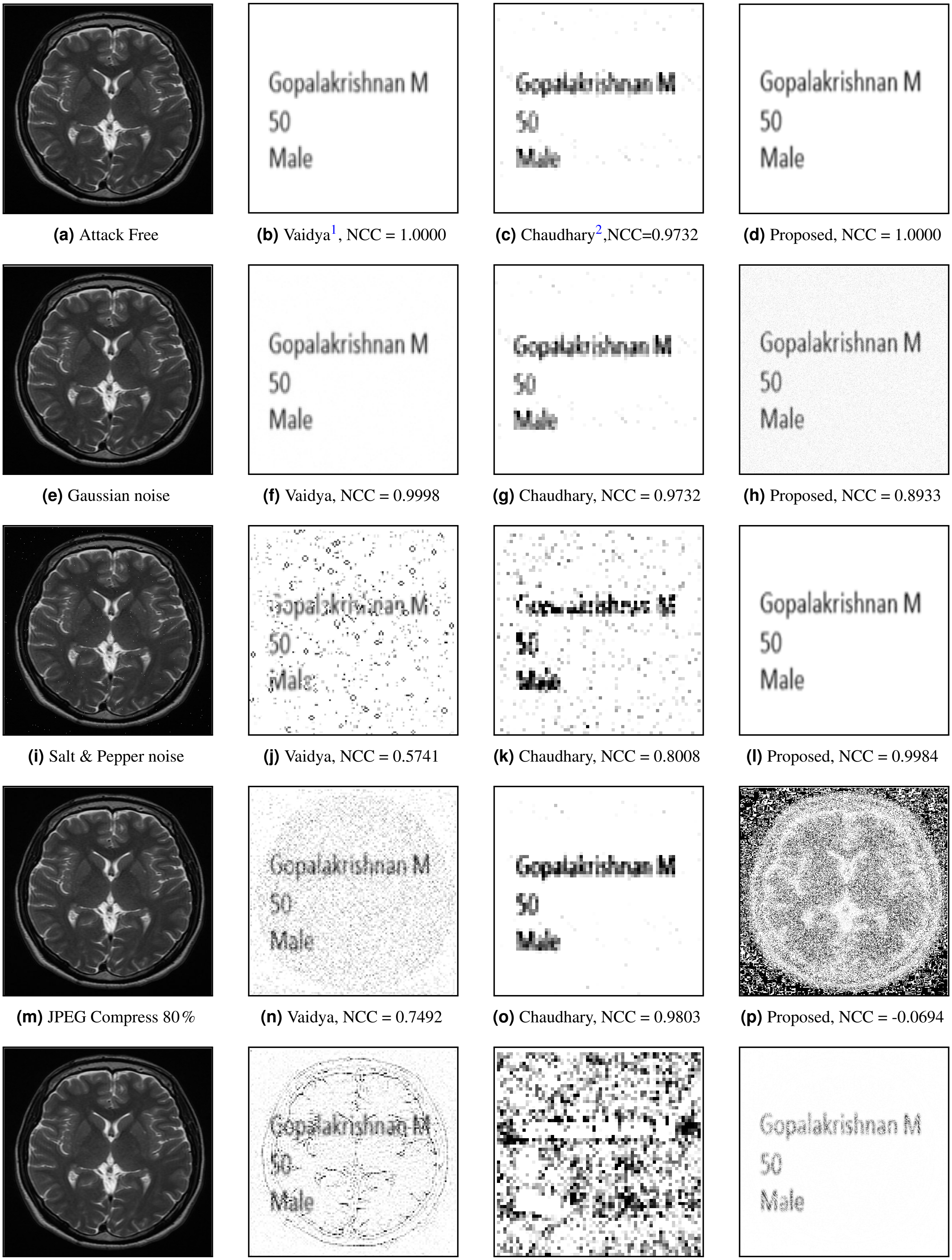

(a) Attack Free

(b) Vaidya[1], NCC = 1.0000

(c) Chaudhary[2],NCC=0.9732

(d) Proposed, NCC = 1.0000

(e) Gaussian noise

(f) Vaidya, NCC = 0.9998

(g) Chaudhary, NCC = 0.9732

(h) Proposed, NCC = 0.8933

(i) Salt & Pepper noise

(j) Vaidya, NCC = 0.5741

(k) Chaudhary, NCC = 0.8008

(l) Proposed, NCC = 0.9984

(m) JPEG Compress 80%

(n) Vaidya, NCC = 0.7492

(o) Chaudhary, NCC = 0.9803

(p) Proposed, NCC = -0.0694

(q) Median Filter (3×3)

(r) Vaidya, NCC = 0.5939

(s) Chaudhary, NCC = -0.2073

(t) Proposed, NCC = 0.7770

Figure S3. Visual comparison of extracted watermarks using the BMIBTD dataset. First column represents the watermarked host images after the respective attack. Gaussian noise parameters are $\mu = 0.01$, $\sigma^2 = 0.002$, Salt and Pepper noise parameter is noise density = 0.002. Refer to the caption of Fig. S4 for technical details regarding watermark resolutions.

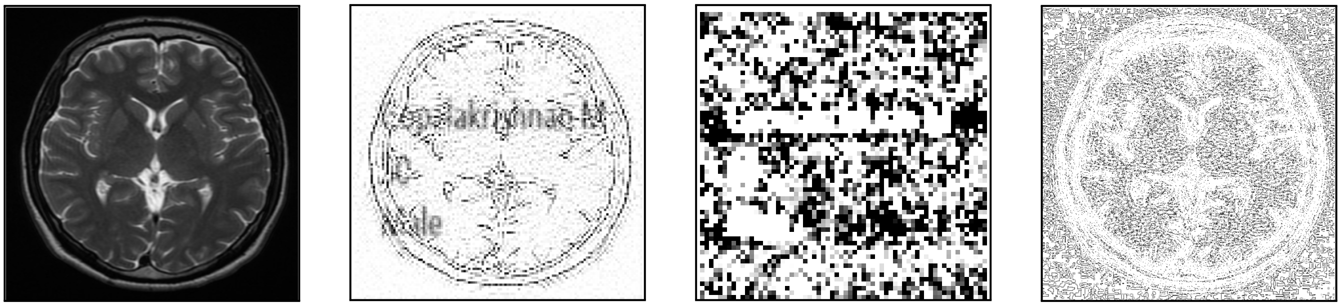

(a) Mean Filter (3×3) **(b)** Vaidya[1], NCC = 0.3939 **(c)** Chaudhary[2],NCC=-0.2434 **(d)** Proposed, NCC = -0.0131

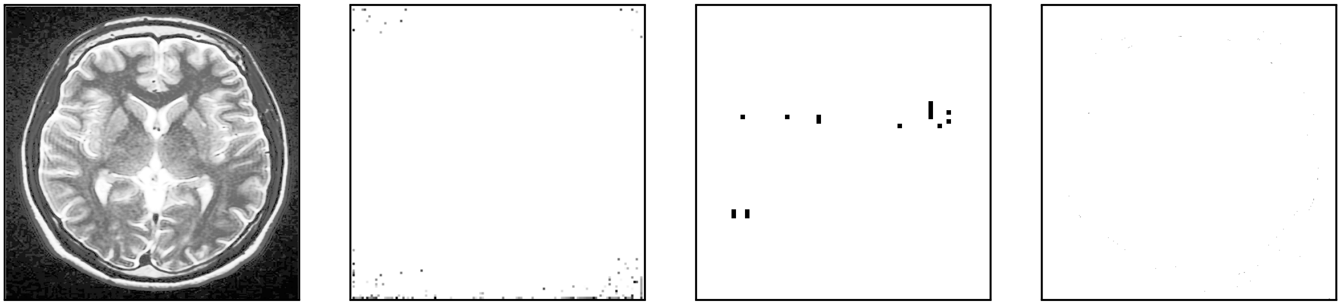

(e) Histogram Equalisation **(f)** Vaidya, NCC = -0.0210 **(g)** Chaudhary, NCC = 0.3477 **(h)** Proposed, NCC = -0.0005

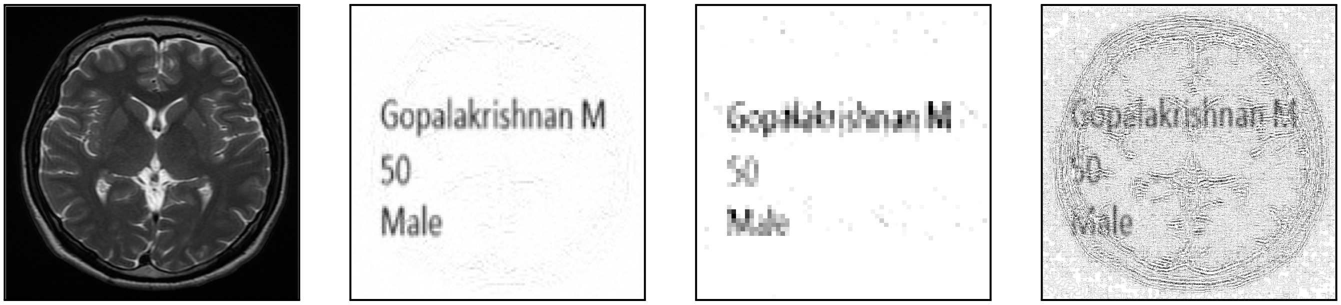

(i) Scaling attack (2× then 0.5×) **(j)** Vaidya, NCC = 0.9892 **(k)** Chaudhary, NCC = 0.9374 **(l)** Proposed, NCC = 0.2607

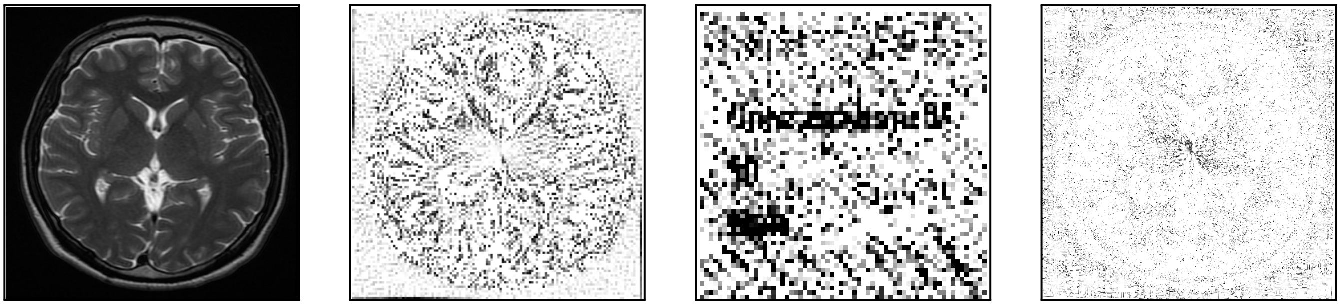

(m) Rotation 1° **(n)** Vaidya, NCC = 0.0943 **(o)** Chaudhary, NCC = 0.3931 **(p)** Proposed, NCC = -0.0267

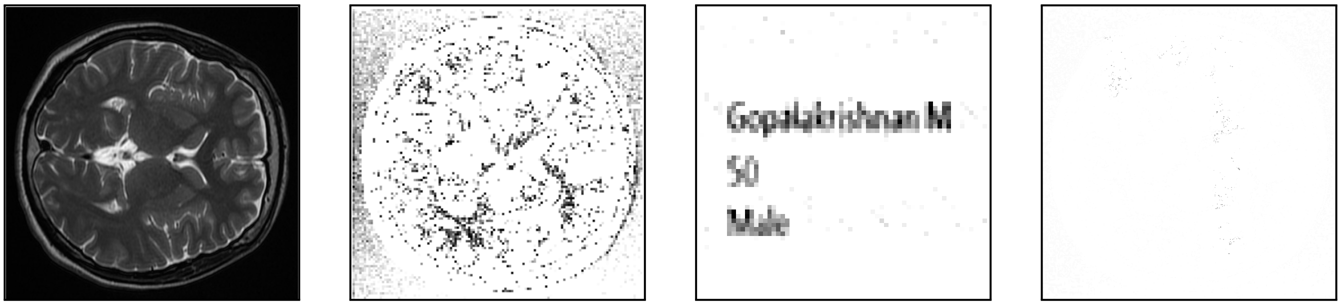

(q) Rotation 90° **(r)** Vaidya, NCC = -0.0180 **(s)** Chaudhary, NCC = 0.9732 **(t)** Proposed, NCC = -0.0164

Figure S4. Visual comparison of extracted watermarks using the BMIBTD dataset. First column represents the watermarked host images after the respective attack. For the proposed method, the extraction utilises the native 512×512 watermark resolution, while Vaidya's[1] and Chaudhary's[2] extractions correspond to their native 128×128 and 64×64 dimensions, respectively. NCC values are calculated on these native dimensions without interpolation to prevent artificial smoothing bias.

Table S3. Comparison of Robustness Metrics on Original vs. Extracted Watermark Under Various Non-geometric Attacks on the CXIP dataset (Values: Mean / Std.). The subscript $_{ae}$ indicates the attack after scrambling and encryption, while the subscript $_{be}$ indicates the attack before scrambling and encryption. Chaudhary's method does not scramble or encrypt the watermarked image.

Attack Type	Method	NCC	BER	$\mathbf{NPCR}_W$	$\mathbf{UACI}_W$
Attack Free	Vaidya[1]	1.0000 / 0	0 / 0	0 / 0	0 / 0
	Chaudhary[2]	0.9091 / 0.0901	0.051 / 0.008	12.71 / 2.47	1.662 / 0.823
	Proposed	1.0000 / 0	0 / 0	0 / 0	0 / 0
Gaussian Noise ($\mu = 0$, $\sigma^2 = 0.002$)	Vaidya_{ae}	$0.9997 / 5 \times 10^{-5}$	0.052 / 0.003	34.60 / 1.60	0.170 / 0.016
	Vaidya_{be}	$0.9997 / 5 \times 10^{-5}$	0.052 / 0.003	34.57 / 1.54	0.170 / 0.016
	Chaudhary	0.9091 / 0.0901	0.051 / 0.008	12.71 / 2.47	1.662 / 0.823
	Proposed_{ae}	0.8811 / 0.0005	$0.174 / 3 \times 10^{-4}$	54.24 / 0.09	3.625 / 0.011
	Proposed_{be}	0.8810 / 0.0005	$0.174 / 3 \times 10^{-4}$	54.26 / 0.10	3.627 / 0.012
Gaussian Noise ($\mu = 0.01$, $\sigma^2 = 0.002$)	Vaidya_{ae}	$0.9998 / 3 \times 10^{-5}$	0.034 / 0.002	20.79 / 1.07	0.100 / 0.010
	Vaidya_{be}	$0.9998 / 4 \times 10^{-5}$	0.034 / 0.002	20.82 / 1.06	0.100 / 0.010
	Chaudhary	0.9091 / 0.0901	0.051 / 0.008	12.71 / 2.47	1.662 / 0.823
	Proposed_{ae}	0.8933 / 0.0005	$0.147 / 4 \times 10^{-4}$	46.35 / 0.09	2.889 / 0.009
	Proposed_{be}	0.8931 / 0.0005	$0.147 / 4 \times 10^{-4}$	46.37 / 0.09	2.891 / 0.010
Salt and Pepper (Noise density = 0.001)	Vaidya_{ae}	0.7043 / 0.0233	0.026 / 0.002	6.20 / 0.46	1.794 / 0.184
	Vaidya_{be}	0.6983 / 0.0189	0.026 / 0.002	6.24 / 0.43	1.873 / 0.158
	Chaudhary	0.7991 / 0.0639	0.112 / 0.019	27.72 / 4.76	6.188 / 1.129
	Proposed_{ae}	0.9995 / 0.0001	$4 \times 10^{-5} / 8 \times 10^{-6}$	$0.01 / 2 \times 10^{-3}$	$0.003 / 6 \times 10^{-4}$
	Proposed_{be}	0.9980 / 0.0016	$7 \times 10^{-5} / 4 \times 10^{-5}$	$0.01 / 4 \times 10^{-3}$	0.006 / 0.004
Salt and Pepper (Noise density = 0.002)	Vaidya_{ae}	0.5514 / 0.0304	0.048 / 0.003	11.48 / 0.78	3.408 / 0.311
	Vaidya_{be}	0.5488 / 0.0272	0.048 / 0.003	11.48 / 0.73	3.423 / 0.280
	Chaudhary	0.5807 / 0.0787	0.182 / 0.020	39.19 / 3.10	14.362 / 2.641
	Proposed_{ae}	$0.9990 / 2 \times 10^{-4}$	$8 \times 10^{-5} / 1 \times 10^{-5}$	$0.02 / 3 \times 10^{-3}$	0.005 / 0.001
	Proposed_{be}	0.9961 / 0.0029	$1 \times 10^{-4} / 7 \times 10^{-5}$	$0.03 / 8 \times 10^{-3}$	0.012 / 0.007
JPEG Compression (Quality = 80 %)	Vaidya_{ae}	0.1303 / 0.0177	0.255 / 0.007	53.41 / 1.58	22.391 / 0.714
	Vaidya_{be}	0.6533 / 0.0304	0.199 / 0.011	53.76 / 3.21	7.009 / 0.553
	Chaudhary	0.8653 / 0.1272	0.053 / 0.011	13.13 / 3.17	1.865 / 0.803
	Proposed_{ae}	0 / 0	0.042 / 0	10.48 / 0	2.773 / 0
	Proposed_{be}	-0.0158 / 0.0286	0.158 / 0.021	31.95 / 3.67	13.539 / 2.927
Median Filter (3×3 window)	Vaidya_{ae}	0.0179 / 0.0303	0.135 / 0.016	29.89 / 3.25	11.050 / 1.569
	Vaidya_{be}	0.6248 / 0.0531	0.185 / 0.010	52.57 / 2.32	6.316 / 1.044
	Chaudhary	-0.0929 / 0.0685	0.217 / 0.060	44.94 / 9.89	16.710 / 6.136
	Proposed_{ae}	0.0820 / 0.0043	$0.042 / 3 \times 10^{-5}$	$10.38 / 5 \times 10^{-3}$	2.753 / 0.002
	Proposed_{be}	0.6248 / 0.0377	0.051 / 0.008	19.43 / 1.53	1.816 / 0.125
Median Filter (5×5 window)	Vaidya_{ae}	0.0262 / 0.0194	0.093 / 0.013	20.45 / 2.49	7.629 / 1.258
	Vaidya_{be}	0.3693 / 0.0546	0.209 / 0.010	53.14 / 2.39	10.955 / 1.667
	Chaudhary	-0.2273 / 0.0615	0.396 / 0.097	60.69 / 9.12	37.124 / 10.183
	Proposed_{ae}	0.0252 / 0.0045	$0.042 / 1 \times 10^{-5}$	$10.47 / 2 \times 10^{-3}$	2.774 / 0.001
	Proposed_{be}	0.4767 / 0.0435	0.058 / 0.009	21.54 / 1.80	2.295 / 0.105
Mean Filter (3×3 window)	Vaidya_{ae}	0.0084 / 0.0200	0.084 / 0.012	18.73 / 2.36	6.844 / 1.233
	Vaidya_{be}	0.5331 / 0.0638	0.191 / 0.007	52.83 / 1.91	7.474 / 1.077
	Chaudhary	-0.1839 / 0.0247	0.305 / 0.045	53.52 / 4.62	27.190 / 4.848
	Proposed_{ae}	0.0012 / 0.0042	$0.042 / 3 \times 10^{-6}$	$10.48 / 7 \times 10^{-4}$	$2.774 / 3 \times 10^{-4}$
	Proposed_{be}	-0.0038 / 0.0148	0.137 / 0.017	29.74 / 3.40	12.849 / 1.843
Mean Filter (5×5 window)	Vaidya_{ae}	0.0400 / 0.0219	0.069 / 0.007	15.77 / 1.38	5.350 / 0.722
	Vaidya_{be}	0.2969 / 0.0490	0.214 / 0.008	52.58 / 2.47	12.534 / 1.396
	Chaudhary	-0.2475 / 0.0199	0.442 / 0.031	61.48 / 2.83	42.309 / 3.251
	Proposed_{ae}	0.0006 / 0.0030	$0.042 / 3 \times 10^{-6}$	$10.48 / 7 \times 10^{-4}$	$2.774 / 2 \times 10^{-4}$
	Proposed_{be}	-0.0119 / 0.0138	0.122 / 0.017	25.92 / 3.26	10.727 / 1.695
Histogram Equalisation	Vaidya_{ae}	-0.0154 / 0.0335	0.084 / 0.032	18.80 / 6.26	6.768 / 3.253
	Vaidya_{be}	-0.0154 / 0.0335	0.084 / 0.032	18.80 / 6.26	6.768 / 3.253
	Chaudhary	0.2680 / 0.2591	0.462 / 0..389	52.51 / 38.24	46.690 / 40.556
	Proposed_{ae}	0.0064 / 0.0023	$0.043 / 3 \times 10^{-4}$	10.75 / 0.05	2.865 / 0.022
	Proposed_{be}	-0.0068 / 0.0095	0.045 / 0.003	11.15 / 0.65	3.048 / 0.317

Table S4. Comparison of Robustness Metrics on Original vs. Extracted Watermark Under Various Geometric Attacks on the CXIP dataset (Values: Mean / Std.). The subscript $_{ae}$ indicates the attack after scrambling and encryption, while the subscript $_{be}$ indicates the attack before scrambling and encryption. Chaudhary's method does not scramble or encrypt the watermarked image.

Attack Type	Method	NCC	BER	NPCR_W	UACI_W
Attack Free	Vaidya[1]	1.0000 / 0	0 / 0	0 / 0	0 / 0
	Chaudhary[2]	0.9091 / 0.0901	0.051 / 0.008	12.71 / 2.47	1.662 / 0.823
	Proposed	1.0000 / 0	0 / 0	0 / 0	0 / 0
Scaling (2× followed by 0.5×)	Vaidya_{ae}	-0.0288 / 0.0186	0.151 / 0.018	31.86 / 3.47	13.594 / 1.964
	Vaidya_{be}	0.9447 / 0.0194	0.123 / 0.007	49.87 / 1.81	1.455 / 0.221
	Chaudhary	0.4165 / 0.3013	0.068 / 0.020	17.68 / 6.00	2.880 / 0.642
	Proposed_{ae}	0.0011 / 0.0022	$0.042 / 5 \times 10^{-6}$	$10.49 / 1 \times 10^{-3}$	$2.775 / 4 \times 10^{-4}$
	Proposed_{be}	0.0690 / 0.0257	0.230 / 0.014	48.09 / 3.13	20.708 / 1.238
Rotation (θ = 1°)	Vaidya_{ae}	0.0300 / 0.0147	0.068 / 0.006	15.47 / 1.13	5.211 / 0.568
	Vaidya_{be}	0.1002 / 0.0226	0.212 / 0.014	46.19 / 3.40	17.341 / 1.074
	Chaudhary	0.3090 / 0.0857	0.405 / 0.026	58.56 / 3.81	40.958 / 2.698
	Proposed_{ae}	0.0003 / 0.0020	$0.042 / 3 \times 10^{-6}$	$10.48 / 8 \times 10^{-4}$	$2.774 / 3 \times 10^{-4}$
	Proposed_{be}	-0.0008 / 0.0071	0.085 / 0.008	19.12 / 1.70	6.777 / 0.686
Rotation (θ = 2°)	Vaidya_{ae}	0.0327 / 0.0138	0.067 / 0.007	15.36 / 1.32	5.120 / 0.651
	Vaidya_{be}	0.0434 / 0.0129	0.191 / 0.018	40.87 / 3.83	16.484 / 1.480
	Chaudhary	0.2719 / 0.0666	0.484 / 0.035	61.47 / 4.14	49.781 / 3.650
	Proposed_{ae}	-0.0007 / 0.0003	$0.042 / 3 \times 10^{-6}$	$10.48 / 6 \times 10^{-4}$	$2.774 / 2 \times 10^{-4}$
	Proposed_{be}	-0.0062 / 0.0048	0.074 / 0.005	16.69 / 1.17	5.741 / 0.469
Rotation (θ = 5°)	Vaidya_{ae}	0.0191 / 0.0075	0.067 / 0.006	15.36 / 1.09	5.142 / 0.582
	Vaidya_{be}	-0.0023 / 0.0125	0.155 / 0.016	32.74 / 3.31	13.741 / 1.495
	Chaudhary	0.2318 / 0.0339	0.575 / 0.049	66.18 / 5.92	59.284 / 5.022
	Proposed_{ae}	-0.0004 / 0.0006	$0.042 / 3 \times 10^{-6}$	$10.48 / 8 \times 10^{-4}$	$2.774 / 2 \times 10^{-4}$
	Proposed_{be}	-0.0192 / 0.0114	0.067 / 0.006	14.77 / 0.75	5.161 / 0.642
Rotation (θ = 90°)	Vaidya_{ae}	0.0340 / 0.0163	0.068 / 0.006	15.51 / 1.19	5.219 / 0.609
	Vaidya_{be}	0.0156 / 0.0216	0.068 / 0.005	15.77 / 0.97	5.238 / 0.565
	Chaudhary	0.9091 / 0.0901	0.051 / 0.008	12.71 / 2.47	1.662 / 0.823
	Proposed_{ae}	-0.0005 / 0.0009	$0.042 / 2 \times 10^{-6}$	$10.48 / 5 \times 10^{-4}$	$2.774 / 2 \times 10^{-4}$
	Proposed_{be}	0.0007 / 0.0047	$0.043 / 4 \times 10^{-4}$	11.01 / 0.11	2.799 / 0.009

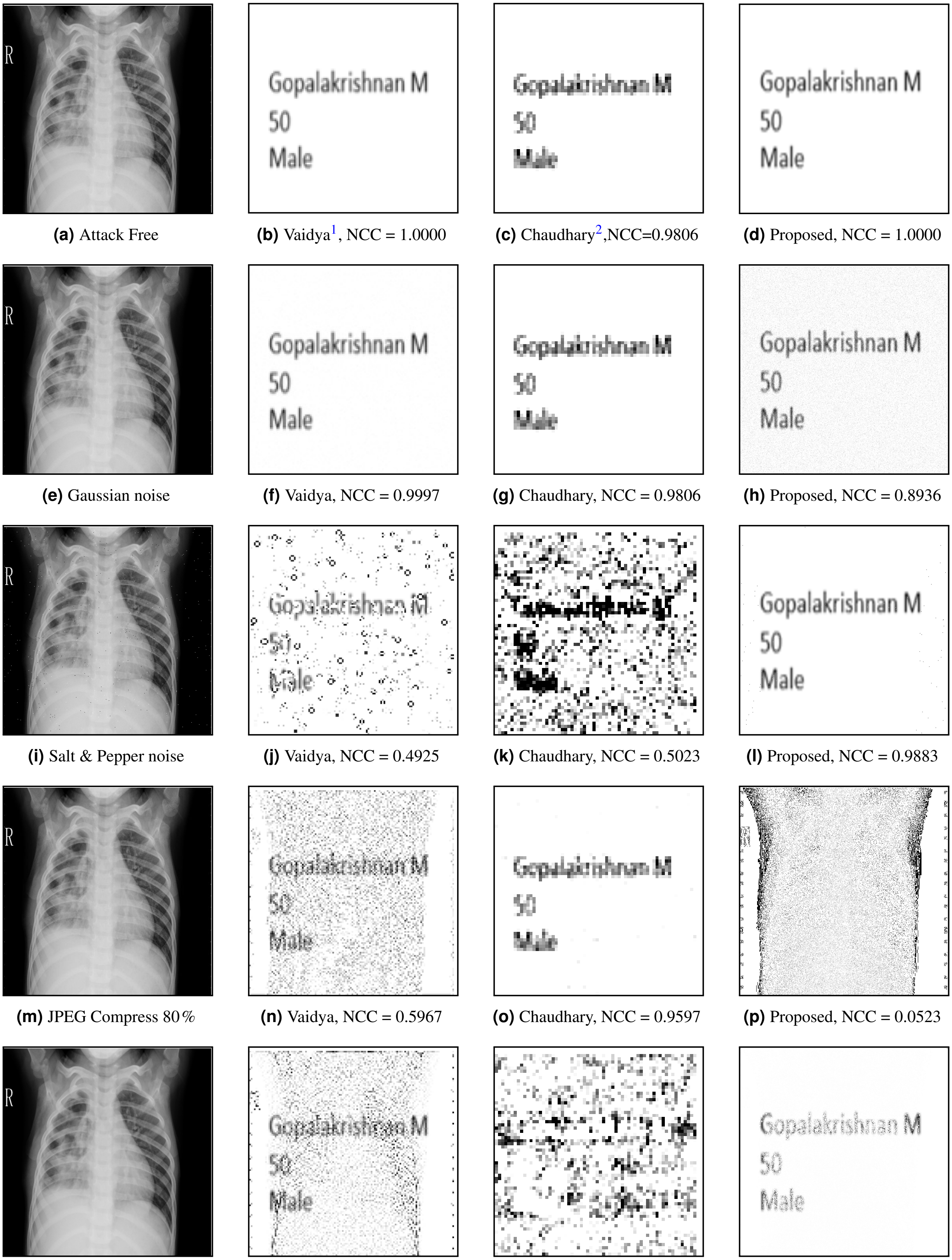

(a) Attack Free

(b) Vaidya[1], NCC = 1.0000

(c) Chaudhary[2],NCC=0.9806

(d) Proposed, NCC = 1.0000

(e) Gaussian noise

(f) Vaidya, NCC = 0.9997

(g) Chaudhary, NCC = 0.9806

(h) Proposed, NCC = 0.8936

(i) Salt & Pepper noise

(j) Vaidya, NCC = 0.4925

(k) Chaudhary, NCC = 0.5023

(l) Proposed, NCC = 0.9883

(m) JPEG Compress 80 %

(n) Vaidya, NCC = 0.5967

(o) Chaudhary, NCC = 0.9597

(p) Proposed, NCC = 0.0523

(q) Median Filter (3×3)

(r) Vaidya, NCC = 0.5836

(s) Chaudhary, NCC = -0.1039

(t) Proposed, NCC = 0.7096

Figure S5. Visual comparison of extracted watermarks using the CXIP dataset. First column represents the watermarked host images after the respective attack. Gaussian noise parameters are $\mu = 0.01$, $\sigma^2 = 0.002$, Salt and Pepper noise parameter is noise density = 0.002. Refer to the caption of Fig. S6 for technical details regarding watermark resolutions.

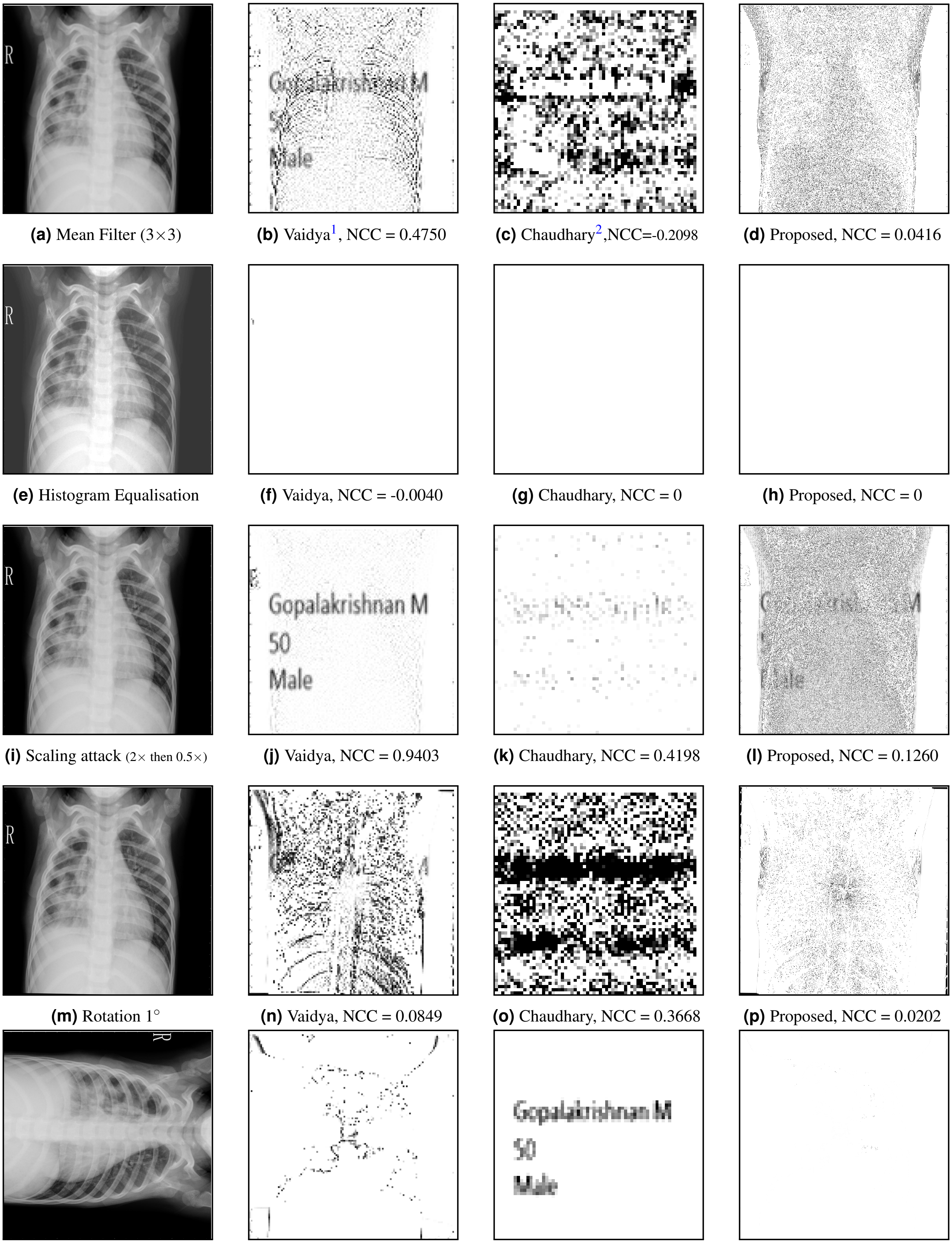

(a) Mean Filter (3×3) **(b)** Vaidya[1], NCC = 0.4750 **(c)** Chaudhary[2],NCC=-0.2098 **(d)** Proposed, NCC = 0.0416

(e) Histogram Equalisation **(f)** Vaidya, NCC = -0.0040 **(g)** Chaudhary, NCC = 0 **(h)** Proposed, NCC = 0

(i) Scaling attack (2× then 0.5×) **(j)** Vaidya, NCC = 0.9403 **(k)** Chaudhary, NCC = 0.4198 **(l)** Proposed, NCC = 0.1260

(m) Rotation 1° **(n)** Vaidya, NCC = 0.0849 **(o)** Chaudhary, NCC = 0.3668 **(p)** Proposed, NCC = 0.0202

(q) Rotation 90° **(r)** Vaidya, NCC = 0.0151 **(s)** Chaudhary, NCC = 0.9806 **(t)** Proposed, NCC = 0.0033

Figure S6. Visual comparison of extracted watermarks using the CXIP dataset. First column represents the watermarked host images after the respective attack. For the proposed method, the extraction utilises the native 512×512 watermark resolution, while Vaidya's[1] and Chaudhary's[2] extractions correspond to their native 128×128 and 64×64 dimensions, respectively. NCC values are calculated on these native dimensions without interpolation to prevent artificial smoothing bias.

Table S5. Comparison of Robustness Metrics on Original vs. Extracted Watermark Under Various Non-geometric Attacks on the LUI dataset (Values: Mean / Std.). The subscript $_{ae}$ indicates the attack after scrambling and encryption, while the subscript $_{be}$ indicates the attack before scrambling and encryption. Chaudhary's method does not scramble or encrypt the watermarked image.

**Attack Type**	**Method**	**NCC**	**BER**	**NPCR_W**	**UACI_W**
Attack Free	Vaidya[1]	1.0000 / 0	0 / 0	0 / 0	0 / 0
	Chaudhary[2]	0.8880 / 0.0796	0.236 / 0.092	71.33 / 22.53	5.547 / 3.441
	Proposed	1.0000 / 0	0 / 0	0 / 0	0 / 0
Gaussian Noise (μ = 0, σ^2 = 0.002)	Vaidya_{ae}	0.9995 / 0.0031	0.056 / 0.011	36.14 / 5.05	0.202 / 0.070
	Vaidya_{be}	0.9995 / 0.0031	0.056 / 0.011	36.16 / 5.10	0.202 / 0.071
	Chaudhary	0.8880 / 0.0796	0.236 / 0.092	71.33 / 22.53	5.547 / 3.441
	Proposed_{ae}	0.8812 / 0.0007	$0.174 / 4 \times 10^{-4}$	54.27 / 0.11	3.625 / 0.013
	Proposed_{be}	0.8811 / 0.0008	$0.174 / 4 \times 10^{-4}$	54.27 / 0.12	3.626 / 0.013
Gaussian Noise (μ = 0.01, σ^2 = 0.002)	Vaidya_{ae}	0.9997 / 0.0002	0.037 / 0.007	22.01 / 3.73	0.119 / 0.042
	Vaidya_{be}	0.9997 / 0.0002	0.037 / 0.007	22.00 / 3.72	0.119 / 0.042
	Chaudhary	0.8880 / 0.0796	0.236 / 0.092	71.33 / 22.53	5.547 / 3.441
	Proposed_{ae}	0.8934 / 0.0007	$0.147 / 3 \times 10^{-4}$	46.39 / 0.10	2.889 / 0.011
	Proposed_{be}	0.8933 / 0.0008	$0.147 / 4 \times 10^{-4}$	46.38 / 0.10	2.890 / 0.011
Salt and Pepper (Noise density = 0.001)	Vaidya_{ae}	0.7237 / 0.0300	0.025 / 0.003	6.17 / 0.67	1.639 / 0.229
	Vaidya_{be}	0.7223 / 0.0305	0.025 / 0.003	6.16 / 0.65	1.643 / 0.228
	Chaudhary	0.6671 / 0.1122	0.213 / 0.034	49.85 / 8.31	13.757 / 4.244
	Proposed_{ae}	0.9995 / 0.0002	$4 \times 10^{-5} / 8 \times 10^{-6}$	$0.01 / 2 \times 10^{-3}$	$0.003 / 7 \times 10^{-4}$
	Proposed_{be}	0.9949 / 0.0031	$1 \times 10^{-4} / 7 \times 10^{-5}$	$0.02 / 7 \times 10^{-3}$	0.013 / 0.007
Salt and Pepper (Noise density = 0.002)	Vaidya_{ae}	0.5747 / 0.0308	0.046 / 0.005	11.47 / 1.07	3.123 / 0.366
	Vaidya_{be}	0.5757 / 0.0330	0.046 / 0.005	11.42 / 1.06	3.119 / 0.379
	Chaudhary	0.5328 / 0.0945	0.228 / 0.034	46.25 / 4.70	19.078 / 4.984
	Proposed_{ae}	$0.9989 / 2 \times 10^{-4}$	$8 \times 10^{-5} / 1 \times 10^{-5}$	$0.02 / 3 \times 10^{-3}$	0.006 / 0.001
	Proposed_{be}	0.9898 / 0.0061	$3 \times 10^{-4} / 1 \times 10^{-4}$	0.04 / 0.01	0.026 / 0.014
JPEG Compression (Quality = 80 %)	Vaidya_{ae}	0.1241 / 0.0475	0.233 / 0.023	49.14 / 5.23	19.927 / 1.449
	Vaidya_{be}	0.6455 / 0.0988	0.198 / 0.036	54.88 / 9.55	6.979 / 2.002
	Chaudhary	0.7420 / 0.1565	0.233 / 0.080	68.55 / 19.45	6.137 / 3.104
	Proposed_{ae}	0 / 0	0.042 / 0	10.48 / 0	$2.773 / 4 \times 10^{-16}$
	Proposed_{be}	0.0537 / 0.0688	0.177 / 0.024	30.49 / 3.44	20.838 / 2.970
Median Filter (3×3 window)	Vaidya_{ae}	0.0812 / 0.0517	0.138 / 0.022	30.59 / 3.98	11.180 / 2.350
	Vaidya_{be}	0.4828 / 0.0676	0.183 / 0.024	47.53 / 6.11	8.496 / 1.597
	Chaudhary	-0.2240 / 0.0166	0.364 / 0.039	67.92 / 5.08	30.460 / 7.699
	Proposed_{ae}	0.0855 / 0.0047	$0.042 / 3 \times 10^{-5}$	$10.38 / 7 \times 10^{-3}$	2.751 / 0.003
	Proposed_{be}	0.7047 / 0.0605	0.040 / 0.005	14.44 / 1.23	1.499 / 0.210
Median Filter (5×5 window)	Vaidya_{ae}	0.0337 / 0.0326	0.114 / 0.018	24.90 / 3.34	9.487 / 1.850
	Vaidya_{be}	0.2197 / 0.0501	0.211 / 0.029	47.07 / 6.03	15.976 / 2.779
	Chaudhary	-0.2806 / 0.0190	0.491 / 0.035	69.89 / 5.40	47.058 / 4.153
	Proposed_{ae}	0.0267 / 0.0053	$0.042 / 2 \times 10^{-5}$	$10.47 / 3 \times 10^{-3}$	2.774 / 0.002
	Proposed_{be}	0.4737 / 0.1046	0.050 / 0.005	16.72 / 1.08	2.240 / 0.252
Mean Filter (3×3 window)	Vaidya_{ae}	0.0101 / 0.0292	0.108 / 0.018	23.39 / 3.43	9.153 / 1.845
	Vaidya_{be}	0.4041 / 0.0562	0.193 / 0.024	48.42 / 5.28	10.191 / 1.838
	Chaudhary	-0.2517 / 0.0279	0.426 / 0.064	68.29 / 2.49	39.377 / 7.925
	Proposed_{ae}	0.0007 / 0.0030	$0.042 / 2 \times 10^{-6}$	$10.48 / 5 \times 10^{-4}$	$2.774 / 2 \times 10^{-4}$
	Proposed_{be}	0.0556 / 0.0609	0.112 / 0.011	24.81 / 2.14	10.150 / 1.225
Mean Filter (5×5 window)	Vaidya_{ae}	0.0164 / 0.0320	0.086 / 0.012	19.14 / 2.40	7.053 / 1.245
	Vaidya_{be}	0.1974 / 0.0450	0.208 / 0.030	46.39 / 5.55	16.346 / 2.988
	Chaudhary	-0.2906 / 0.0230	0.518 / 0.045	68.72 / 2.35	50.170 / 4.735
	Proposed_{ae}	0.0004 / 0.0017	$0.042 / 2 \times 10^{-6}$	$10.48 / 4 \times 10^{-4}$	$2.774 / 2 \times 10^{-4}$
	Proposed_{be}	0.0484 / 0.0640	0.085 / 0.008	18.75 / 1.46	6.883 / 0.782
Histogram Equalisation	Vaidya_{ae}	0.1184 / 0.1611	0.044 / 0.001	11.01 / 0.23	2.833 / 0.159
	Vaidya_{be}	0.1184 / 0.1611	0.044 / 0.001	11.01 / 0.23	2.833 / 0.159
	Chaudhary	0.2447 / 0.1685	$0.048 / 3 \times 10^{-4}$	$11.72 / 3 \times 10^{-3}$	2.810 / 0.072
	Proposed_{ae}	0.0075 / 0.0085	$0.043 / 4 \times 10^{-4}$	10.66 / 0.09	2.830 / 0.030
	Proposed_{be}	0.1207 / 0.1796	$0.042 / 3 \times 10^{-4}$	$10.48 / 2 \times 10^{-4}$	2.787 / 0.047

Table S6. Comparison of Robustness Metrics on Original vs. Extracted Watermark Under Various Geometric Attacks on the LUI dataset (Values: Mean / Std.). The subscript $_{ae}$ indicates the attack after scrambling and encryption, while the subscript $_{be}$ indicates the attack before scrambling and encryption. Chaudhary's method does not scramble or encrypt the watermarked image.

Attack Type	Method	NCC	BER	$NPCR_W$	$UACI_W$
Attack Free	Vaidya[1]	1.0000 / 0	0 / 0	0 / 0	0 / 0
	Chaudhary[2]	0.8880 / 0.0796	0.236 / 0.092	71.33 / 22.53	5.547 / 3.441
	Proposed	1.0000 / 0	0 / 0	0 / 0	0 / 0
Scaling (2× followed by 0.5×)	$Vaidya_{ae}$	0.0264 / 0.0415	0.204 / 0.020	42.32 / 3.83	18.582 / 2.153
	$Vaidya_{be}$	0.9633 / 0.0203	0.112 / 0.015	44.96 / 5.69	1.330 / 0.337
	Chaudhary	0.3712 / 0.2940	0.223 / 0.078	63.73 / 18.58	6.975 / 3.406
	$Proposed_{ae}$	-0.0003 / 0.0014	$0.042 / 6 \times 10^{-6}$	$10.49 / 1 \times 10^{-3}$	$2.775 / 5 \times 10^{-4}$
	$Proposed_{be}$	0.1985 / 0.0733	0.201 / 0.032	43.29 / 6.20	16.597 / 3.430
Rotation (θ = 1°)	$Vaidya_{ae}$	0.0130 / 0.0187	0.080 / 0.008	17.83 / 1.61	6.406 / 0.835
	$Vaidya_{be}$	0.0878 / 0.0473	0.193 / 0.021	40.97 / 3.91	16.837 / 2.448
	Chaudhary	0.1312 / 0.1821	0.316 / 0.059	58.68 / 5.13	26.477 / 8.989
	$Proposed_{ae}$	-0.0004 / 0.0011	$0.042 / 3 \times 10^{-6}$	$10.48 / 7 \times 10^{-4}$	$2.774 / 3 \times 10^{-4}$
	$Proposed_{be}$	0.0300 / 0.0641	0.077 / 0.007	17.24 / 1.46	5.997 / 0.495
Rotation (θ = 2°)	$Vaidya_{ae}$	0.0174 / 0.0229	0.076 / 0.009	17.04 / 1.73	6.005 / 0.887
	$Vaidya_{be}$	0.0442 / 0.0353	0.184 / 0.020	38.35 / 3.74	16.471 / 2.241
	Chaudhary	0.1009 / 0.1779	0.363 / 0.068	58.62 / 5.58	33.654 / 8.835
	$Proposed_{ae}$	-0.0002 / 0.0020	$0.042 / 3 \times 10^{-6}$	$10.48 / 6 \times 10^{-4}$	$2.774 / 2 \times 10^{-4}$
	$Proposed_{be}$	0.0095 / 0.0461	0.077 / 0.006	16.53 / 1.21	6.087 / 0.397
Rotation (θ = 5°)	$Vaidya_{ae}$	0.0165 / 0.0202	0.078 / 0.008	17.54 / 1.48	6.250 / 0.755
	$Vaidya_{be}$	0.0050 / 0.0203	0.185 / 0.018	36.74 / 3.41	16.776 / 1.912
	Chaudhary	0.0783 / 0.1821	0.436 / 0.079	60.14 / 6.93	42.902 / 9.128
	$Proposed_{ae}$	-0.0002 / 0.0014	$0.042 / 3 \times 10^{-6}$	$10.48 / 5 \times 10^{-4}$	$2.774 / 2 \times 10^{-4}$
	$Proposed_{be}$	-0.0276 / 0.0179	0.092 / 0.008	17.54 / 1.40	7.572 / 0.630
Rotation (θ = 90°)	$Vaidya_{ae}$	0.0172 / 0.0174	0.077 / 0.008	17.38 / 1.48	6.174 / 0.782
	$Vaidya_{be}$	0.0099 / 0.0327	0.092 / 0.014	21.01 / 2.60	7.319 / 1.537
	Chaudhary	0.8880 / 0.0796	0.236 / 0.092	71.33 / 22.53	5.547 / 3.441
	$Proposed_{ae}$	0.0002 / 0.0020	$0.042 / 3 \times 10^{-6}$	$10.48 / 6 \times 10^{-4}$	$2.774 / 3 \times 10^{-4}$
	$Proposed_{be}$	-7×10^{-5} / 0.0080	0.044 / 0.001	11.63 / 0.50	2.820 / 0.067

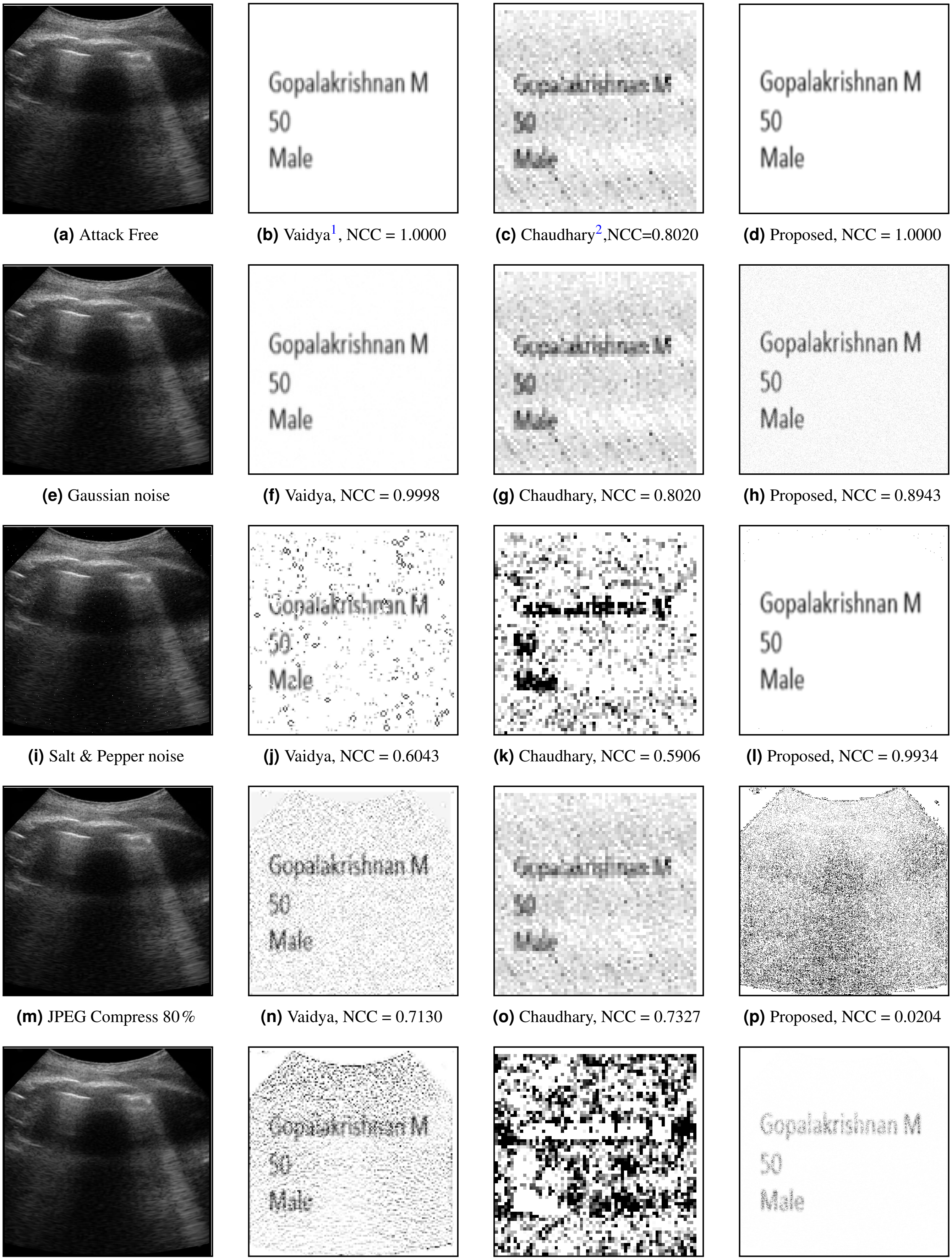

(a) Attack Free **(b)** Vaidya[1], NCC = 1.0000 **(c)** Chaudhary[2],NCC=0.8020 **(d)** Proposed, NCC = 1.0000

(e) Gaussian noise **(f)** Vaidya, NCC = 0.9998 **(g)** Chaudhary, NCC = 0.8020 **(h)** Proposed, NCC = 0.8943

(i) Salt & Pepper noise **(j)** Vaidya, NCC = 0.6043 **(k)** Chaudhary, NCC = 0.5906 **(l)** Proposed, NCC = 0.9934

(m) JPEG Compress 80 % **(n)** Vaidya, NCC = 0.7130 **(o)** Chaudhary, NCC = 0.7327 **(p)** Proposed, NCC = 0.0204

(q) Median Filter (3×3) **(r)** Vaidya, NCC = 0.4645 **(s)** Chaudhary, NCC = -0.2311 **(t)** Proposed, NCC = 0.6559

Figure S7. Visual comparison of extracted watermarks using the LUI dataset. First column represents the watermarked host images after the respective attack. Gaussian noise parameters are $\mu = 0.01$, $\sigma^2 = 0.002$, Salt and Pepper noise parameter is noise density = 0.002. Refer to the caption of Fig. S8 for technical details regarding watermark resolutions.

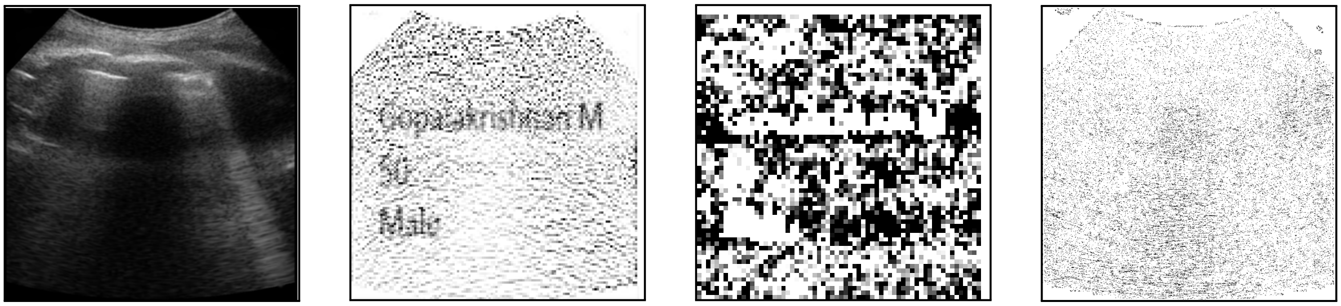

(a) Mean Filter (3×3) **(b)** Vaidya[1], NCC = 0.3831 **(c)** Chaudhary[2],NCC=-0.2607 **(d)** Proposed, NCC = 0.0169

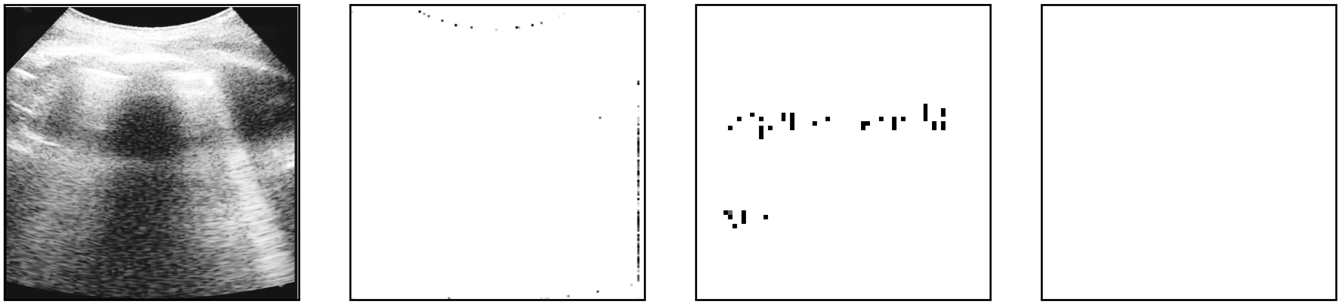

(e) Histogram Equalisation **(f)** Vaidya, NCC = -0.0127 **(g)** Chaudhary, NCC = 0.4535 **(h)** Proposed, NCC = 0

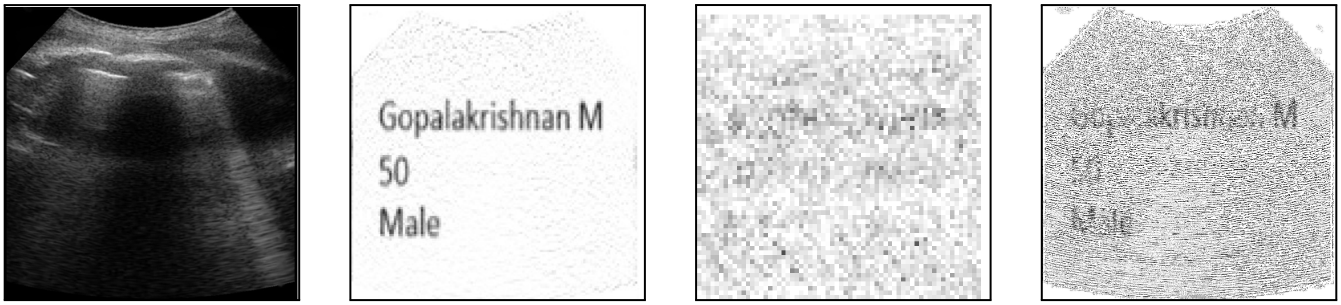

(i) Scaling attack (2× then 0.5×) **(j)** Vaidya, NCC = 0.9693 **(k)** Chaudhary, NCC = 0.2925 **(l)** Proposed, NCC = 0.1377

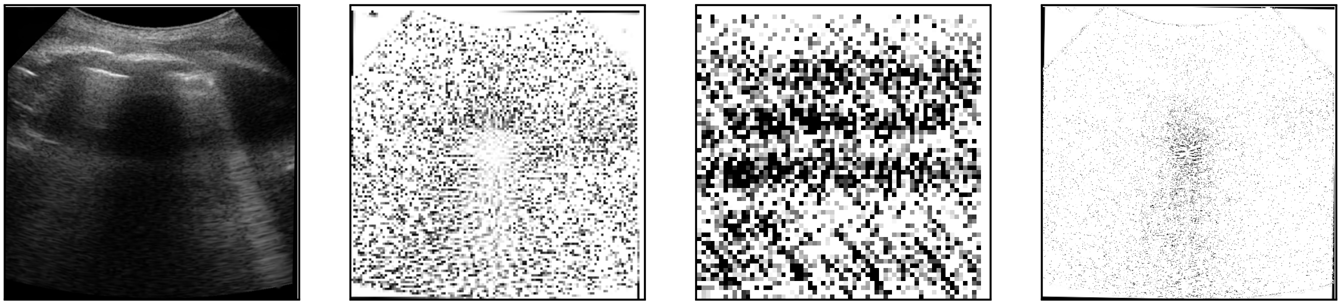

(m) Rotation 1° **(n)** Vaidya, NCC = 0.0748 **(o)** Chaudhary, NCC = 0.2731 **(p)** Proposed, NCC = -0.0127

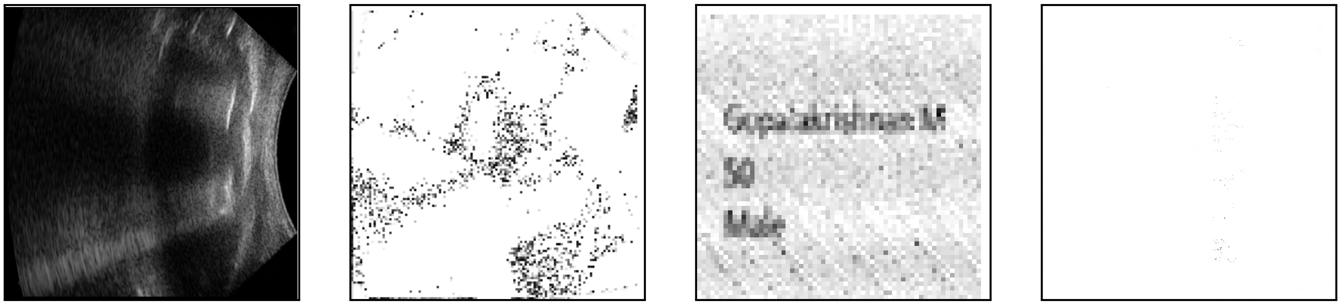

(q) Rotation 90° **(r)** Vaidya, NCC = 0.0107 **(s)** Chaudhary, NCC = 0.8020 **(t)** Proposed, NCC = 0.0051

Figure S8. Visual comparison of extracted watermarks using the LUI dataset. First column represents the watermarked host images after the respective attack. For the proposed method, the extraction utilises the native 512×512 watermark resolution, while Vaidya's[1] and Chaudhary's[2] extractions correspond to their native 128×128 and 64×64 dimensions, respectively. NCC values are calculated on these native dimensions without interpolation to prevent artificial smoothing bias.

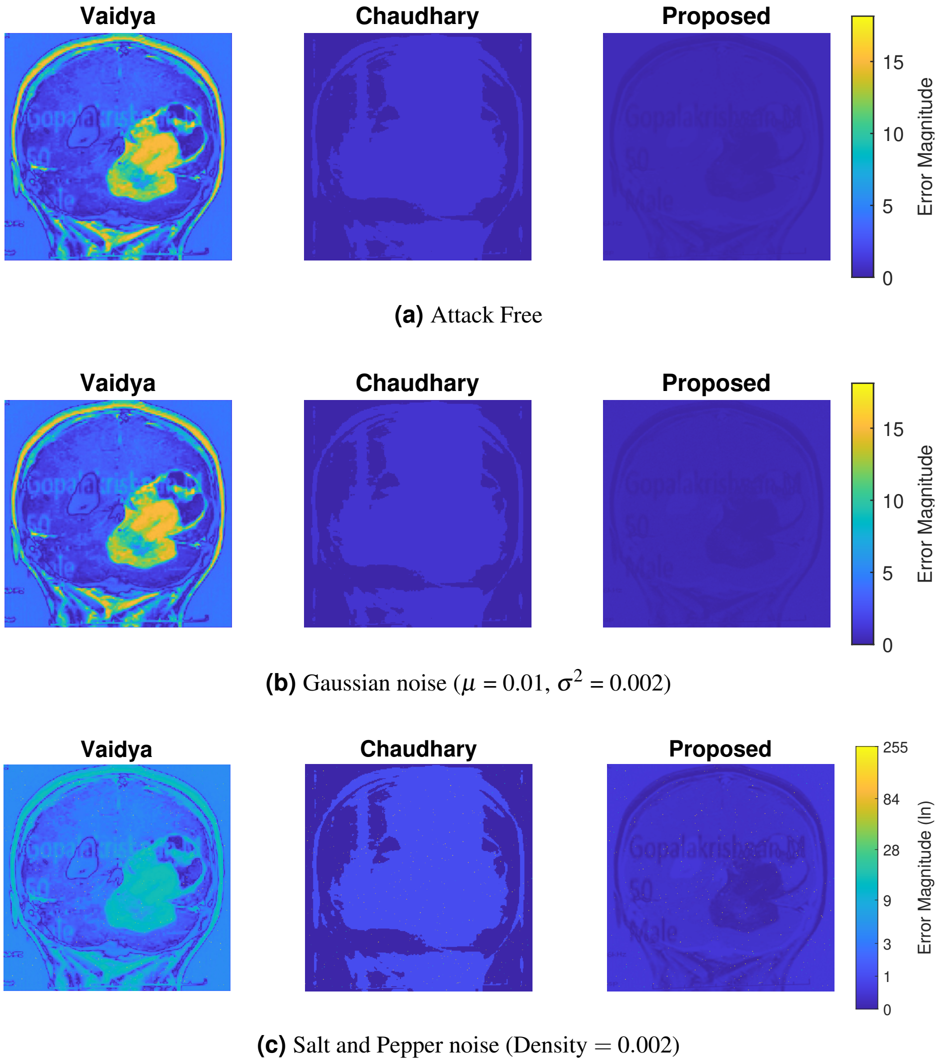

Figure S9. Residual difference maps for the proposed method compared to SOTA methods on the BMIBTD dataset. Subfigure (a) confirms the minimal residual footprint and near-total visual sparsity, while (b) and (c) demonstrate the stochastic distribution of error under high-robustness scenarios for the proposed method.

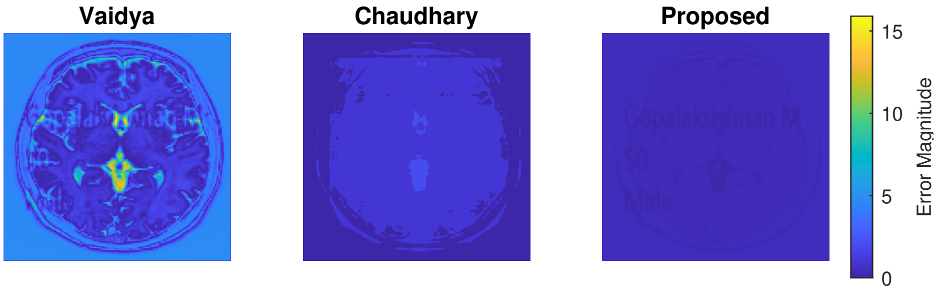

(a) Attack Free

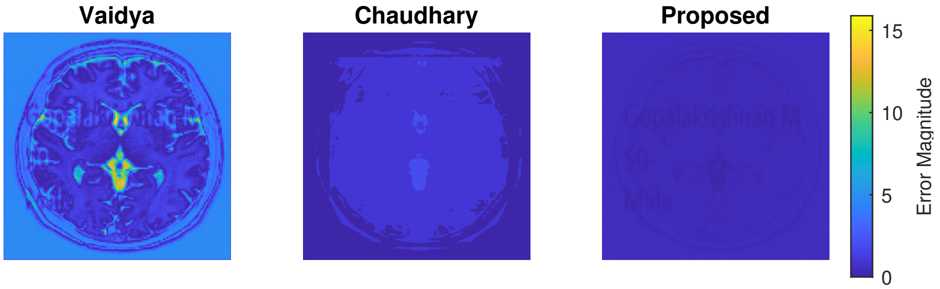

(b) Gaussian noise ($\mu = 0.01$, $\sigma^2 = 0.002$)

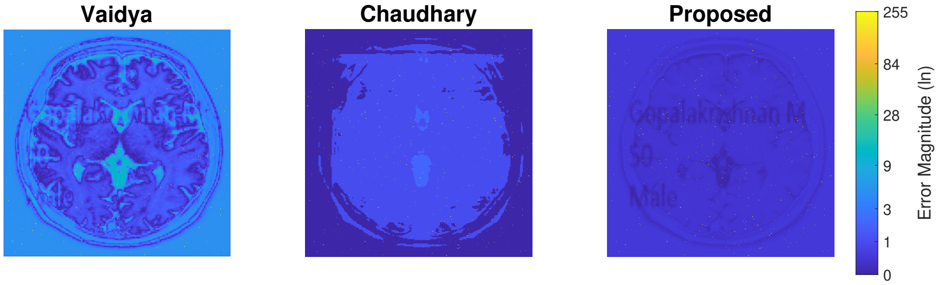

(c) Salt and Pepper noise (Density = 0.002)

Figure S10. Residual difference maps for the proposed method compared to SOTA methods on the BMIBTD dataset. Subfigure (a) confirms the minimal residual footprint and near-total visual sparsity, while (b) and (c) demonstrate the stochastic distribution of error under high-robustness scenarios for the proposed method.

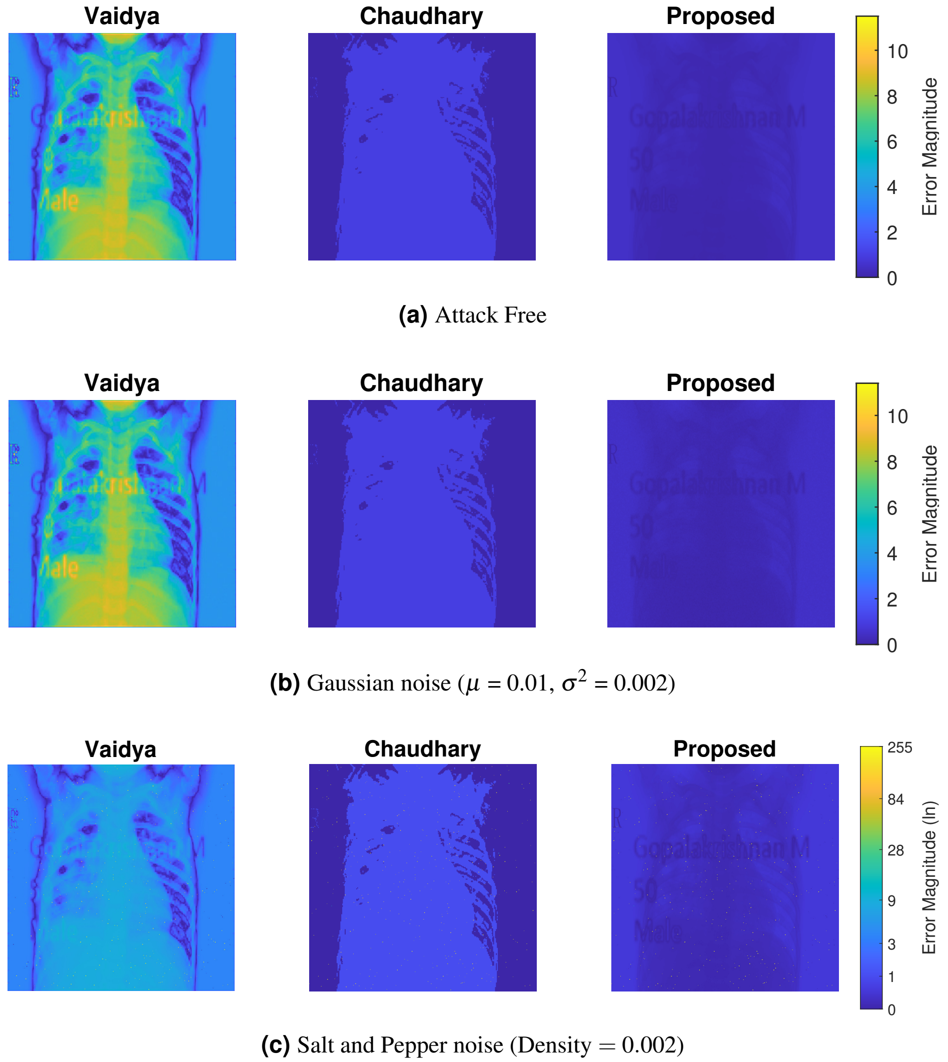

Figure S11. Residual difference maps for the proposed method compared to SOTA methods on the CXIP dataset. Subfigure (a) confirms the minimal residual footprint and near-total visual sparsity, while (b) and (c) demonstrate the stochastic distribution of error under high-robustness scenarios for the proposed method.

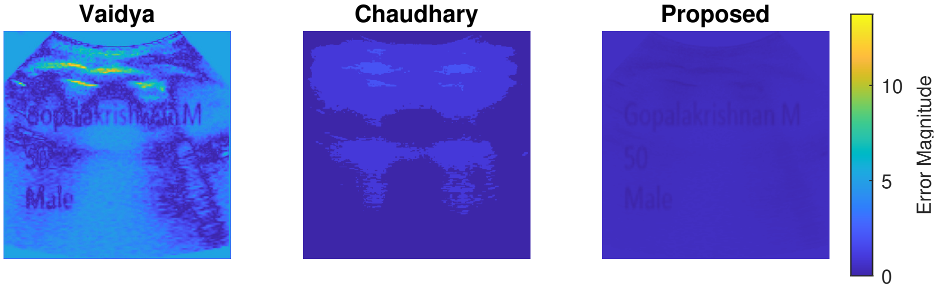

(a) Attack Free

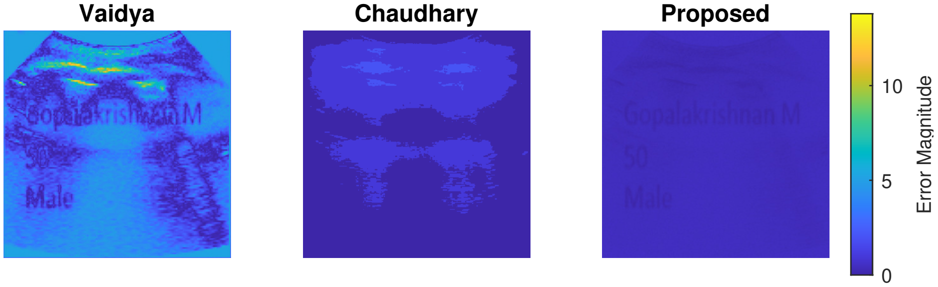

(b) Gaussian noise ($\mu = 0.01$, $\sigma^2 = 0.002$)

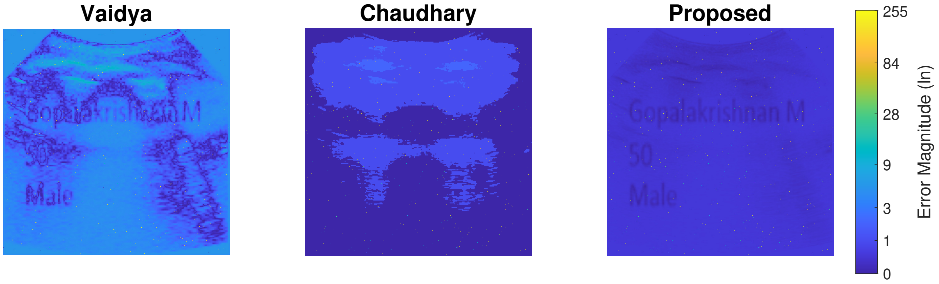

(c) Salt and Pepper noise (Density = 0.002)

Figure S12. Residual difference maps for the proposed method compared to SOTA methods on the LUI dataset. Subfigure (a) confirms the minimal residual footprint and near-total visual sparsity, while (b) and (c) demonstrate the stochastic distribution of error under high-robustness scenarios for the proposed method.

Table S7. Comparison of Security Performance Metrics. Proposed$_{ne}$ refers to values for proposed work without encryption. Proposed$_x$ and Proposed$_r$ represent perturbations in x_{seed} ($\Delta = 10^{-8}$) and r_{param} ($\Delta = 10^{-15}$) respectively. (Values: Mean / Std., NPCR and UACI in %, Correlation r_{xy} for Horizontal (H), Vertical (V), and Diagonal (D) directions.)

Data	Method	NPCR$_{sec}$ (%)	UACI$_{sec}$ (%)	Entropy	Corr (H)	Corr (V)	Corr (D)
CXIP	Vaidya's[1]	4×10^{-4} / 0	2×10^{-6} / 0	7.132 / 0.201	0.148 / 0.086	0.429 / 0.083	0.650 / 0.060
	Chaudhary's[2]	- / -	- / -	7.232 / 0.215	0.994 / 0.002	0.996 / 0.002	0.992 / 0.004
	Proposed$_{ne}$	- / -	- / -	7.213 / 0.213	0.994 / 0.002	0.996 / 0.002	0.992 / 0.004
	Proposed$_x$	99.99 / 7×10^{-4}	17.329 / 0.099	7.683 / 0.048	0.032 / 0.021	0.033 / 0.019	0.128 / 0.029
	Proposed$_r$	99.99 / 7×10^{-4}	17.349 / 0.198	7.683 / 0.048	0.032 / 0.021	0.033 / 0.019	0.128 / 0.029
LUI	Vaidya's[1]	4×10^{-4} / 0	2×10^{-6} / 1×10^{-21}	6.158 / 0.801	0.253 / 0.079	0.476 / 0.072	0.643 / 0.057
	Chaudhary's[2]	- / -	- / -	6.012 / 0.923	0.994 / 0.001	0.986 / 0.004	0.981 / 0.005
	Proposed$_{ne}$	- / -	- / -	5.964 / 0.906	0.994 / 0.001	0.985 / 0.004	0.981 / 0.005
	Proposed$_x$	99.99 / 7×10^{-4}	18.526 / 1.167	7.551 / 0.071	0.024 / 0.016	0.083 / 0.029	0.054 / 0.030
	Proposed$_r$	99.99 / 7×10^{-4}	18.517 / 1.177	7.551 / 0.071	0.024 / 0.016	0.083 / 0.029	0.054 / 0.030

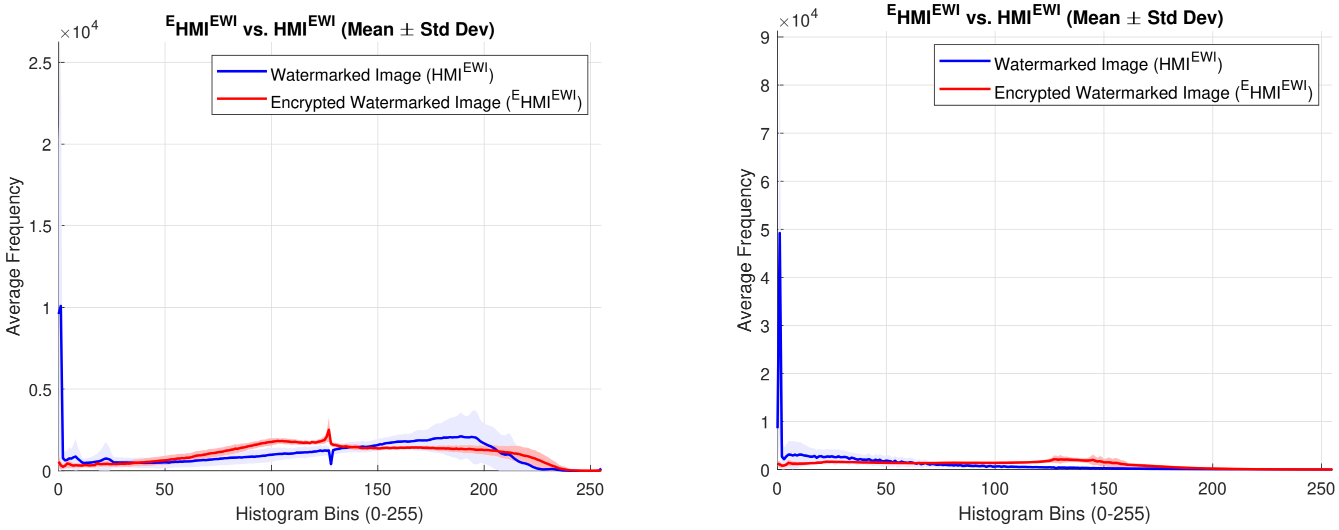

(a) CXIP Dataset

(b) LUI Dataset

Figure S13. Visualisation of histogram flattening effect in the proposed method. The mean and standard deviation of the histograms of watermarked images and encrypted watermarked images for the respective datasets are plotted.

References

1. Vaidya, S. P. Fingerprint-based robust medical image watermarking in hybrid transform. *The Vis. Comput.* **39**, 2245–2260 (2023).
2. Chaudhary, H., Garg, P. & Vishwakarma, V. Enhanced medical image watermarking using hybrid dwt-hmd-svd and arnold scrambling. *Sci. Reports* **15**, DOI: 10.1038/s41598-025-94080-4 (2025).
